# Homoconjugation-induced fluorescence enhancement in *C*_3v_-symmetric pyrene- and phenanthrene-embedded triptycenes

**DOI:** 10.1039/d6sc07032a

**Published:** 2026-11-10

**Authors:** Puyi Huang, Yu Watanabe, Masahiro Yamashina, Shinji Toyota

**Affiliations:** a Department of Chemistry, School of Science, Institute of Science Tokyo 2-12-1 Ookayama Meguro-ku Tokyo 152-8551 Japan yamashina.m.7af1@m.isct.ac.jp toyota.s.b178@m.isct.ac.jp

## Abstract

π-Extension of triptycene provides an effective strategy for constructing three-dimensional π-conjugated molecules featuring through-space homoconjugation. However, because π-extended triptycenes composed solely of polycyclic aromatic hydrocarbon (PAH) units have rarely been explored, the intrinsic effects of homoconjugation on embedded PAH units remain poorly understood. Herein, we report the synthesis of *C*_3v_-symmetric pyrene- and phenanthrene-embedded triptycenes as model systems for investigating the intrinsic effects of homoconjugation. The π-extended triptycenes were synthesized by Negishi cross-coupling followed by Pt-catalyzed cyclization and fully characterized by spectroscopic and single-crystal X-ray diffraction analyses. DFT calculations revealed that the homoconjugation promotes delocalization of the frontier molecular orbitals over the molecular framework and narrows the HOMO–LUMO gap, leading to bathochromic shifts in the absorption and emission bands. More importantly, TD-DFT calculations revealed that homoconjugation substantially increased the allowed character of the S_0_←S_1_ transition in the parent PAHs, resulting in significantly increased oscillator strengths, radiative decay rates, and fluorescence quantum yields. These findings establish homoconjugation as an effective molecular design principle for tuning the electronic structure and optical properties of three-dimensional PAH-based π-conjugated molecules.

## Abstract 2

Homoconjugative interactions among three pyrene or phenanthrene units in a *C*_3v_-symmetric triptycene framework induce bathochromic shifts in absorption and emission, accompanied by enhanced fluorescence quantum yields.

## Graphical abstract

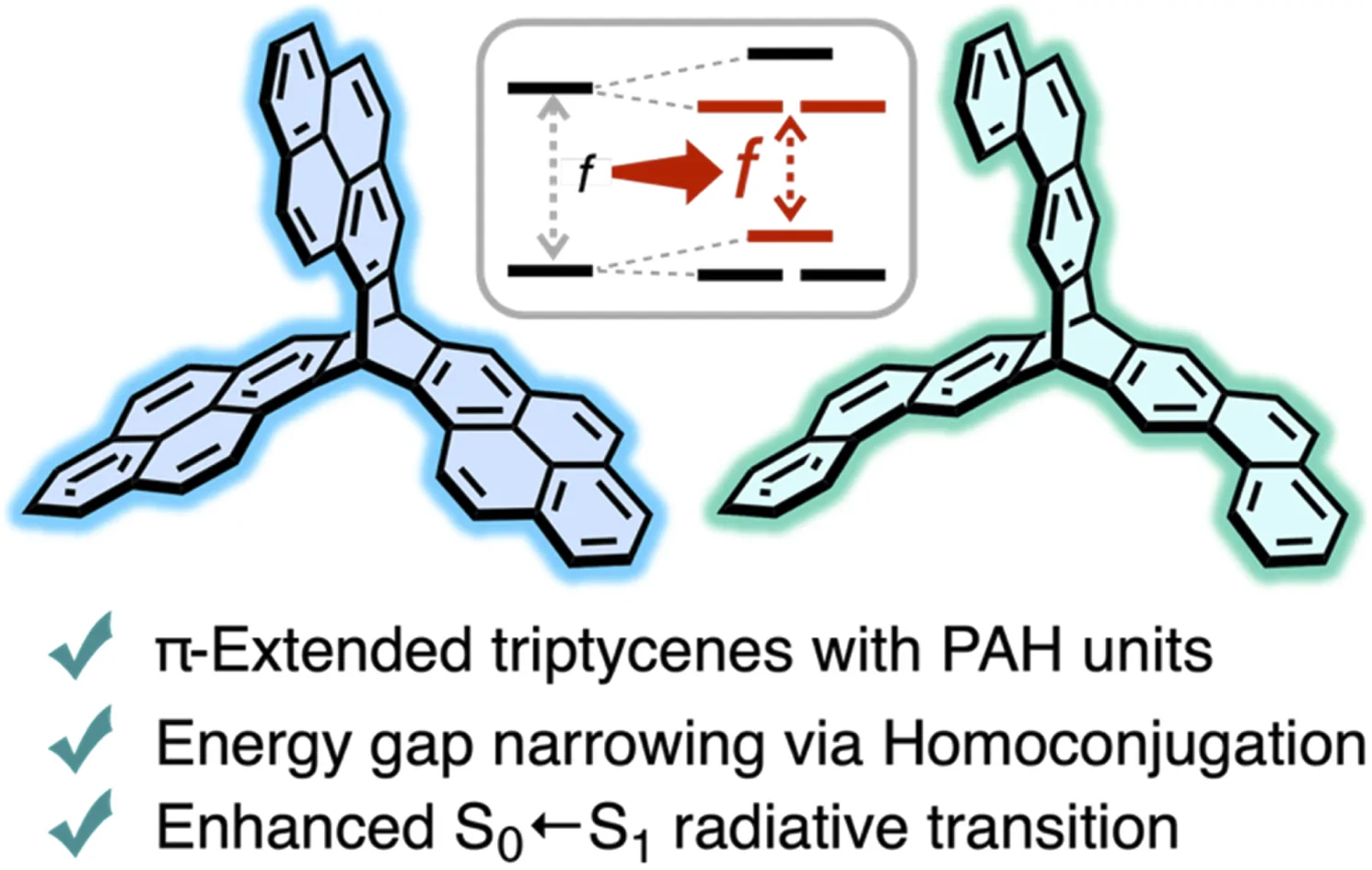

## Introduction

Homoconjugation refers to the delocalization of electrons through orbital interactions between two π-electron systems separated by a non-conjugating group, such as a CH_2_ group.^1–3^ Homoconjugated three-dimensional molecules possess unique electronic structures together with characteristic physicochemical properties (*e.g.*, optical properties and multidirectional charge transport^4–12^). Among them, triptycene having a rigid framework composed of three benzene rings has been extensively investigated as a prototypical homoconjugated scaffold (Fig. 1a, left).^13–16^ More recently, π-extension of triptycenes has emerged as an effective strategy for constructing three-dimensional π-conjugated systems *via* the cooperative interplay of conventional through-bond conjugation and through-space homoconjugation (Fig. 1a, right).^17–25^ Most reported π-extended triptycenes incorporate heteroatom-containing donor–acceptor chromophores, enabling optical functions such as through-space charge transfer^26–28^ and thermally activated delayed fluorescence (TADF).^29–32^ However, since the frontier molecular orbitals are largely governed by heteroatom-derived electronic effects, the intrinsic effect of homoconjugation on embedded π-conjugated chromophores remains poorly understood.

**Fig. 1:**
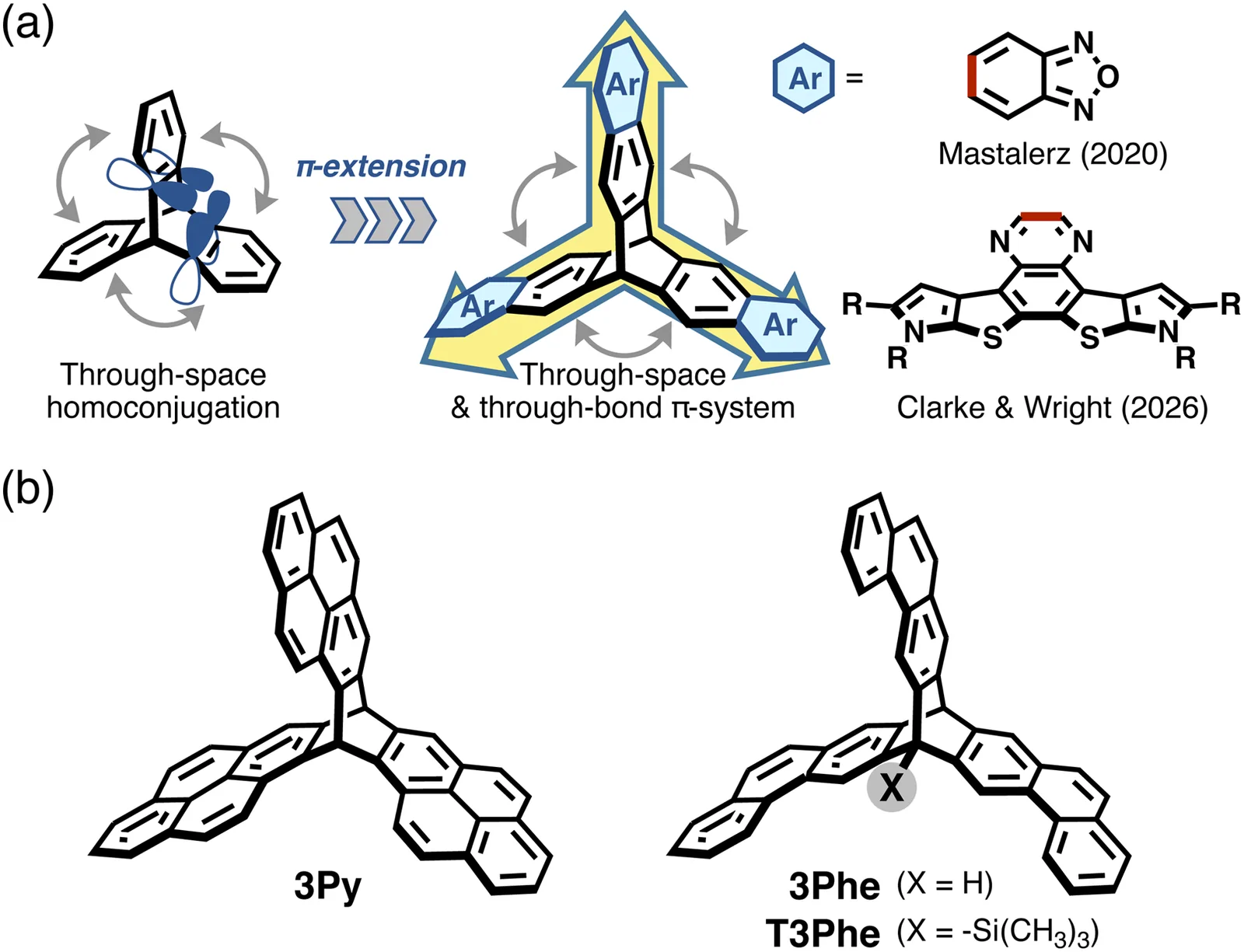
Concept of this study. (a) Homoconjugation in triptycene and schematic representation of the previously reported π-extended triptycenes. The bonds highlighted in red in the structure indicate the positions at which the π-conjugated units are fused to the triptycene framework. (b) Chemical structures of π-extended triptycenes 3Py, 3Phe, and T3Phe investigated in this study.

Polycyclic aromatic hydrocarbons (PAHs) provide an ideal platform for addressing this issue because their electronic structures are well established and are free from heteroatom-derived electronic effects.^33,34^ Nevertheless, there have been only a few reports regarding PAH-embedded π-extended triptycenes because of their synthetic limitations.^35–40^ Therefore, the influence of homoconjugation on the electronic structures and optical properties of PAHs remains unclear. Herein, we report the synthesis of the *C*_3v_-symmetric π-extended triptycenes 3Py and 3Phe, embedding pyrene and phenanthrene units, respectively (Fig. 1b). With its characteristic *C*_3v_ structure, 3Py forms dual-pore crystals in the solid state. DFT calculations revealed that homoconjugation promotes the frontier orbital delocalization and narrows the HOMO–LUMO gap. As a consequence, the π-extended triptycene derivatives exhibit pronounced changes in the absorption and emission together with substantial enhancements of the fluorescence quantum yields. These findings demonstrate that homoconjugation provides an effective strategy for tuning electronic structures and emissive properties of PAHs, thereby establishing a new molecular design of three-dimensional π-conjugated molecules.

## Results and discussion

### Synthesis of π-extended triptycenes 3Py and 3Phe

The *C*_3v_-symmetric π-extended pyrene-embedded triptycene 3Py was synthesized in three steps from 2,7,14-tribromotriptycene 3Br (ref. 41) and 2-bromo-1,3-bis[2-(trimethylsilyl)ethynyl]benzene (A) (Scheme 1 and Fig. S1). Compound A was prepared from 1,3-dichlorobenzene *via* a slightly modified Hart reaction according to the protocol reported by Ito *et al.*^42^ Treatment of A with *n*-BuLi followed by ZnCl_2_ afforded the corresponding organozinc reagent A_Zn_, which subsequently underwent Negishi cross-coupling^43^ with 3Br to give 1a in 79% yield. Subsequent deprotection of the trimethylsilyl groups with tetrabutylammonium fluoride (TBAF) furnished 1b in 99% yield. The final step involved a Pt-catalyzed intramolecular cycloisomerization. In contrast to previously reported conditions,^44–47^1b predominantly underwent oligomerization rather than the desired cycloisomerization. After extensive optimization of the reaction conditions, we found that the cycloisomerization worked best under dilute conditions (*ca.* 1 mM), affording 3Py in 10% yield after purification by silica gel column chromatography and recycling GPC. The synthesis of the phenanthrene-incorporated triptycene 3Phe was carried out from 3Br using 1-bromo-2-(trimethylsilylethynyl)benzene (B)^48^ following the same procedure as 3Py. Although the cycloisomerization of 2b proceeded more efficiently than that of 1b, it afforded an inseparable mixture of two regioisomers 3Phe (*C*_3v_) and 3Phe′ (*C*_s_) in a 2.0 : 1.0 molar ratio in 33% combined yield. These isomers differ in the cyclization regioselectivity: all three phenanthrene units in 3Phe are formed *via* cyclization at the 3-position, whereas one unit in 3Phe′ is cyclized at the 1-position. Therefore, to achieve regioselective cycloisomerization, we next introduced steric control into the precursor. Indeed, the 2-ethynylphenyl derivative 3b, which was prepared from 9-trimethylsilyl-substituted T3Br,^41^ successfully underwent a selective cycloisomerization, affording the *C*_3v_-symmetric product T3Phe in 57% yield (Scheme 1b). Subsequent removal of the trimethylsilyl group with TBAF furnished the desired 3Phe.

**Scheme 1:**
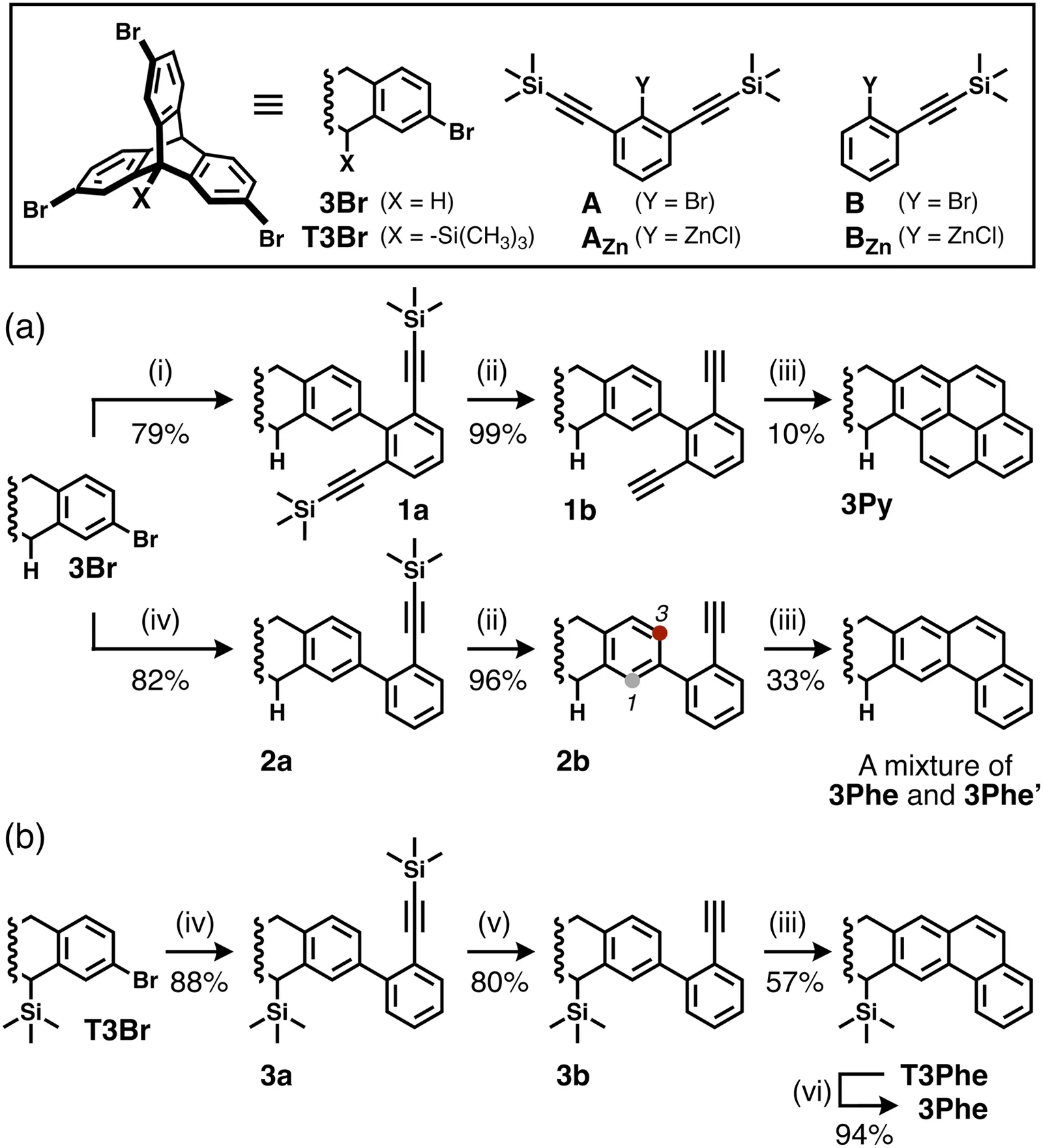
Synthetic route of pyrene/phenanthrene-incorporated triptycene. (a) Synthesis of 3Py and 3Phe, and (b) selective synthesis of 3Phe*via* the formation of T3Phe: (i) A_Zn_, PdCl_2_(CH_3_CN)_2_, XPhos, THF, reflux; (ii) TBAF, THF, r.t.; (iii) PtCl_2_, P(C_6_F_5_)_3_, toluene, reflux; (iv) B_Zn_, PdCl_2_(CH_3_CN)_2_, SPhos, THF, reflux; (v) TBAF, THF, −40 °C; (vi) TBAF, THF, 76 °C.

The ^1^H NMR spectra of 3Py and 3Phe are shown in Fig. 2a, and all signals were assigned by 2D NMR analyses (see SI). Owing to the *C*_3v_-symmetric structure, the bridgehead proton signals *H*_a_ and *H*_b_ appeared at different chemical shifts, corresponding to the inner and outer positions. For instance, the inner proton *H*_a_ of 3Phe appeared at 6.18 ppm, which was slightly downfield shifted relative to the outer proton *H*_b_ (Δ*δ* = +0.2 ppm) (Fig. 2a, top). Surprisingly, in the case of 3Py, the *H*_a_ signal appeared at 8.68 ppm despite being attached to an sp^3^-carbon, representing a substantial downfield shift relative to *H*_b_ (Δ*δ* = +2.0 ppm) (Fig. 2a, bottom). This shift is particularly remarkable considering that the corresponding bridgehead proton signal of triptycene typically appears at 5.45 ppm in CDCl_3_.^49^ Notably, the bridgehead carbon *C*_a_ (40.6 ppm) exhibited a substantial upfield shift in the ^13^C NMR spectrum relative to *C*_b_ (56.7 ppm, Δ*δ* = −16 ppm, Fig. 2b). To obtain insight into the origin of these chemical shift differences, the gauge-invariant atomic orbitals (GIAO) and 2D nuclear-independent chemical shift (NICS) calculations were performed (Fig. 2c and Table S3). The GIAO calculations showed good agreement with the experimentally observed chemical shifts, reproducing both the pronounced downfield shift of *H*_a_ and the upfield shift of *C*_a_. Furthermore, the 2D NICS maps revealed an obvious deshielding and shielding regions around the *H*_a_ and the *C*_a_, respectively (Fig. 2d). In addition, the *C*_a_–*H*_a_ bond of 3Py in the optimized structure was slightly shorter than that in triptycene (1.083 *vs.* 1.091 Å; Fig. S6), indicating local steric compression around the inner bridgehead position.^45–47^ A slight shortening of the *C*_j_–*H*_j_ bonds was also observed relative to the neighboring *C*_i_–*H*_i_ bonds (1.084 Å *vs.* 1.086 Å; Fig. S6). These results suggest that the unusual chemical shifts of *H*_a_ and *C*_a_ arise from the combined effects of magnetic shielding/deshielding by the neighboring aromatic panels and steric compression at the inner bridgehead position.

**Fig. 2:**
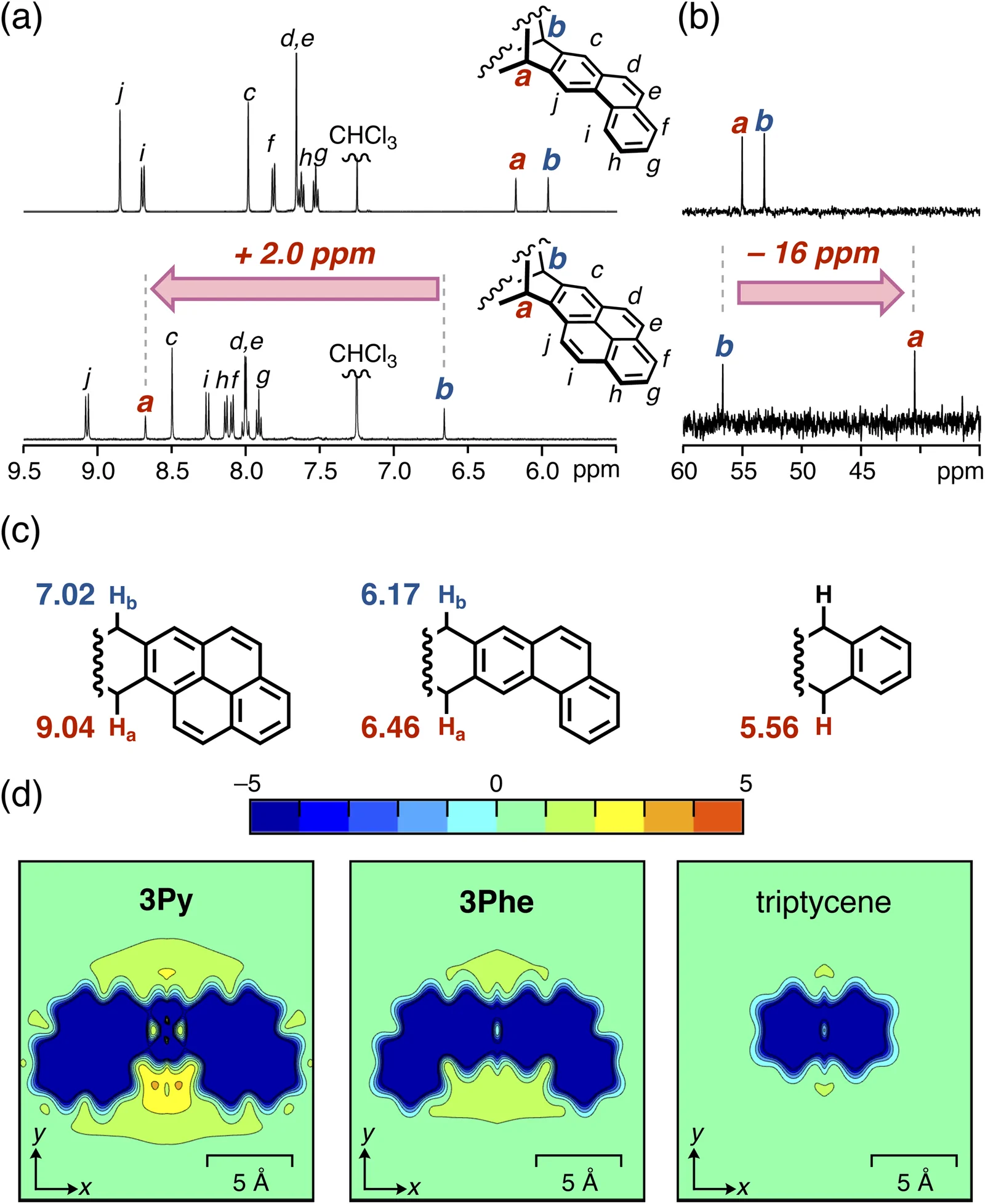
NMR analysis of *C*_3v_-symmetric triptycenes. (a) ^1^H NMR spectra (500 MHz, CDCl_3_, 298 K) and (b) ^13^C NMR spectra (125 MHz, CDCl_3_, 298 K) of 3Py and 3Phe. (c) Calculated chemical shifts and (d) 2D-NICS maps of 3Py, 3Phe, and triptycene. Left (3Py), center (3Phe), and right (triptycene). The 2D-NICS maps were generated using NICS(1)_iso_ values calculated on 120°-folded cross-sectional planes located 1 Å from the two aromatic panels of the triptycene surface. The calculated three-dimensional data were projected onto a two-dimensional plane by omitting the *z*-axis component. The calculations were performed at the HF/6-311+G(d,p) (PCM, CHCl_3_) level^50,51^ for (c) and at the B3LYP-D3(BJ)/6-31G(d,p) level for (d), using structures optimized at the B3LYP-D3(BJ)/6-31G(d,p) (PCM, CHCl_3_) level.

### Single crystal X-ray diffraction analysis

Single-crystal X-ray diffraction analyses (SXRD) unambiguously revealed the formation of *C*_3v_-symmetric π-extended triptycenes (Fig. 3a and S2–S5). The interpanel angles between the fused polyaromatic moieties were evaluated. The angles in 3Phe and T3Phe were approximately 120°, which is comparable to those of the optimized structures (Fig. S6). In contrast, the corresponding angles in 3Py deviated from the ideal values, showing values of 114°, 115°, and 132°, respectively, owing to crystalline packing effects. These results encouraged us to investigate the packing structure. Surprisingly, single crystals of 3Py obtained from CHCl_3_ and MeOH (*i.e.*3Py·CHCl_3_) formed a dual-pore crystal,^52^ containing two distinct-sized pores in a crystal lattice (Fig. 3b and d). The larger and smaller pores exhibited effective diameters of 5.9–8.1 Å and 5.2–6.0 Å, respectively, and were present in a 2 : 1 ratio. Each pore was constructed from six 3Py molecules arranged in an anti-parallel orientation. Whereas intermolecular π–π and CH–π interactions were found among the 3Py molecules in the large pore, only CH–π interactions were observed in the small pore. Although CHCl_3_ molecules occupied the pores, the low solvent occupancy observed in the CH_2_Cl_2_ crystal suggested that the dual-pore structure may be preserved even after removal of the solvents. Single crystals of 3Py were also obtained from C_6_F_6_/MeOH (*i.e.*, 3Py·C_6_F_6_), affording a similar crystal system. Strikingly, three electron-deficient C_6_F_6_ molecules were fully clathrated within the larger pore, as confirmed by SXRD analysis (Fig. 3c and e). As a consequence of this clathration, the effective diameter of the small pores slightly increased to 5.9–6.8 Å relative to that in 3Py·CHCl_3_, resulting in the formation of a single-pore crystal. In contrast, 3Phe adopted a simple close-packed structure without a pore (Fig. S4 and S5). These findings thus imply that such *C*_3v_-symmetric π-extended triptycenes can form dual-pore crystals through the directional assembly enabled by *C*_3v_-symmetry.

**Fig. 3:**
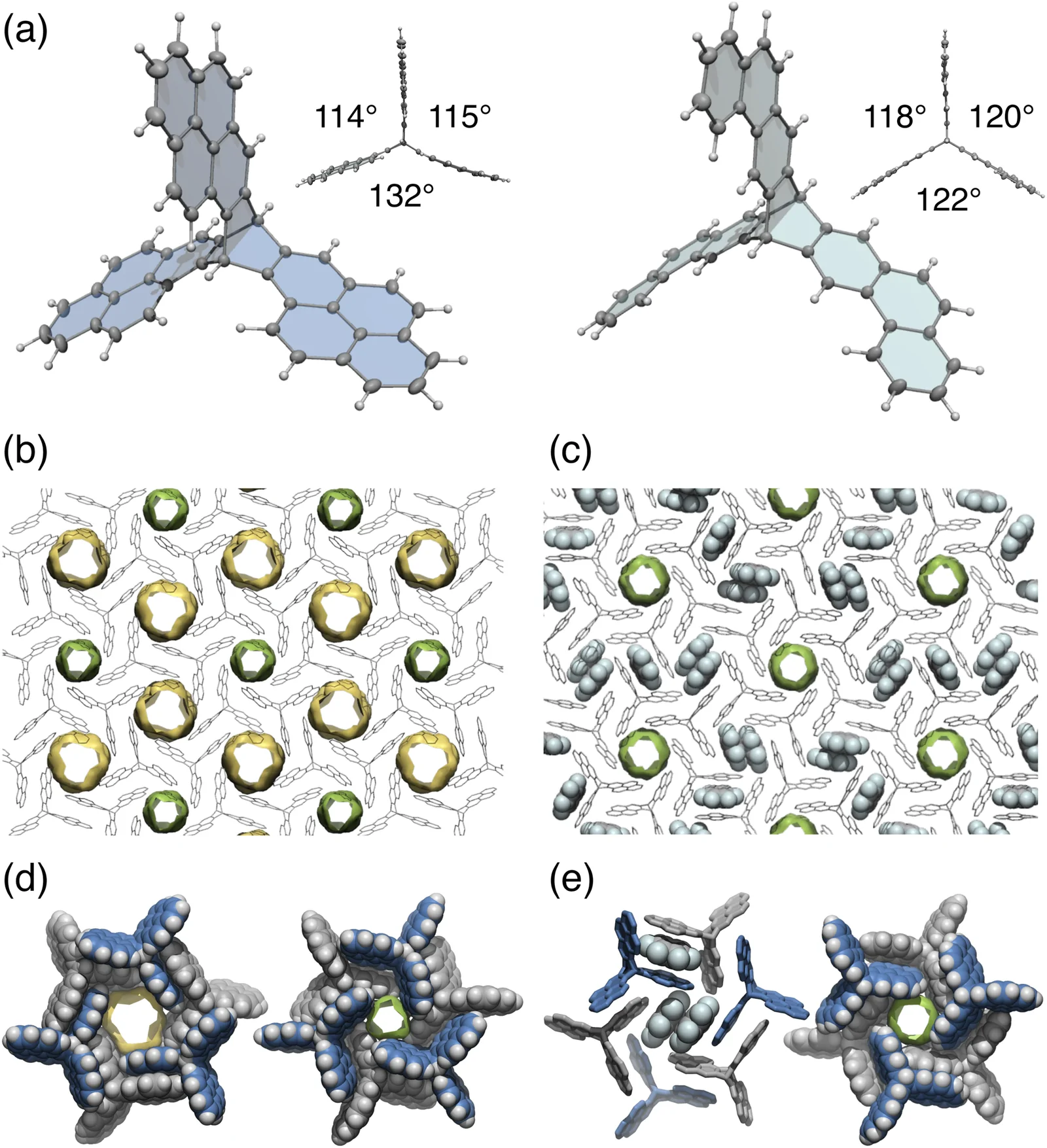
Single-crystal X-ray diffraction analysis. (a) ORTEP drawings of 3Py and 3Phe. Thermal ellipsoids are drawn at 50% probability. Interpanel angles were defined as the angular sectors between the PAH centroids projected onto a plane perpendicular to the bridgehead C–C axis. Crystalline packing structures of 3Py recrystallized from (b) CHCl_3_/MeOH and (c) C_6_F_6_/MeOH. (d and e) Representative assemblies of 3Py defining the pore and a related site. The solvent molecules within the pores are omitted for clarity. The pores are colored yellow or green.

### DFT calculations

To evaluate the potential of homoconjugation-mediated electronic communication, we performed DFT calculations (Fig. 4, S7 and S8). Homoconjugative interactions among the three PAH units resulted in orbital splitting into a nondegenerate orbital and a pair of degenerate orbitals. In 3Py, the degenerate LUMO and LUMO+1 (−1.71 eV) were stabilized relative to the LUMO of pyrene (Py) (−1.57 eV). This stabilization arises from bonding orbital interactions between the pyrene moieties across the bicyclic scaffold. In contrast, the HOMO of 3Py (−5.17 eV) was destabilized relative to that of Py (−5.42 eV) because of antibonding orbital interactions between the pyrene moieties. Consequently, the HOMO–LUMO gap was narrowed from 3.84 eV for Py to 3.47 eV for 3Py. Similar orbital splitting was observed for 3Phe, in which the three orbitals derived from the LUMO of phenanthrene (Phe) corresponded to the degenerate LUMO/LUMO+1 and the higher-energy LUMO+4 of 3Phe. Nucleus-independent chemical shift (NICS) calculations revealed no significant changes in the local aromaticity (Table S3b). These results suggest that the orbital energy changes arise mainly from homoconjugative interactions among the PAH units through the triptycene scaffold.

**Fig. 4:**
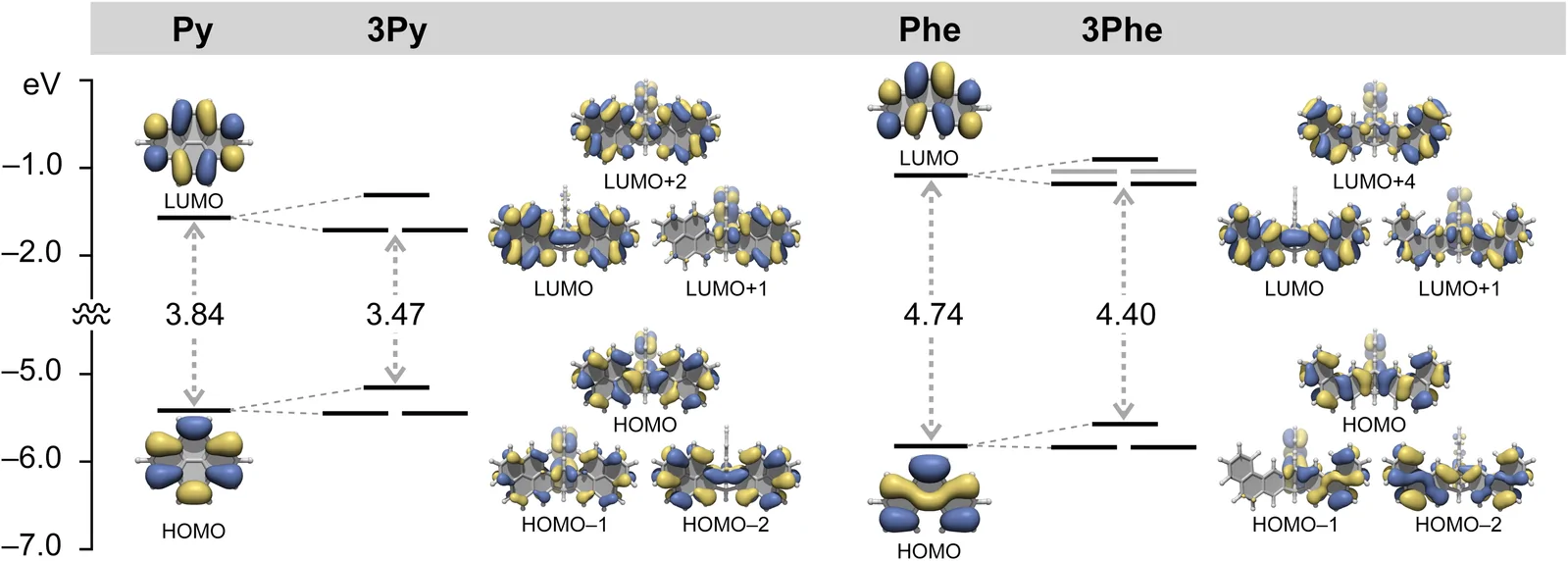
Frontier molecular orbitals and energy diagrams. Structural optimizations were carried out by DFT at the B3LYP-D3(BJ)/6-31G(d,p) (PCM, CHCl_3_) level. The LUMO+2 and LUMO+3 levels of 3Phe are shown in gray.

### Physical properties of π-extended triptycenes

The DFT calculations prompted us to investigate the optical and electrochemical properties of π-extended triptycene derivatives. In the UV-vis spectra, both 3Py and 3Phe exhibited significantly red-shifted absorption bands relative to their corresponding aromatic subunits (Fig. 5a and b and Table 1). This bathochromic shift is consistent with the reduced HOMO–LUMO gaps calculated by DFT calculations. Interestingly, their spectral patterns and molar absorption coefficients (*ε*) differ from those of Py and Phe, indicating that the absorption properties are not simply additive. 3Py and 3Phe displayed weak absorption bands at longer wavelengths (387 nm for 3Py, and 352 and 335 nm for 3Phe). To assign these absorptions, TD-DFT calculations and natural transition orbital (NTO) analyses were performed. The calculated excitation energies varied depending on the functional (Fig. S10). Accordingly, the following discussion focuses on the character and relative ordering of the electronic transitions. For 3Py, the nearly degenerate S_1_ and S_2_ states are represented by HOMO→LUMO and HOMO→LUMO+1 transitions, respectively, indicating that the lowest-energy absorption originates from the pyrene-derived S_1_ state stabilized through homoconjugation (Fig. S7 and S9). In contrast, the S_0_→S_1_ transition of Phe is forbidden,^53^ whereas the S_0_→S_2_ transition is allowed (Fig. 5c and S8). Although the nearly degenerate S_1_ and S_2_ states of 3Phe contained multiple molecular orbital contributions, NTO analysis revealed two dominant excitation patterns resembling the HOMO→LUMO and HOMO→LUMO+1 excitations (Fig. 5d and S8). Both S_1_ and S_2_ of 3Phe were optically allowed (*f ca.* 0.14). These results suggest that the lowest-energy absorption of 3Phe originates from homoconjugation-induced stabilization of the originally allowed S_2_ state of Phe, rather than from brightening of its originally forbidden S_1_ state.

**Fig. 5:**
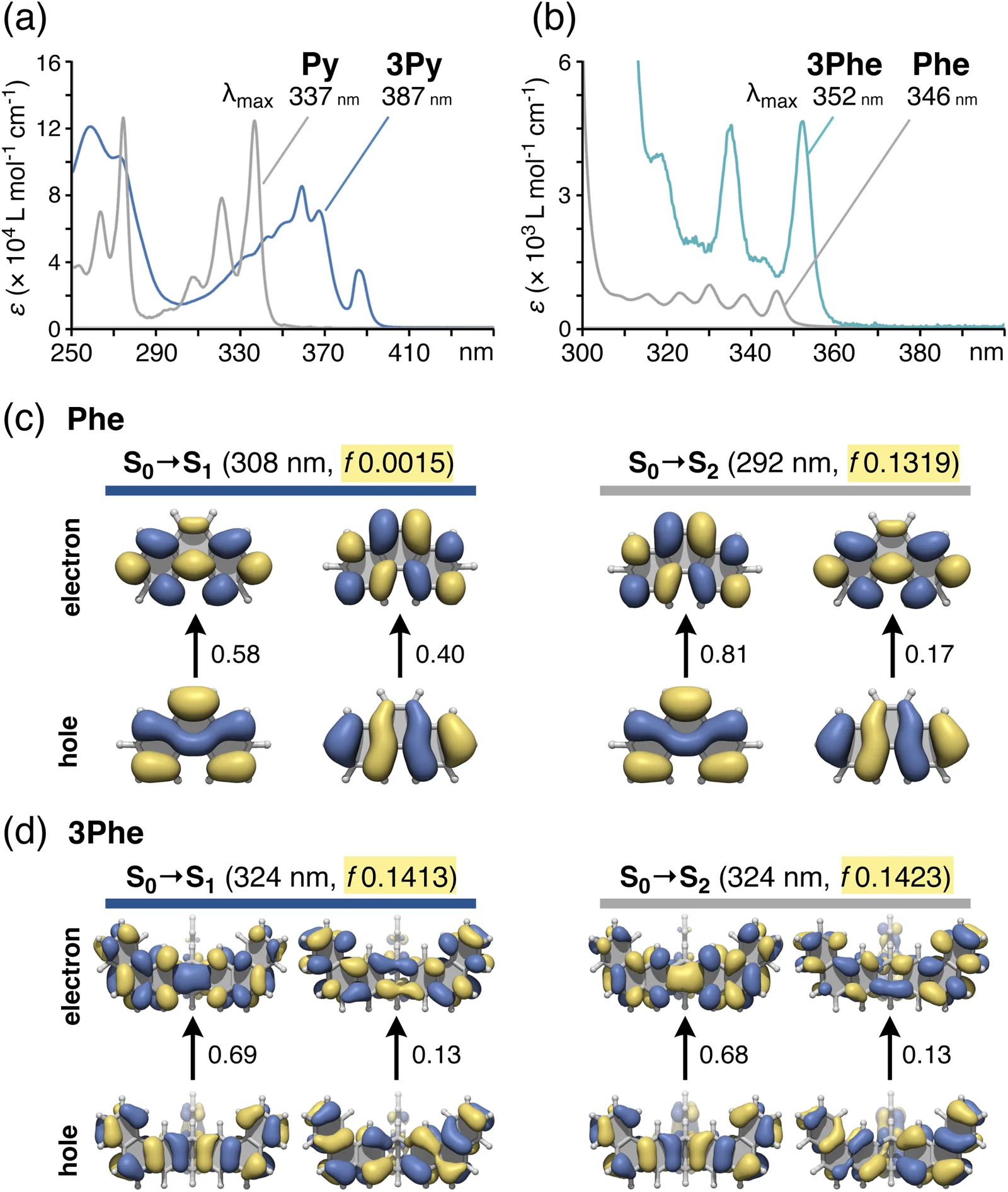
Absorption properties of π-extended triptycenes. UV-vis spectra of (a) Py and 3Py in the 250–450 nm region (CHCl_3_, 1.0 × 10^−5^ M) and (b) Phe and 3Phe in the 300–400 nm region (CHCl_3_, Phe: 1.0 × 10^−3^ M to observe the forbidden transition, 3Phe: 1.0 × 10^−5^ M). The molar absorption coefficients (*ε*) of Py and Phe were multiplied by a factor of three. Optimized structures and corresponding NTOs for the S_1_ and S_2_ states of (c) Phe and (d) 3Phe, calculated at the B3LYP-D3(BJ)/6-31G(d,p) level (PCM, CHCl_3_).

**Table 1:** Optical data of the compounds in CHCl_3_

Compound	*λ*_abs_/nm	*ε* a/L mol^−1^ cm^−1^	*λ*_em_/nm	*Φ*_F_	*τ*/ns	*k*_r_/s^−1^	*k*_nr_/s^−1^
Py	337	1.24 × 10^5^	375	0.12	49	2.5 × 10^6^	18 × 10^6^
3Py	387	3.42 × 10^4^	393	0.57	7.5	77 × 10^6^	58 × 10^6^
Phe	346	8.16 × 10^2^	350	0.06	16	3.8 × 10^6^	59 × 10^6^
3Phe	352	4.63 × 10^3^	356	0.14	11	13 × 10^6^	79 × 10^6^

a The values of Py and Phe were multiplied by a factor of three. The original values were 4.14 × 10^4^ (Py) and 2.72 × 10^2^ (Phe).

In the fluorescence spectra, both 3Py and 3Phe exhibited bathochromic emission relative to Py and Phe, respectively (Fig. 6a and b, and Tables 1 and S8). Their fluorescence quantum yields (*Φ*_F_) increased by *ca.* 2–5-fold compared with the parent PAH, respectively. Interestingly, the fluorescence lifetimes (*τ*) of both triptycene derivatives were shorter than those of their corresponding subunits. To elucidate the origin of the enhanced fluorescence, the radiative (*k*_r_) and nonradiative (*k*_nr_) decay rate constants were calculated. As a result, *k*_nr_ of 3Py and 3Phe increased by only *ca.* 1–3-fold, whereas *k*_r_ increased much more substantially by *ca.* 3–30-fold relative to those of Py and Phe. These results indicate that the enhanced fluorescence originates primarily from an increase in the radiative decay rate.^24,26^

**Fig. 6:**
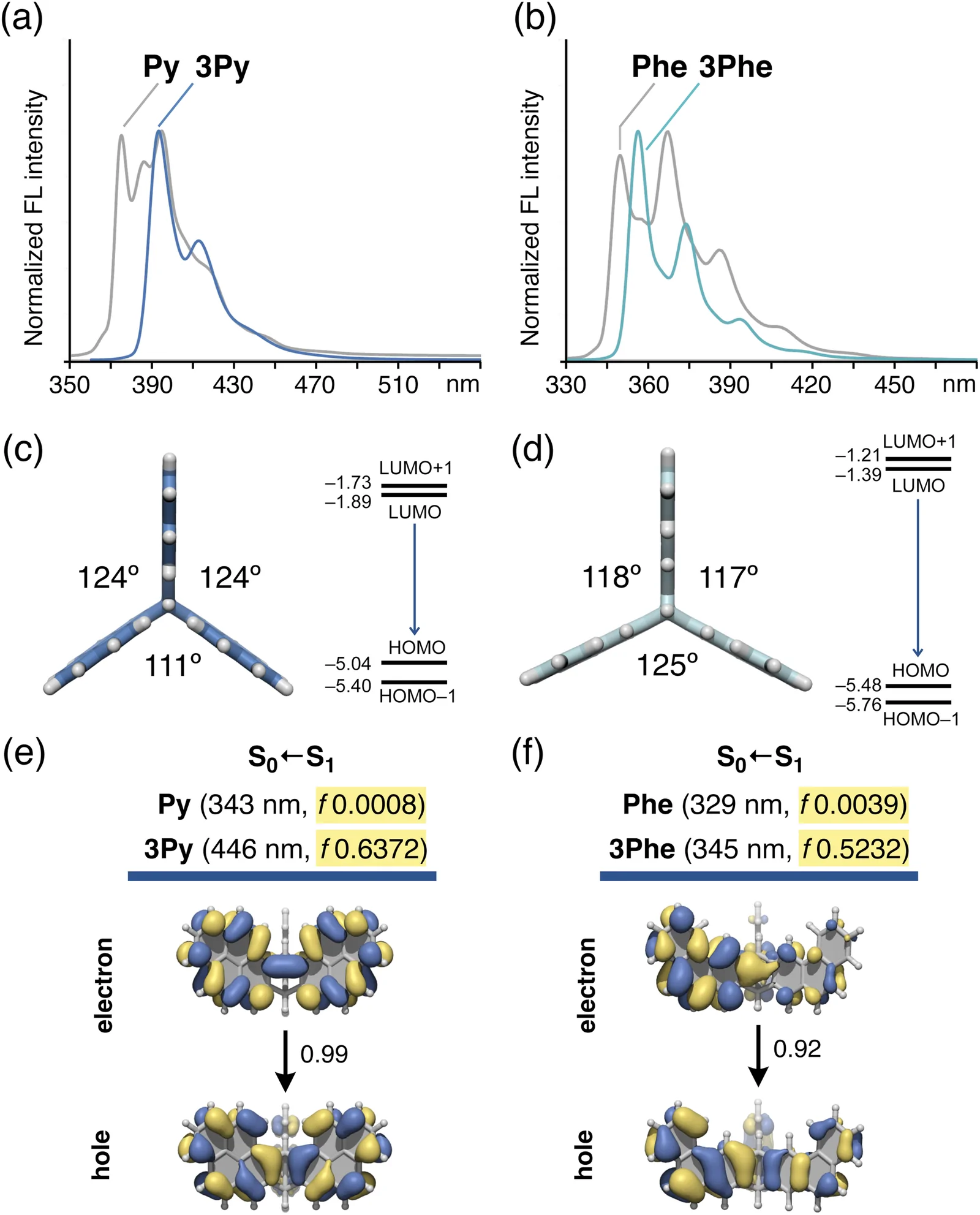
Fluorescence properties of π-extended triptycenes. Fluorescence spectra of (a) Py and 3Py and (b) Phe and 3Phe (CHCl_3_, 1.0 × 10^−5^ M). The optimized S_1_ structures and corresponding energy diagrams of (c) 3Py and (d) 3Phe. NTOs for the S_0_←S_1_ transitions of (e) Py and 3Py and (f) Phe and 3Phe, together with the emission wavelengths and oscillator strengths (*f*) of the S_1_ states. DFT and TD-DFT calculations were performed at the B3LYP-D3(BJ)/6-31G(d,p) (PCM, CHCl_3_) level.

This fluorescence behavior is consistent with the TD-DFT calculations using the excited-state optimized geometries. Unlike the ground-state structure, the optimized excited-state geometries of 3Py and 3Phe exhibited structural distortions (Fig. 6c, d, and S14). The calculated emissions reproduced the experimentally observed trend in the bathochromic shifts, reflecting stabilization of the lowest excited states through homoconjugation (Fig. S15). For both Py and Phe, the S_1_ states were dominated by the HOMO←LUMO+1 (*ca.* 60%) and HOMO–1←LUMO (*ca.* 40%) transitions, resulting in negligible oscillator strength (*f* 0.001–0.004) and an essentially forbidden S_0_←S_1_ transition (Table S9). In contrast, the S_1_ states of 3Phe and 3Py were dominated by the HOMO←LUMO (3Py*ca.* 99%, 3Phe*ca.* 92%) transition, giving rise to large oscillator strengths (*f* 0.6372 for 3Py and 0.5232 for 3Phe) and allowed S_0_←S_1_ transitions. These trends were also observed using CAM-B3LYP-D3(BJ) and PBE0 (Table S10 and Fig. S16). NTO analyses further revealed a pronounced change in the S_1_-state character: the S_1_ states of Py and Phe involved two NTO pairs, whereas those of 3Py and 3Phe were predominantly described by a single NTO pair (Fig. 6e and f). These results indicate that homoconjugative interactions modify the S_1_ electronic states and increase the allowed character of S_0_←S_1_ transitions, thereby accounting for the substantially enhanced radiative decay rates of 3Phe and 3Py. In contrast to CHCl_3_ solution, both 3Py and 3Phe exhibited weak fluorescence in the solid state (Fig. S13).

Finally, the electrochemical properties of 3Py and 3Phe were investigated by cyclic voltammetry (CV) and differential pulse voltammetry (DPV). All compounds (3Py, 3Phe, Py, and Phe) exhibited irreversible oxidation waves in the cyclic voltammograms,^54^ preventing accurate determination of the oxidation potentials from the CV data (Fig. S17). Therefore, differential pulse voltammetry (DPV) was employed to obtain the oxidation potentials more reliably (Fig. S18). For 3Phe, the first oxidation potential was 0.06 V (*vs.* Fc/Fc^+^) lower than that of Phe, consistent with the destabilization of the HOMO estimated by DFT calculations. In contrast, the oxidation potential of 3Py was slightly higher than that of Py (Δ*V* = 0.02 V), despite the predicted HOMO destabilization. Given the irreversible oxidation and broad DPV responses, particularly for 3Py, the oxidation behavior cannot be interpreted solely in terms of the calculated HOMO energy. The observed potentials may also be influenced by processes accompanying oxidation, including reactions or intermolecular interactions on the electrode surface.

## Conclusions

In summary, we have synthesized pyrene- and phenanthrene-embedded triptycene derivatives and investigated the effects of homoconjugation on their optical and electrochemical properties. Selective synthesis of π-extended triptycenes was achieved through Negishi cross-coupling followed by Pt-catalyzed cyclization. Structural characterization revealed that the pyrene-embedded triptycene derivative unexpectedly forms dual-pore crystals capable of clathrating perfluorobenzene. Homoconjugation induced red-shifted absorption and emission together with enhanced fluorescence quantum yields in both triptycene derivatives, which is in good agreement with the DFT and TD-DFT calculations. These findings provide valuable design guidelines for the development of three-dimensional π-conjugated molecules with enhanced fluorescence through homoconjugative interactions.

## Author contributions

Conceptualization: M. Y. and S. T.; methodology: P. H., Y. W., M. Y. and S. T.; investigation: P. H. and Y. W.; formal analysis: P. H., Y. W. and M. Y.; visualization: P. H. and M. Y.; writing – original draft: P. H. and M. Y.; writing – review & editing: M. Y. and S. T.; supervision: M. Y. and S. T.; project administration: M. Y. and S. T.; funding acquisition: M. Y. and S. T.

## Conflicts of interest

There are no conflicts to declare.

## Supplementary Material

SC-OLF-D6SC07032A-s001

## Data Availability

CCDC 2535452 (3Py·CHCl_3_), 2535453 (3Py·C_6_F_6_), 2535454 (3Phe) and 2535455 (T3Phe) (CIF) contain the supplementary crystallographic data for this paper.^55*a–d*^ The data underlying this study are available in the published article and its supplementary information (SI). Supplementary information is available. See DOI: https://doi.org/10.1039/d6sc07032a.
