# Supplementary material for: Homoconjugation-induced fluorescence enhancement in C3v-symmetric pyrene- and phenanthrene-embedded triptycenes
Source: Chem Sci. 2026 Nov 10. Online ahead of print. doi: 10.1039/d6sc07032a (PMC13760498; doi:10.1039/d6sc07032a)
Supplement: Supplementary file 1 [file SC-OLF-D6SC07032A-s001.pdf]

Supplementary Information (SI) for Chemical Science.
This journal is © The Royal Society of Chemistry

Homoconjugation-Induced Fluorescence Enhancement in C_{3v}-Symmetric Pyrene- and Phenanthrene-Embedded Triptycenes

Puyi Huang, Yu Watanabe, Masahiro Yamashina*, and Shinji Toyota*

Department of Chemistry, School of Science, Institute of Science Tokyo, 2-12-1 Ookayama, Meguro-ku, Tokyo 152-8551, Japan.

Table of Contents

1. General S3

2. Synthesis S6

2.1. Synthesis of 2-bromo-1,3-bis[2-(trimethylsilyl)ethynyl]benzene (**A**) S6

2.2. Synthesis of **1a**–**3a** S7

2.3. Synthesis of **1b** and **2b** S8

2.4. Synthesis of **3Py** S10

2.5. Pt-Catalyzed cycloisomerization of **2b** S11

2.6. Synthesis of **3b** S12

2.7. Synthesis of **T3Phe** S13

2.8. Synthesis of **3Phe** S14

3. Single crystal X-ray diffraction measurements S15

4. DFT calculations S19

4.1. Ground-state optimized structures S19

4.2. NMR analyses S20

4.3. TD-DFT calculations S22

5. Optical properties S27

5.1. UV–vis and fluorescence properties S27

5.2. Excited-state optimized structures S31

6. Electrochemical measurements S34

7. NMR spectra S35

8. Cartesian coordinates S52

1. General

Materials and methods

All reagents and solvents were purchased from commercial sources and used as received. NMR spectra were recorded using a JEOL 500 MHz JNM-ECX500 spectrometer. Chemical shifts for ^{1}H and ^{13}C are reported in ppm on the δ scale; ^{1}H signals were calibrated with TMS (0.00 ppm) as an internal standard. ^{13}C signals were calibrated with the solvent signals of $CDCl_3$ (77.16 ppm). Matrix-Assisted Laser Desorption/Ionization High-resolution mass spectra (MALDI-HRMS) were measured on a Bruker UltrafleXtreme mass spectrometer using DCTB (*trans*-2-[3-(4-*t*-butylphenyl)-2-methyl-2-propenylidene]malononitrile) as a matrix and PEG (polyethylene glycol) as a calibrant. X-ray crystallography was carried out with a diffractometer equipped in a beamline BL-5A at the High Energy Accelerator Research Organization (KEK), Japan with a Pilatus3 S6M detector (synchrotron, λ = 0.7500 Å) and processed using XDS ver 2022.01.10[S1]. The structure was solved by intrinsic phasing methods using SHELXT-2018[S2] and then refined with SHELXL-2016[S3] using the Olex2 1.5[S4] as graphical user interfaces. All non-hydrogen atoms were refined with anisotropic displacement parameters. All hydrogen atoms were created with ideal geometry and refined using a riding model. UV-visible absorption and fluorescence spectra were recorded using a JASCO V-730 spectrometer and a JASCO FP-8550 spectrofluorometer. Samples were analyzed at room temperature using quartz cuvettes with optical path lengths of 10.0 mm. Absolute fluorescence quantum yield was measured using a JASCO FP-8550 with an ILF-135 attachment. Fluorescence lifetime was measured using a Hamamatsu Quantaurus-Tau C11367-02. Melting points were measured using a Yanaco MP-500P micro melting point apparatus without standard correction and data are uncorrected. Recycling gel permeation chromatography (GPC) was performed on a JAI LaboACE LC-5060 recycling preparative HPLC system equipped with an RI-700LA refractive index detector and a JAIGEL-2.5HR column. Cyclic voltammograms and differential pulse voltammograms were recorded using an EC-Frontier ECstat-302. The data were collected in Benzene/Acetonitrile (1/1) solution with 0.10 mol L^{-1} of Bu_4NPF_6 as electrolyte, using a Pt disk electrode as the working electrode, a Pt wire as the counter electrode, and an Ag/AgCl in a Luggin capillary as the reference electrode. The scan rate was 100 mV s^{-1}. All potentials were referenced to Fc/Fc^{+} under identical conditions. All DFT calculations were performed using the Gaussian 16 program suite[S5] and NTO analyses were conducted using Multiwfn[S6].

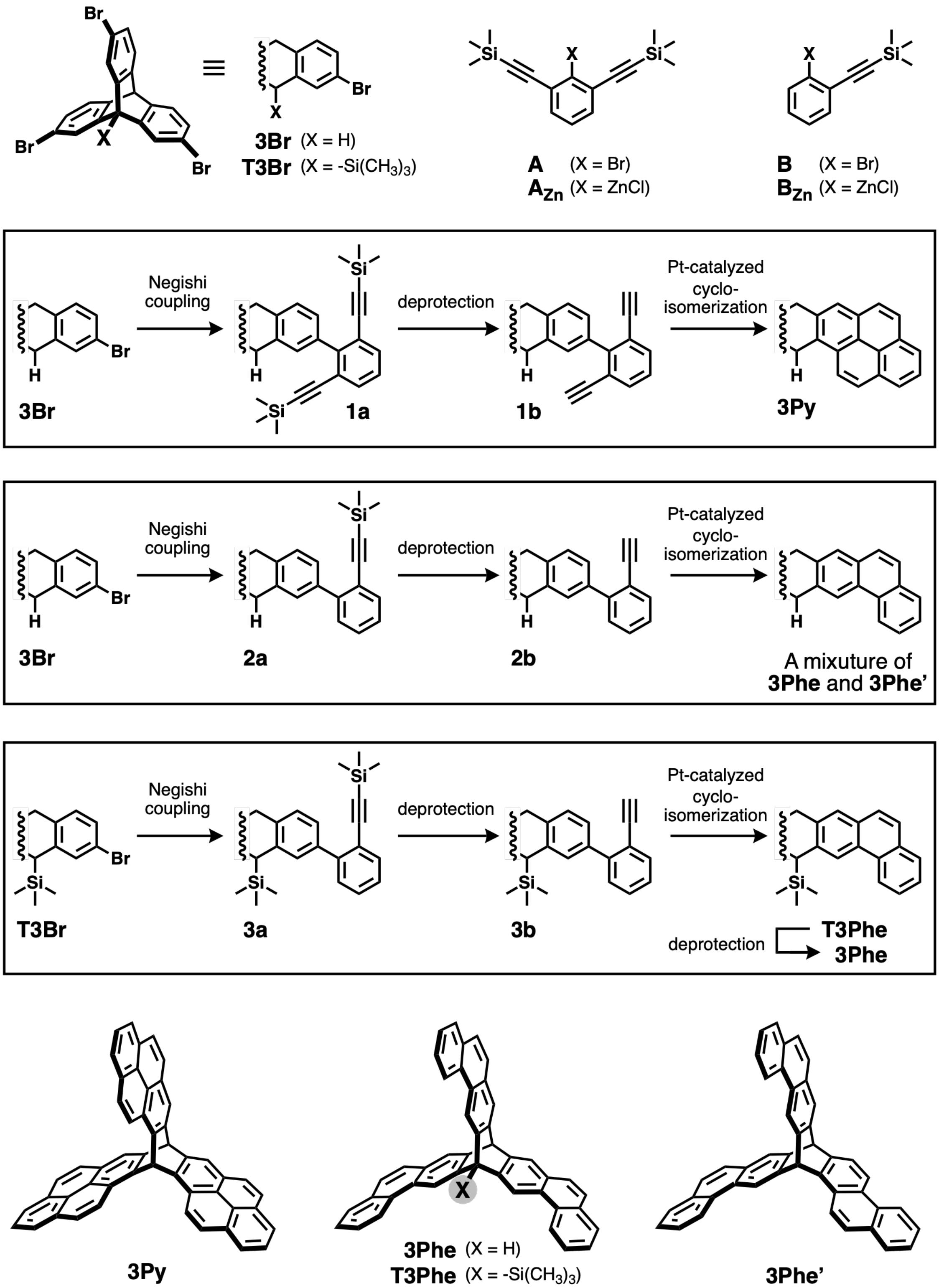

Figure S1. Synthetic routes of **3Phe**, **T3Phe**, **3Phe'**, and **3Py**.

References

S1. W. Kabsch, *Acta Crystallogr., Sect. D: Biol. Crystallogr.*, 2010, **66**, 133–144.

S2. G. M. Sheldrick, *Acta Crystallogr., Sect. A: Found. Adv.*, 2015, **71**, 3–8.

S3. G. M. Sheldrick, *Acta Crystallogr., Sect. C: Struct. Chem.*, 2015, **71**, 3–8.

S4. O. V. Dolomanov, L. J. Bourhis, R. J. Gildea, J. A. K. Howard and H. Puschmann, *J. Appl. Crystallogr.*, 2009, **42**, 339–341.

S5. M. J. Frisch, G. W. Trucks, H. B. Schlegel, G. E. Scuseria, M. A. Robb, J. R. Cheeseman, G. Scalmani, V. Barone, G. A. Petersson, H. Nakatsuji, X. Li, M. Caricato, A. V. Marenich, J. Bloino, B. G. Janesko, R. Gomperts, B. Mennucci, H. P. Hratchian, J. V. Ortiz, A. F. Izmaylov, J. L. Sonnenberg, D. Williams-Young, F. Ding, F. Lipparini, F. Egidi, J. Goings, B. Peng, A. Petrone, T. Henderson, D. Ranasinghe, V. G. Zakrzewski, J. Gao, N. Rega, G. Zheng, W. Liang, M. Hada, M. Ehara, K. Toyota, R. Fukuda, J. Hasegawa, M. Ishida, T. Nakajima, Y. Honda, O. Kitao, H. Nakai, T. Vreven, K. Throssell, J. A. Montgomery, Jr., J. E. Peralta, F. Ogliaro, M. J. Bearpark, J. J. Heyd, E. N. Brothers, K. N. Kudin, V. N. Staroverov, T. A. Keith, R. Kobayashi, J. Normand, K. Raghavachari, A. P. Rendell, J. C. Burant, S. S. Iyengar, J. Tomasi, M. Cossi, J. M. Millam, M. Klene, C. Adamo, R. Cammi, J. W. Ochterski, R. L. Martin, K. Morokuma, O. Farkas, J. B. Foresman and D. J. Fox, *Gaussian 16, Revision C.01*, Gaussian, Inc., Wallingford, CT, USA, 2016.

S6. T. Lu and F. Chen, *J. Comput. Chem.*, 2012, **33**, 580–592.

S7. H. Ito, T. Abe and K. Saigo, *Angew. Chem. Int. Ed.*, 2011, **50**, 7144–7147.

S8. K. Ueno, Y. Nishii and M. Miura, *Org. Lett.*, 2021, **23**, 3552–3556.

S9. T. Matsuda, K. Kato, T. Goya, S. Shimada and M. Murakami, *Chem. Eur. J.* 2016, **22**, 1941.

S10. J. M. Granda, J. Grabowski and J. Jurczak, *Org. Lett.* 2015, **17**, 5882–5885.

S11. E. Posenitskiy, F. Spiegelman and D. Lemoine, *Mach. Learn.: Sci. Technol.*, 2021, **2**, 035039.

2. Synthesis

2.1. Synthesis of 2-bromo-1,3-bis[2-(trimethylsilyl)ethynyl]benzene (A) PH-1-171

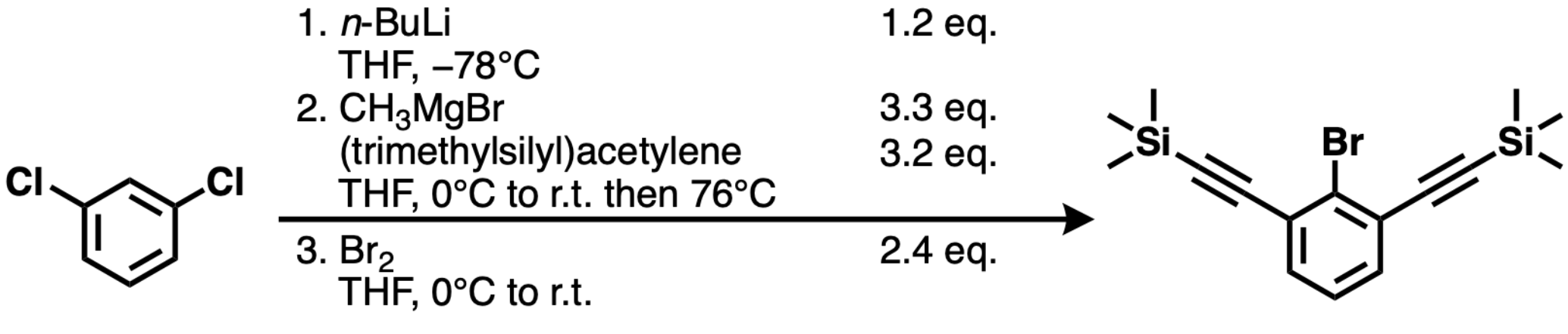

50% A

The synthesis was carried out by modifying the reported procedure[S7].

A 300 mL two-neck flask (Flask A) was evacuated and backfilled with argon three times. Anhydrous tetrahydrofuran (THF; 20 mL) was added, followed by the addition of (trimethylsilyl)acetylene (3.92 g, 39.9 mmol). Methylmagnesium bromide (CH_3MgBr; 3.0 M in diethyl ether, 13.5 mL, 40.5 mmol) was then added dropwise to the solution under stirring at room temperature. The reaction mixture was stirred for at least 2 h, during which a white precipitate formed, indicating the formation of [2-(trimethylsilyl)ethynyl]magnesium bromide.

In a 100 mL two-necked flask (Flask B), purged with argon in the same manner, 1,3-dichlorobenzene (1.83 g, 12.5 mmol) was dissolved in anhydrous THF (50 mL) and cooled to –78°C. Under stirring, *n*-butyllithium (*n*-BuLi; 6.5 mL, 2.3 M in hexane, 15.0 mmol) was slowly added dropwise. The solution was further stirred at –78°C for 3 h, after which it was rapidly transferred via a cannula into Flask A (precooled to 0°C). The obtained pale yellow solution was stirred at room temperature for 20 h, affording a dark-colored solution. The mixture was then refluxed for 3 h and subsequently cooled to 0°C. Bromine (1.5 mL, 29.3 mmol) was slowly added dropwise to the flask, and the solution was stirred at room temperature for 3 h.

After the reaction was completed, sat. $Na_2S_2O_3$ aq. (50 mL) was added, and the organic solvent was removed under reduced pressure using a rotary evaporator. The residue was dissolved in AcOEt, and washed with sat. $NaHCO_3$ aq. The organic phase was dried over $MgSO_4$, filtered, and concentrated under reduced pressure. The crude product was purified by column chromatography on silica gel (hexane) and recrystallization (MeOH/DCM) to give colorless crystals (2.18 g, 50% yield).

^{1}H NMR (500 MHz, $CDCl_3$, 298 K): δ 7.41 (d, J = 7.7 Hz, 2H), 7.17 (t, J = 7.7 Hz, 1H), 0.27 (s, 18H); ^{13}C NMR (125 MHz, $CDCl_3$, 298 K): δ 133.3 (CH), 128.9 (C_q), 126.6 (CH),

126.2 (C_q), 103.0 (-*C*≡C-Si), 100.1 (-C≡*C*-Si), –0.1 ($SiCH_3$); m.p. = 110.0–112.1°C. R_f = 0.38 (hexane).

2.2. Synthesis of 1a–3a

PH-2-079, PH-2-011, PH-2-065

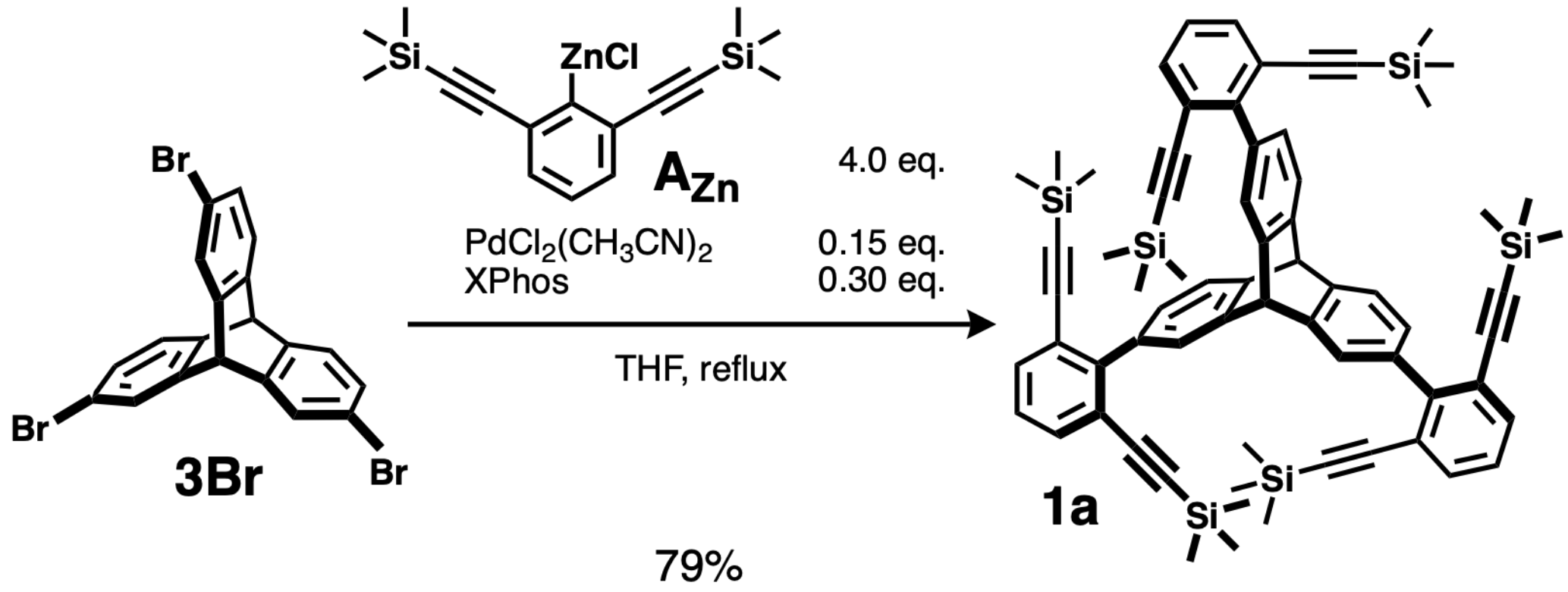

Compound **A** (1.69 g, 4.84 mmol) and anhydrous THF (8.0 mL) were added to an argon-purged 20 mL two-neck flask (Flask A), and the solution was cooled to –78°C. *n*-Butyllithium (2.1 mL, 2.3 M in hexane, 4.83 mmol) was slowly added via syringe under stirring. The reaction mixture was stirred for 1 h while gradually warming from –78°C to –40°C. Subsequently, an anhydrous THF solution (20.0 mL) of anhydrous zinc chloride ($ZnCl_2$; 1.25 g, 9.17 mmol) was added dropwise, and the mixture was stirred for 1 h while warming from –40°C to room temperature.

To an argon-purged 100 mL two-neck flask (Flask B), compound **3Br**[S8] (0.594 g, 1.21 mmol), $PdCl_2(CH_3CN)_2$ (18.1 mg, 69.7 μmol), XPhos (69.7 mg, 0.146 mmol), and anhydrous THF (10.0 mL) were added. The mixture was stirred at room temperature, and the solution of $\mathbf{A_{Zn}}$ in Flask A was transferred to Flask B via cannula. The resultant mixture was refluxed for 48 h. After the reaction was completed, H_2O (5.0 mL) was added, and the organic solvent was removed under reduced pressure using a rotary evaporator. The residue was dissolved in AcOEt, and washed with sat. $NaHCO_3$ aq. The organic phase was dried with $MgSO_4$, filtered, and concentrated under reduced pressure. The crude product was purified by column chromatography on silica gel (hexane/DCM = 4:1) to give a yellow solid (1.01 g, 79% yield).

Using the same procedure, **2a** and **3a** were synthesized as follows. **2a** (82% yield, a white solid) was synthesized from **3Br** (0.121 g, 0.246 mmol) with **B** (0.372 g, 1.47

mmol), $PdCl_2(CH_3CN)_2$ (10.2 mg, 39.3 μmol), and SPhos (30.2 mg, 73.6 μmol). **3a** (88% yield, a pale yellow solid) was obtained from **T3Br**[S8] (0.55 g, 0.98 mmol) with 1-bromo-2-(trimethylsilylethynyl)benzene **B**[S9] (1.73 g, 6.84 mmol), $PdCl_2(CH_3CN)_2$ (25.3 mg, 97.5 μmol), and SPhos (80.2 mg, 0.195 mmol).

for **1a**

^{1}H NMR (500 MHz, $CDCl_3$, 298 K): δ 7.57 (s, 3H), 7.46 (d, J = 7.7 Hz, 6H), 7.39 (d, J = 7.4 Hz, 3H), 7.23 (d, J = 7.4 Hz, 3H), 7.14 (t, J = 7.7 Hz, 3H), 5.51 (s, 1H), 5.47 (s, 1H), −0.14 (s, 54H); ^{13}C NMR (125 MHz, $CDCl_3$, 298 K): δ 146.9 (C_q), 145.1 (C_q), 144.2 (C_q), 134.9 (CH), 133.3 (CH), 128.0 (CH), 126.6 (CH), 126.2 (CH), 123.2 (C_q), 122.4 (C_q), 104.7 (-<u>*C*</u>≡C-Si), 98.6 (-C≡<u>*C*</u>-Si), 54.5 (CH), 54.2 (CH), 0.0 ($SiCH_3$); HRMS (MALDI-TOF) *m*/*z*: $[M]^+$ calcd for $C_{68}H_{74}Si_6$, 1058.4401, PEG1000, DCTB; found 1058.4400; m.p. = 230.3–231.5°C. R_f = 0.43 (hexane/CH_2Cl_2 = 4:1).

for **2a**

^{1}H NMR (500 MHz, $CDCl_3$, 298 K): δ 7.66 (s, 3H), 7.52 (d, J = 7.7 Hz, 3H), 7.45 (d, J = 7.4 Hz, 3H), 7.31–7.27 (m, 9H), 7.24–7.20 (m, 3H), 5.55 (s, 1H), 5.52 (s, 1H), 0.00 (s, 27H); ^{13}C NMR (125 MHz, $CDCl_3$, 298 K): δ 144.9 (C_q), 144.5 (C_q), 144.0 (C_q), 137.4 (C_q), 133.6 (CH), 129.5 (CH), 128.7 (C_q), 126.7 (CH), 126.4 (CH), 124.9 (CH), 123.1 (CH), 121. 4 (CH), 105.0 (-<u>*C*</u>≡C-Si), 97.8 (-C≡<u>*C*</u>-Si), 54.5 (CH), 53.6 (CH), –0.2 ($SiCH_3$); HRMS (MALDI-TOF) *m*/*z*: $[M]^+$ calcd for $C_{53}H_{50}Si_3$, 770.3215; found 770.3195, PEG1000, DCTB; m.p. = 221.1–223.1°C. R_f = 0.44 (hexane/CH_2Cl_2 = 4:1).

for **3a**

^{1}H NMR (500 MHz, $CDCl_3$, 298 K): δ 7.57–7.50 (m, 6H), 7.45 (d, J = 7.7 Hz, 3H), 7.38 (d, J = 8.9 Hz, 3H), 7.33 (t, J = 7.0 Hz, 3H), 7.27–7.21 (m, 6H), 5.47 (s, 1H), 0.79 (s, 9H), –0.05 (s, 27H); ^{13}C NMR (125 MHz, $CDCl_3$, 298 K): δ 147.6 (C_q), 146.3 (C_q), 144.7 (C_q), 136.8 (C_q), 133.6 (CH), 129.4 (CH), 128.7 (CH), 126.7 (CH), 126.4 (CH), 125.9 (CH), 123.1 (CH), 121.8 (C_q), 105.0 (-<u>*C*</u>≡C-Si), 97.8 (-C≡<u>*C*</u>-Si), 54.5 (CH), 47.8 (C_q), 2.4 ($SiCH_3$), –0.3 ($SiCH_3$); HRMS (MALDI-TOF) *m*/*z*: $[M]^+$ calcd for $C_{56}H_{58}Si_4$, 842.3610; found 842.3596, PEG1000, DCTB; m.p. = 217.8–218.8°C. R_f = 0.47 (hexane/CH_2Cl_2 = 4:1).

2.3. Synthesis of 1b and 2b

PH-2-003, PH-2-019

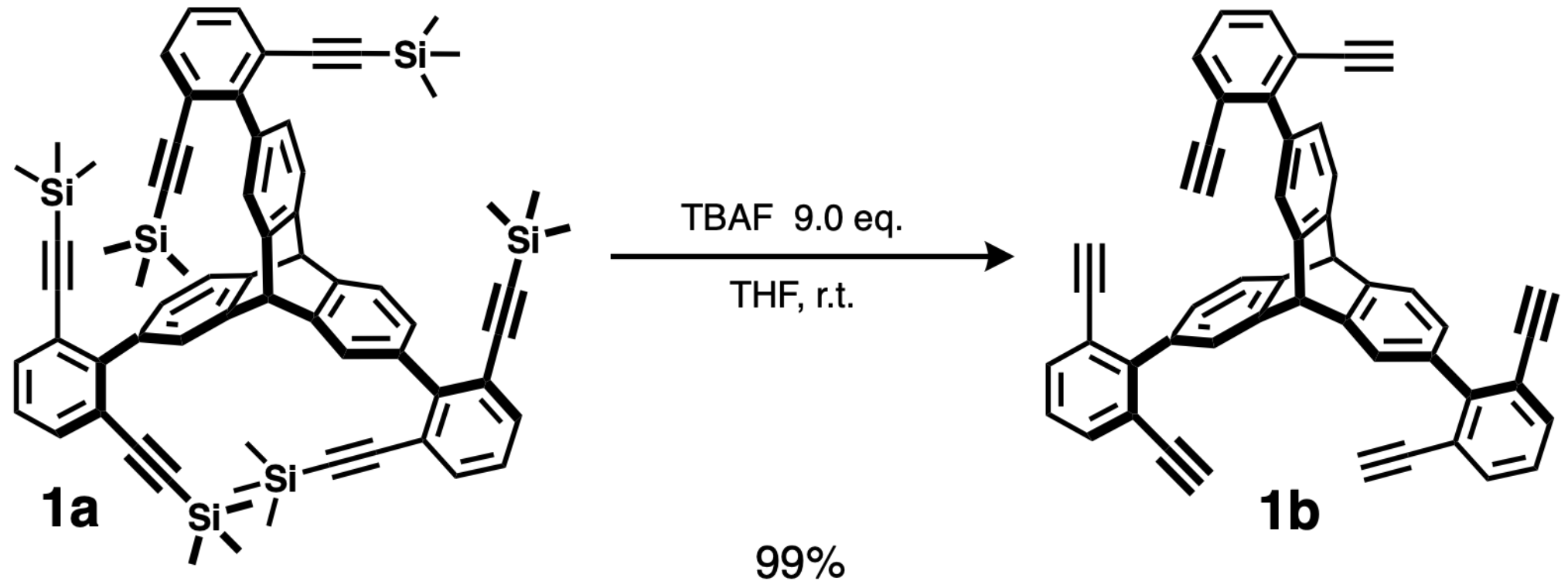

Compound **1a** (0.115 g, 0.11 mmol) and anhydrous THF (30 mL) were added to a 50 mL round-bottom flask. Tetrabutylammonium fluoride (TBAF; 1.0 M in THF, 0.98 mL, 0.98 mmol) was added dropwise, and the resultant mixture was stirred at room temperature for 16 h. After the reaction was completed, the solvent was removed under reduced pressure using a rotary evaporator. The crude product was purified by column chromatography on silica gel (hexane/DCM = 2:1) to give a white solid (67.3 mg, 99% yield).

Using the same procedure, **2b** (96% yield, a white solid) was synthesized from **2a** (0.118 g, 0.153 mmol).

for **1b**

^{1}H NMR (500 MHz, $CDCl_3$, 298 K): δ 7.54 (d, J = 7.2 Hz, 6H), 7.52 (s, 3H), 7.48 (d, J = 7.4 Hz, 3H), 7.23 (t, J = 7.9 Hz, 3H), 7.14 (d, J = 7.7 Hz, 3H), 5.57 (s, 1H), 5.51 (s, 1H), 2.81 (s, 6H); ^{13}C NMR (125 MHz, $CDCl_3$, 298 K): δ 147.3 (C_q), 144.8 (C_q), 135.1 (C_q), 133.8 (CH), 126.9 (CH), 126.5 (CH), 125.5 (CH), 123.2 (CH), 122.3 (C_q), 82.4 (-$\underline{C}$≡CH), 80.9 (-C≡$\underline{C}$H), 54.2 (CH), 53.8 (CH). One aromatic CH signal is overlapped; HRMS (MALDI-TOF) m/z: $[M]^+$ calcd for $C_{50}H_{26}$, 626.2029; found 626.2027, PEG 400, DCTB; m.p. = 196°C (decomp.). R_f = 0.10 (hexane/CH_2Cl_2 = 4:1).

for **2b**

^{1}H NMR (500 MHz, $CDCl_3$, 298 K): δ 7.65 (s, 3H), 7.58 (d, J = 7.4 Hz, 3H), 7.48 (d, J = 7.4 Hz, 3H), 7.39–7.29 (m, 9H), 7.29–7.23 (m, 3H), 5.58–5.54 (m, 2H), 2.99 (s, 3H); ^{13}C

NMR (125 MHz, $CDCl_3$, 298 K): δ 145.2 (C_q), 144.5 (C_q), 144.2 (C_q), 137.3 (C_q), 134.1 (CH), 129.9 (CH), 129.1 (CH), 126.9 (CH), 126.4 (CH), 124.8 (CH), 123.4 (CH), 120.4 (C_q), 83.4 (-$\underline{C}$≡CH), 80.4 (-C≡$\underline{C}$H), 54.4 (CH), 53.5 (CH); HRMS (MALDI-TOF) *m*/*z*: $[M]^+$ calcd for $C_{44}H_{26}$, 554.2029; found 554.2041, PEG 400, DCTB; m.p. = 170°C (decomp.). R_f = 0.07 (hexane/CH_2Cl_2 = 4:1).

2.4. Synthesis of 3Py

PH-2-015

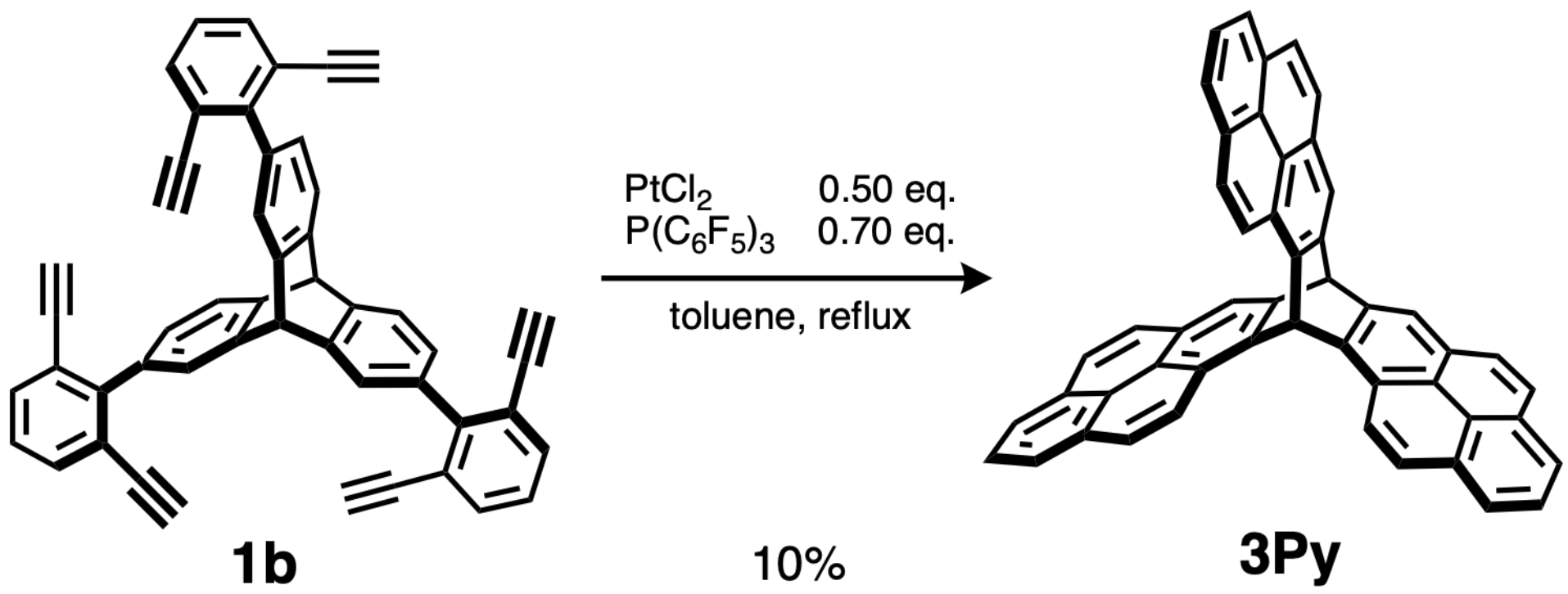

Compound **1b** (34.8 mg, 55.5 μmol), $PtCl_2$ (7.4 mg, 27.8 μmol), and tris(pentafluorophenyl)phosphine ($P(C_6F_5)_3$; 20.7 mg, 38.9 μmol) were placed into a 100 mL two-neck flask under an argon atmosphere. Anhydrous toluene (45 mL) was then added, and the reaction mixture was stirred at 110°C for 72 h. After the reaction was completed, the organic solvent was removed under reduced pressure using a rotary evaporator. The crude product was purified by column chromatography on silica gel (hexane/DCM = 4:1) to give a yellow solid (9.9 mg). The yellow solid was purified by GPC using toluene as the eluent (10 mL/min), yielding a yellow solid (3.4 mg, 10% yield).

^{1}H NMR (500 MHz, $CDCl_3$, 298 K): δ 9.07 (d, J = 9.2 Hz, 3H), 8.68 (s, 1H), 8.50 (s, 3H), 8.26 (d, J = 9.2 Hz, 3H), 8.14 (d, J = 7.7 Hz, 3H), 8.09 (d, J = 7.4 Hz, 3H), 8.00 (ABq, J = 9.5 Hz, 6H), 7.91 (t, J = 7.4 Hz, 3H), 6.66 (s, 1H); ^{13}C NMR (125 MHz, $CDCl_3$, 298 K): δ 144.9 (C_q), 139.1 (C_q), 131.4 (C_q), 130.8 (C_q), 129.1 (CH), 128.5 (CH), 127.3 (C_q), 126.3 (C_q), 125.6 (CH), 125.4 (CH), 125.2 (C_q), 124.9 (CH), 123.1 (C_q), 122.1 (CH), 120.8 (CH), 56.7 (CH), 40.6 (CH). One aromatic CH signal is overlapped; HRMS (MALDI-TOF) *m*/*z*: $[M]^+$ calcd for $C_{50}H_{26}$, 626.2029; found 626.2030, PEG 400, DCTB; m.p. = 295 °C (decomp.) R_f = 0.23 (hexane/CH_2Cl_2 = 4:1).

2.5. Pt-Catalyzed cycloisomerization of 2b

PH-2-021

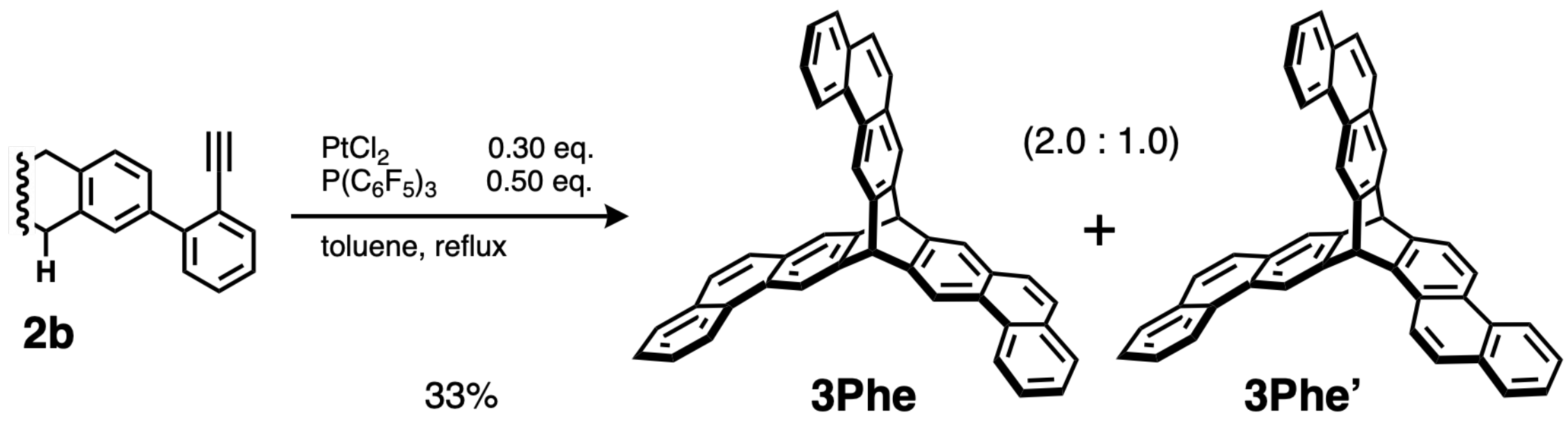

Compound **2b** (41.1 mg, 74.1 μmol), $PtCl_2$ (5.8 mg, 21.8 μmol), and $P(C_6F_5)_3$ (15.9 mg, 29.9 μmol) were placed into a 100 mL two-neck flask under an argon atmosphere. Anhydrous toluene (45 mL) was then added, and the reaction mixture was stirred at 116°C for 72 h. After the reaction was completed, the organic solvent was removed under reduced pressure using a rotary evaporator. The residue was dissolved in AcOEt, and washed with sat. NaCl aq. The organic phase was dried with $MgSO_4$, filtered, and concentrated under reduced pressure. The crude product was purified by silica gel column chromatography (hexane/DCM = 2:1) to give a yellow solid (33.8 mg). The yellow solid was purified by GPC using toluene as the eluent (10 mL min^{-1}) to afford a mixture of constitutional isomers (**3Phe**:**3Phe'** = 2.0:1.0) as a yellow solid (14.4 mg, 33% yield).

GPC chromatogram of a mixture of **3Phe** and **3Phe'** (toluene, 10 mL/min)

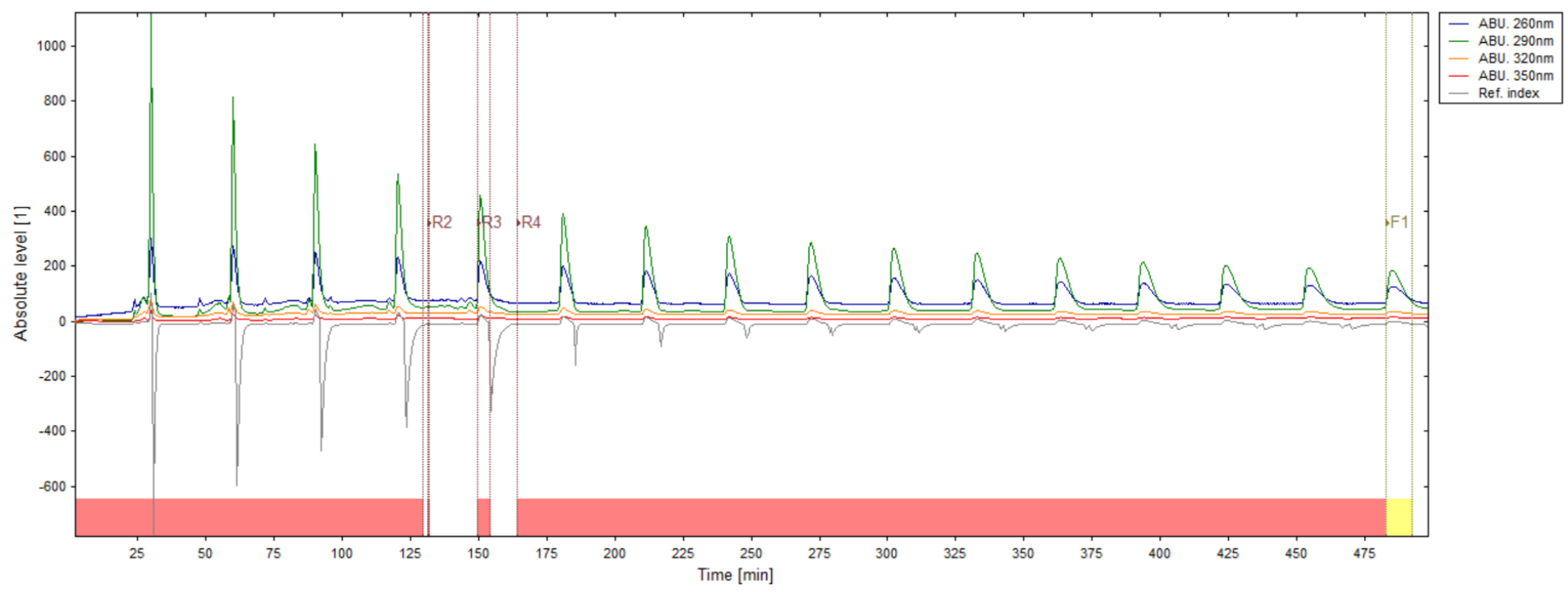

^{1}H NMR (500 MHz, $CDCl_3$, 298 K): δ 8.87 (s, **3Phe'**), 8.86 (s, **3Phe**), 8.72–8.67 (m, **3Phe**/**3Phe'**), 8.62–8.57 (m, **3Phe'**), 8.56 (d, J = 9.2 Hz, **3Phe'**), 8.48-8.45 (m, 1H, **3Phe'**), 8.01–7.97 (m, **3Phe**/**3Phe'**), 7.92-7.86 (m, **3Phe'**), 7.84–7.78 (m, **3Phe**/**3Phe'**), 7.71–7.46 (m, **3Phe**/**3Phe'**), 6.80 (s, **3Phe'**), 6.18 (s, **3Phe**), 6.00 (s, **3Phe'**), 5.97 (s, **3Phe**).

2.6. Synthesis of 3b

PH-2-077

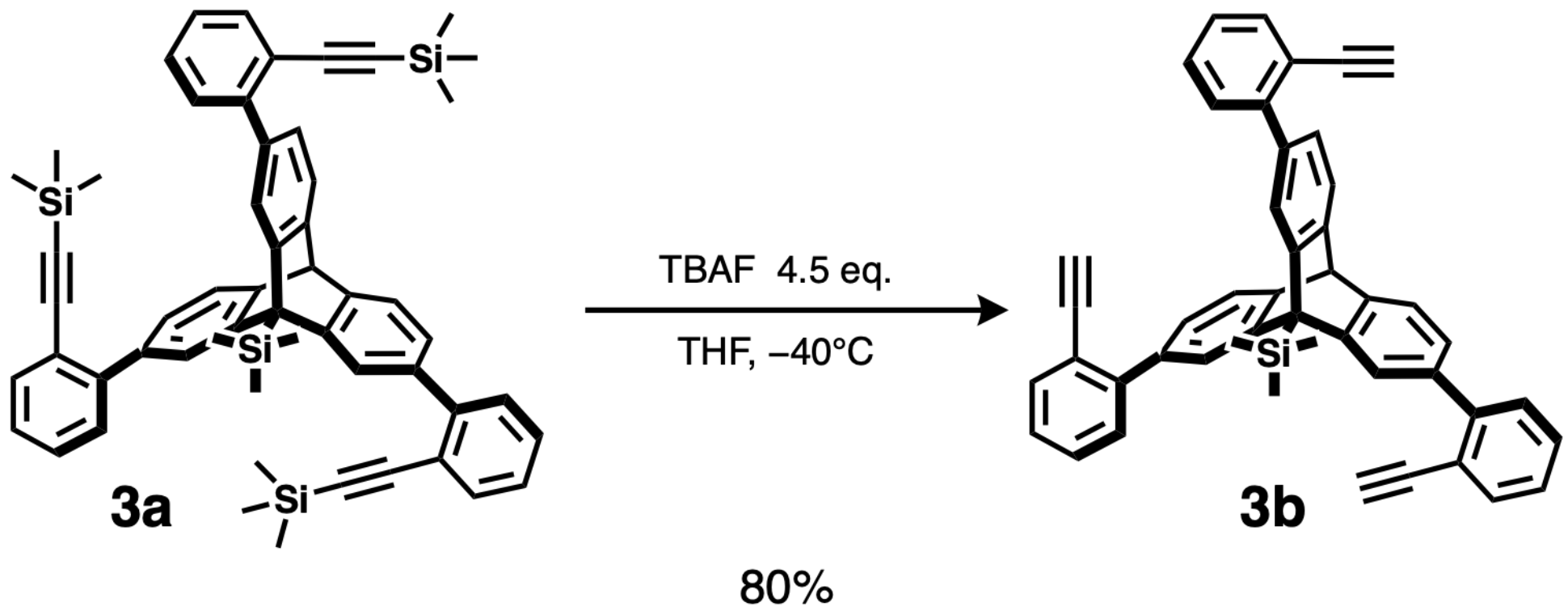

Compound **3a** (0.375 g, 0.44 mmol) and anhydrous THF (40 mL) were added to a 100 mL round-bottom flask, and the solution was cooled to –40°C. TBAF (1.0 M in THF, 1.4 mL, 1.4 mmol) was added dropwise, and the resultant mixture was stirred at room temperature for 2 h. After the reaction was completed, the solvent was removed under reduced pressure using a rotary evaporator. The crude product was purified by column chromatography on silica gel (hexane/DCM = 2:1) to give a white solid (0.223 g, 80% yield).

for **3b**

^{1}H NMR (500 MHz, $CDCl_3$, 298 K): δ 7.64 (s, 3H), 7.59 (d, J = 7.4 Hz, 3H), 7.49 (d, J = 7.4 Hz, 3H), 7.37 (t, J = 7.4 Hz, 3H), 7.31–7.23 (m, 9H), 5.48 (s, 1H), 2.97 (s, 3H), 0.79 (s, 9H); ^{13}C NMR (125 MHz, $CDCl_3$, 298 K): δ 147.6 (C_q), 146.4 (C_q), 144.7 (C_q), 136.8 (C_q), 134.2 (CH), 130.0 (CH), 129.1 (CH), 126.9 (CH), 126.5 (CH), 126.0 (CH), 123.8 (CH), 120.3 (C_q), 83.5 (-$\underline{C}$≡CH), 80.6 (-C≡$\underline{C}$H), 54.6 (CH), 48.0 (C_q), 2.3 ($SiCH_3$); HRMS (MALDI-TOF) *m*/*z*: $[M]^+$ calcd for $C_{47}H_{34}Si$, 626.2424; found 626.2442, PEG 400, DCTB; m.p. = 210°C (decomp.). R_f = 0.07 (hexane/CH_2Cl_2 = 4:1).

2.7. Synthesis of T3Phe

PH-2-057

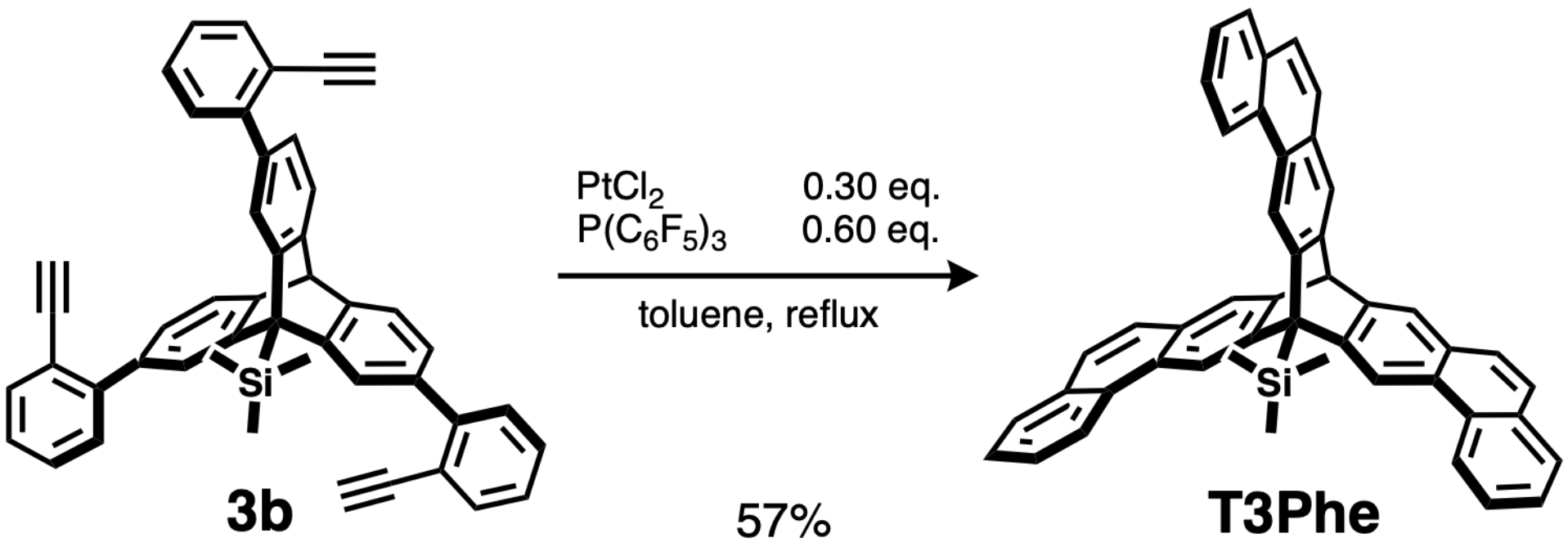

Compound **3b** (64.6 mg, 0.103 mmol), $PtCl_2$ (8.2 mg, 30.8 µmol), and $P(C_6F_5)_3$ (32.9 mg, 61.8 µmol) were placed into a 200 mL two-neck flask under an argon atmosphere. Anhydrous toluene (64 mL) was then added, and the reaction mixture was stirred at 110°C for 60 h. After the reaction was completed, the organic solvent was removed under reduced pressure using a rotary evaporator. The residue was dissolved in AcOEt, and washed with sat. NaCl aq. The organic phase was dried with $MgSO_4$, filtered, and concentrated under reduced pressure. The crude product was purified by column chromatography on silica gel (hexane/DCM = 2:1) to give a white solid (36.7 mg, 57% yield).

^{1}H NMR (500 MHz, $CDCl_3$, 298 K): δ 8.94 (s, 3H), 8.56 (d, J = 8.3 Hz, 3H), 8.00 (s, 3H), 7.82 (d, J = 7.8 Hz, 3H), 7.68–7.62 (m, 9H), 7.53 (t, J = 7.3 Hz, 3H), 5.90 (s, 1H), 1.26 (s, 9H); ^{13}C NMR (125 MHz, $CDCl_3$, 298 K): δ 145.2 (C_q), 143.7 (C_q), 132.2 (C_q), 130.5 (C_q), 129.9 (C_q), 128.8 (CH), 127.8 (C_q), 127.1 (CH), 126.7 (CH), 126.4 (CH), 126.2 (CH), 123.2 (CH), 122.1 (CH), 119.5 (CH), 54.0 (CH), 47.7 (C_q), 2.6 ($SiCH_3$); HRMS (MALDI-TOF) m/z: $[M]^+$ calcd for $C_{47}H_{34}Si$, 626.2424; found 626.2405, PEG 400, DCTB; m.p. = 335°C (decomp.). R_f = 0.67 (hexane/CH_2Cl_2 = 2:1).

2.8. Synthesis of 3Phe

PH-2-093

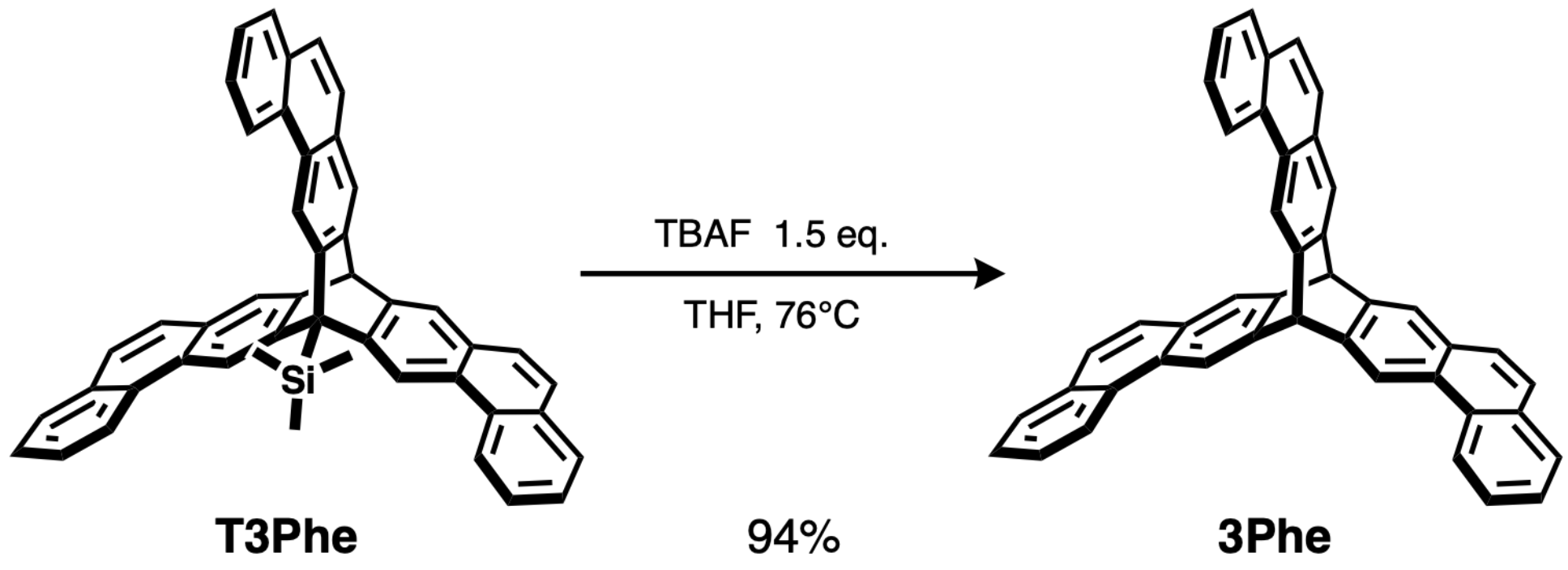

Compound **T3Phe** (35.1 mg, 56.0 μmol) was placed into a 100 mL round-bottom flask, followed by the addition of anhydrous THF (20 mL). To this solution, TBAF (1.0 M in THF, 0.10 mL, 0.10 mmol) was added dropwise under ambient conditions. The resulting mixture was stirred at 76℃ for 18 h. After the reaction was completed, the organic solvent was removed under reduced pressure using a rotary evaporator. The crude product was purified by column chromatography on silica gel (hexane/DCM = 2:1) to give a yellow solid (29.1 mg, 94% yield).

^{1}H NMR (500 MHz, $CDCl_3$, 298 K): δ 8.85 (s, 3H), 8.70 (d, J = 8.3 Hz, 3H), 7.99 (s, 3H), 7.82 (d, J = 8.0 Hz, 3H), 7.69–7.65 (m, 6H), 7.63 (t, J = 7.6 Hz, 3H), 7.53 (t, J = 7.4 Hz, 3H), 6.18 (s, 1H), 5.96 (s, 1H); ^{13}C NMR (125 MHz, $CDCl_3$, 298 K): δ 142.6 (C_q), 142.4 (C_q), 132.2 (C_q), 130.5 (C_q), 130.3 (C_q), 128.6 (CH), 126.9 (C_q), 126.7 (CH), 126.4 (CH), 126.3 (CH), 123.3 (CH), 122.7 (CH), 117.8 (CH), 55.0 (CH), 53.2 (CH). One aromatic CH signal is overlapped; HRMS (MALDI-TOF) m/z: $[M]^+$ calcd for $C_{44}H_{26}$, 554.2029; found 554.2031, PEG 400, DCTB; m.p. = 281.0–282.1°C. R_f = 0.73 (hexane/CH_2Cl_2 = 2:1).

3. Single crystal X-ray diffraction measurements

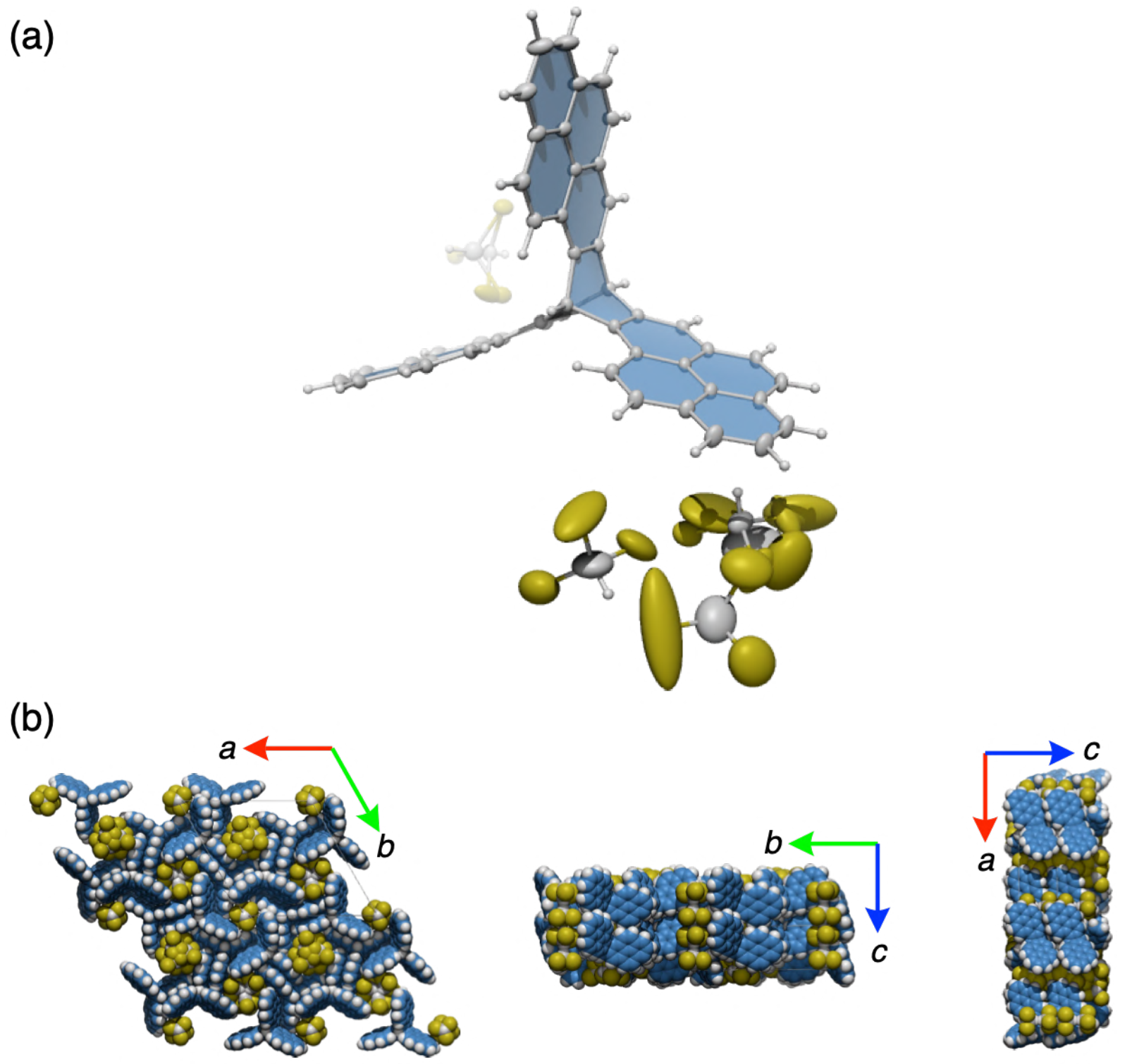

Figure S2. (a) ORTEP drawing structure of **3Py** with $CHCl_3$ and (b) its packing structures. Thermal ellipsoids are drawn at 50% probability. Gray: carbon, white: hydrogen, and yellow: chlorine.

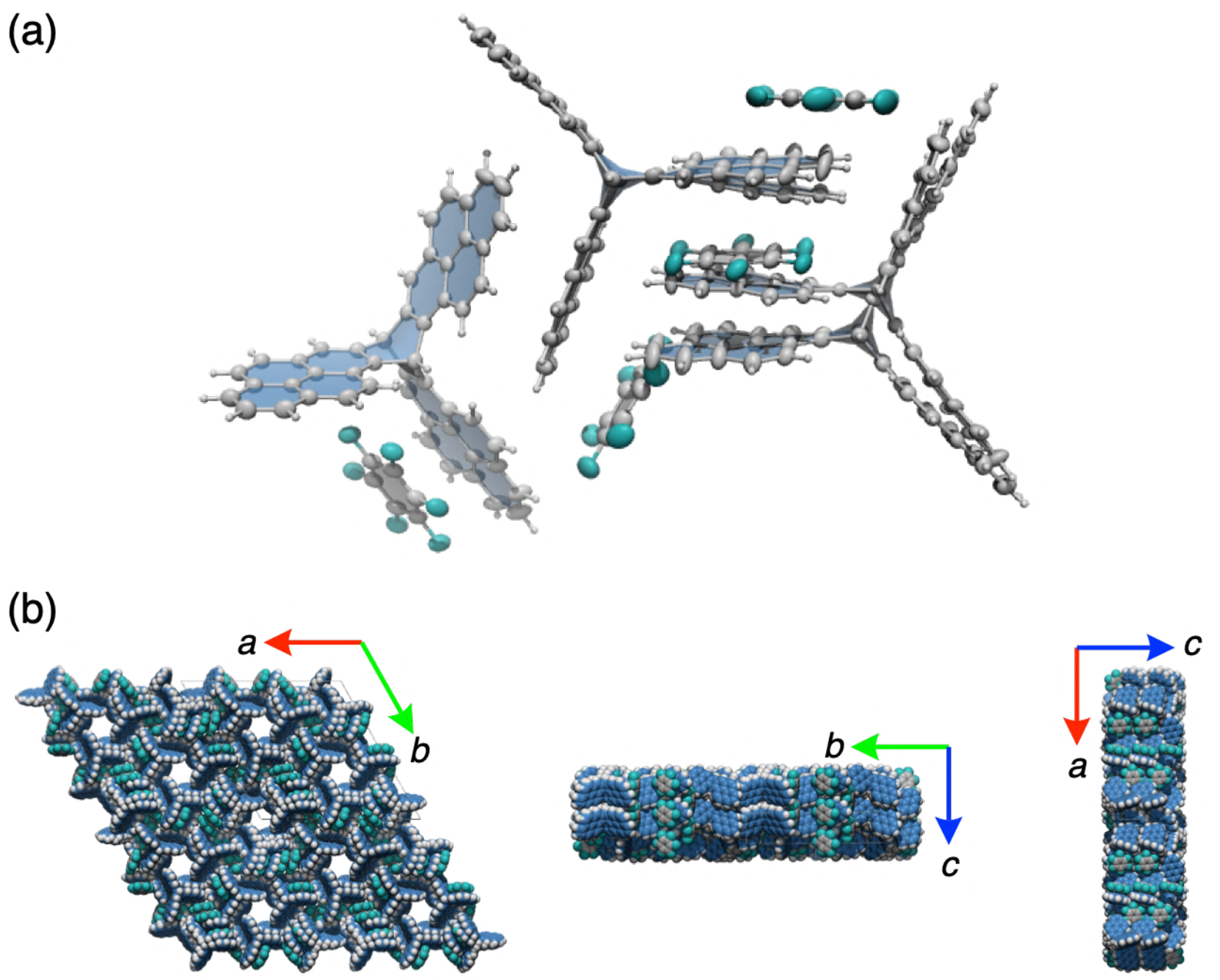

Figure S3. (a) ORTEP drawing structure of **3Py** with C_6F_6 and (b) its packing structures. Thermal ellipsoids are drawn at 50% probability. Gray: carbon, white: hydrogen, and green: fluorine.

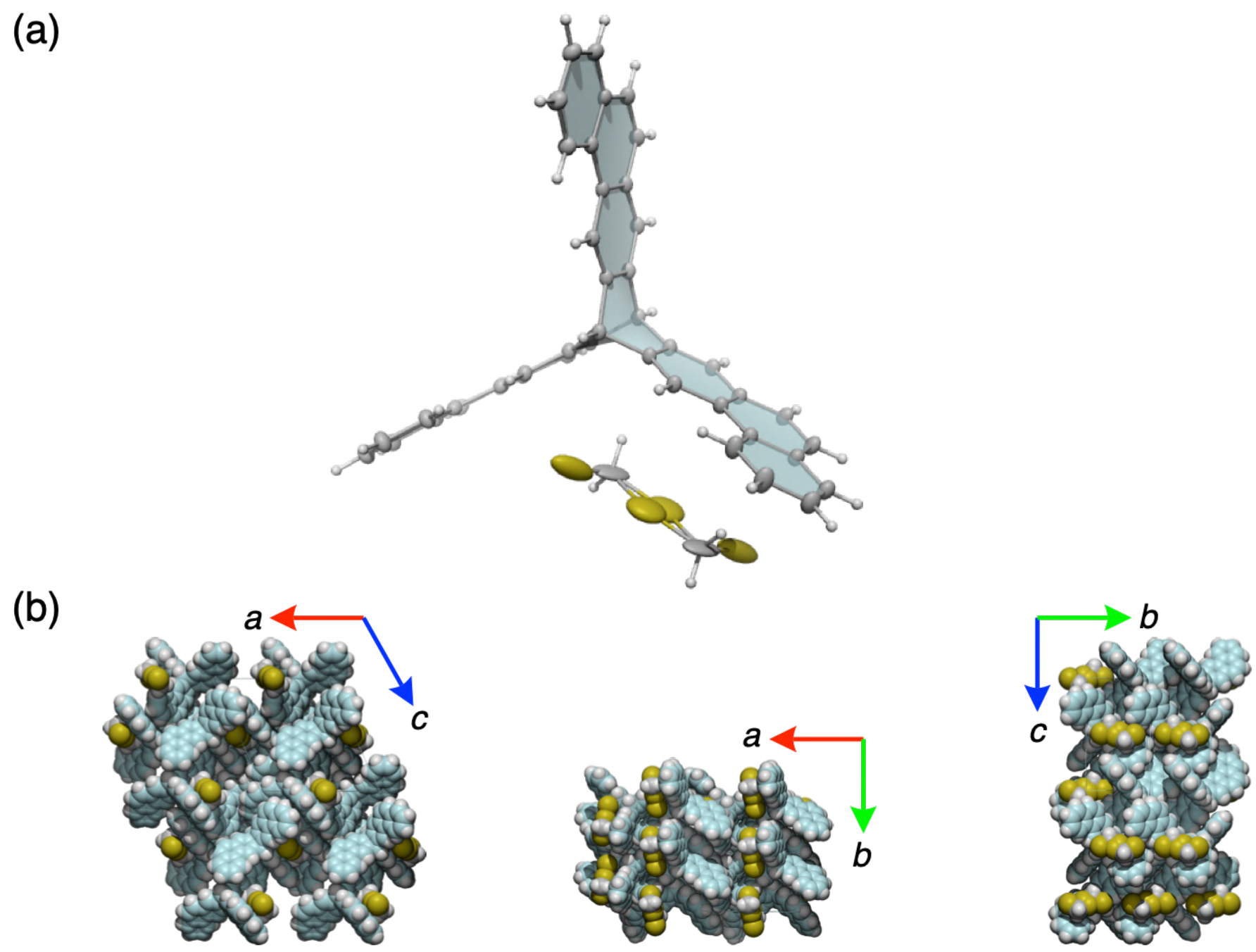

Figure S4. (a) ORTEP drawing structure of **3Phe** with CH_2Cl_2 and (b) its packing structures. Thermal ellipsoids are drawn at 50% probability. Gray: carbon, white: hydrogen, and yellow: chlorine.

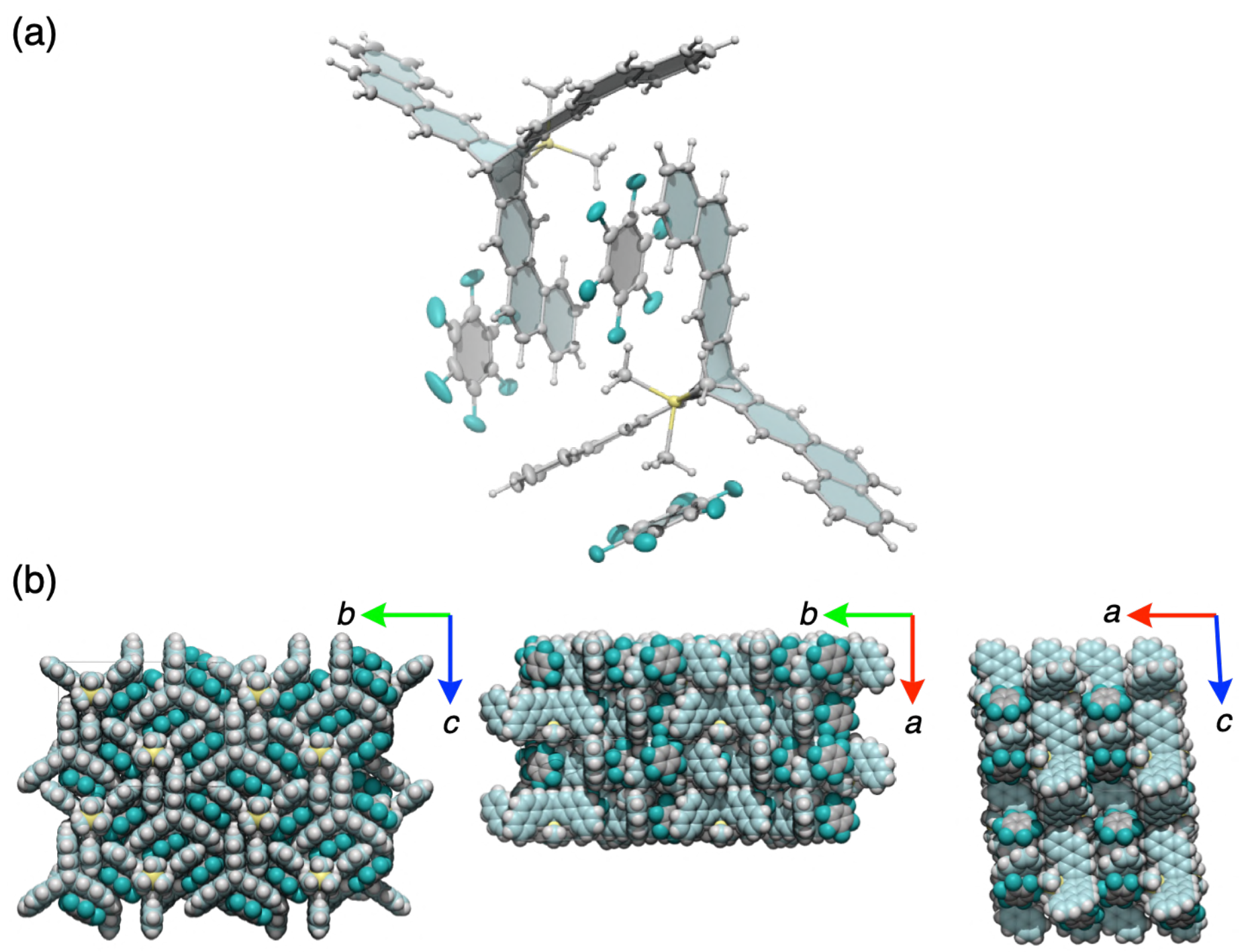

Figure S5. (a) ORTEP drawing structure of **T3Phe** with C_6F_6 and (b) its packing structures. Thermal ellipsoids are drawn at 50% probability. Gray: carbon, white: hydrogen, green: fluorine, and yellow: silicon.

Table S1. Crystal data and structure refinement of **3Py**•$CHCl_3$ and **3Py**•C_6F_6.

Compounds	**3Py**•$CHCl_3$	**3Py**•C_6F_6
Identification code	3Py_CHCl3	3Py_C6F6
CCDC number	2535452	2535453
Empirical formula	$C_{76.87}H_{40.87}Cl_{5.62}$	$C_{243.06}H_{117.16}F_{17.91}$*
Formula weight	1163.58	3377.52
Temperature (K)	95.00	95.00
Wavelength (Å)	0.750	0.750
Crystal system	trigonal	trigonal
Space group	*P*-3	*P*-3
Unit cell dimensions		
a (Å)	27.9900(14)	48.908(10)
b (Å)	27.9900(14)	48.908(10)
c (Å)	8.2090(13)	8.157(4)
α (°)	90	90
β (°)	90	90
γ (°)	120	120
Volume (Å^3)	5569.6(10)	16897(11)
Z	4	4
Density (calculated, g cm^{-3})	1.388	1.328
Absorption coefficient (mm^{-1})	0.390	0.101
F(000)	2390.0	6947.0
Crystal size (mm^3)	0.8 × 0.2 × 0.2	0.2 × 0.3 × 0.3
2θ range for data collection (°)	1.772 to 71.112	1.758 to 71.142
Index ranges	$-41 \le h \le 41$	$-74 \le h \le 75$
	$-41 \le k \le 38$	$-75 \le k \le 63$
	$-9 \le l \le 9$	$-12 \le l \le 12$
Reflections collected	60428	183257
Independent reflections	12209 [R_{int} = 0.0110]	42574 [R_{int} = 0.1224]
Completeness to θ	0.842	0.947
Absorption correction	none	none
Refinement method	Full-matrix least-squares on *F*	
Data / restraints / parameters	12209/287/704	42574/3/2298
Goodness-of-fit on F^2	1.059	1.083
Final *R* indices [$I > 2\sigma(I)$]	R_1 = 0.0659	R_1 = 0.1228
	wR_2 = 0.1857	wR_2 = 0.3433
R indices (all data)	R_1 = 0.0668	R_1 = 0.2332
	wR_2 = 0.1865	wR_2 = 0.4043
Largest diff. peak and hole	0.74 and –0.65 eÅ^{-3}	0.47 and –0.44 eÅ^{-3}

*: Highly disordered solvent molecules were treated using a solvent mask and are not included in the empirical formula.

Table S2. Crystal data and structure refinement of **3Phe** and **T3Phe**.

Compounds	**3Phe**	**T3Phe**
Identification code	3Phe	T3Phe
CCDC number	2535454	2535455
Empirical formula	$C_{44.08}H_{26.16}Cl_{0.15}$	$C_{112}F_{18}Si_2H_{68}$
Formula weight	561.23	1811.84
Temperature (K)	95.00	95.00
Wavelength (Å)	0.750	0.750
Crystal system	monoclinic	monoclinic
Space group	$P2_1$/n	$P2_1$/n
Unit cell dimensions		
a (Å)	17.745(4)	16.0360(12)
b (Å)	9.813(2)	26.5440(6)
c (Å)	19.115(4)	19.8830(9)
α (°)	90	90
β (°)	114.84(3)	95.173(4)
γ (°)	90	90
Volume (Å^3)	3020.6(12)	8428.9(8)
Z	4	4
Density (calculated, g cm^{-3})	1.234	1.428
Absorption coefficient (mm^{-1})	0.093	0.155
F(000)	1173.0	3720.0
Crystal size (mm^3)	0.2 × 0.15 × 0.15	0.3 × 0.05 × 0.05
2θ range for data collection (°)	2.776 to 71.128	2.708 to 71.14
Index ranges	$-27 \leq h \leq 27$	$-24 \leq h \leq 24$
	$-15 \leq k \leq 15$	$-41 \leq k \leq 40$
	$-26 \leq l \leq 29$	$-29 \leq l \leq 29$
Reflections collected	32056	90506
Independent reflections	11174 [R_{int} = 0.0148]	31205 [R_{int} = 0.0126]
Completeness to θ	0.947	0.947
Absorption correction	none	none
Refinement method	Full-matrix least-squares on F	
Data / restraints / parameters	11174/0/425	31205/18/1195
Goodness-of-fit on F^2	1.050	1.021
Final R indices [$I > 2\sigma(I)$]	R_1 = 0.0495	R_1 = 0.0468
	wR_2 = 0.1392	wR_2 = 0.1332
R indices (all data)	R_1 = 0.0510	R_1 = 0.0507
	wR_2 = 0.1411	wR_2 = 0.1368
Largest diff. peak and hole	0.40 and –0.39 eÅ^{-3}	0.85 and –0.75 eÅ^{-3}

4. DFT calculations

4.1. Ground-state optimized structures

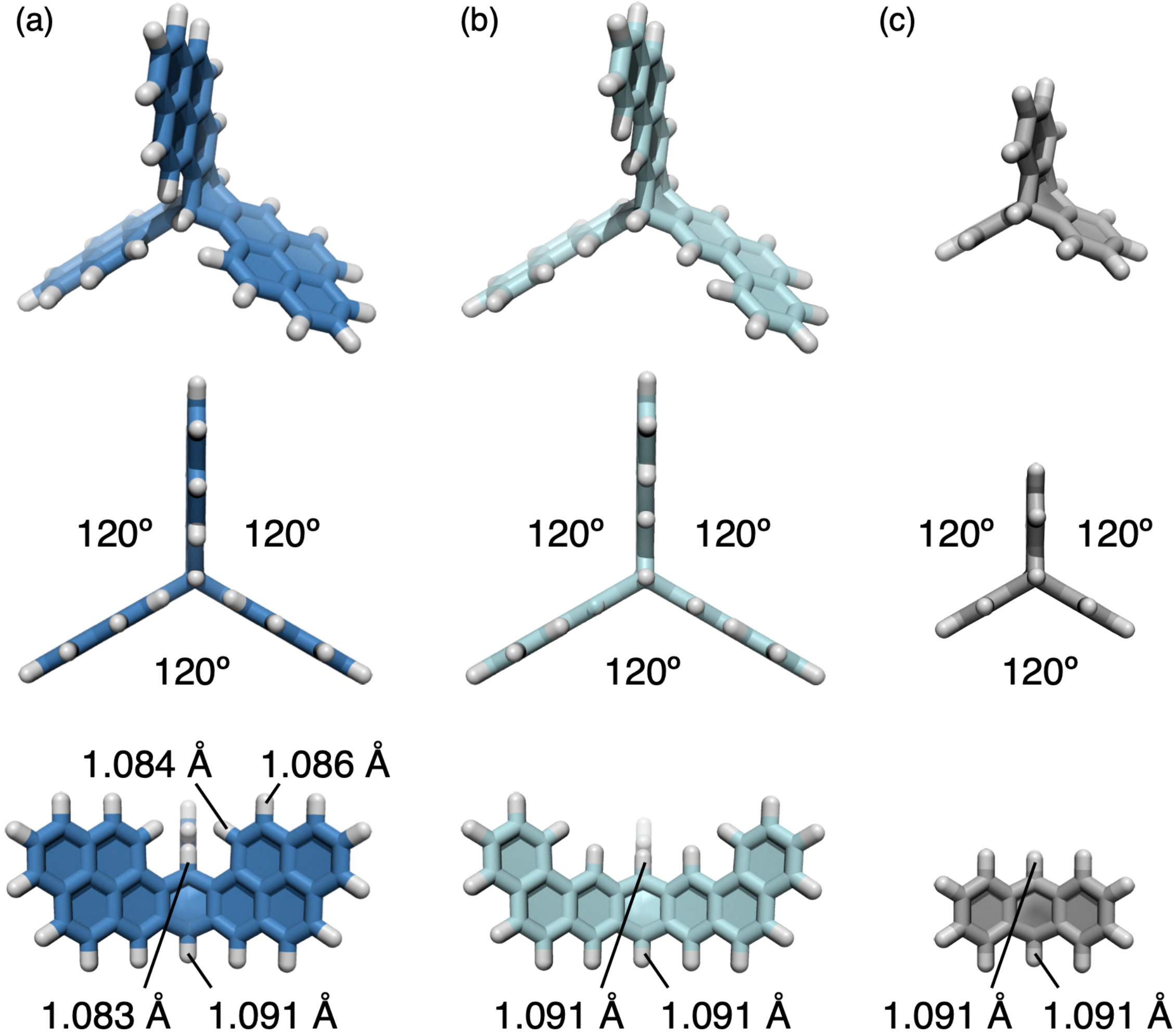

Figure S6. Optimized structures of (a) **3Py**, (b) **3Phe**, and (c) triptycene, calculated at the B3LYP-D3(BJ)/6-31G(d,p) (PCM, $CHCl_3$) level. Front views (top), top views (middle), and side views (bottom) are shown. Interpanel angles were defined as the angular sectors between the PAH centroids projected onto a plane perpendicular to the bridgehead C–C axis. Bridgehead C–H distances are shown in Å.

4.2. NMR analyses

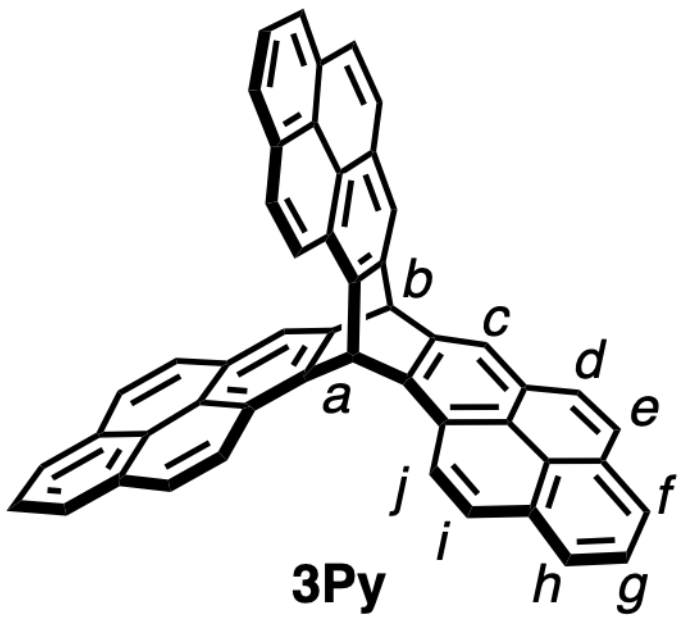

3Py

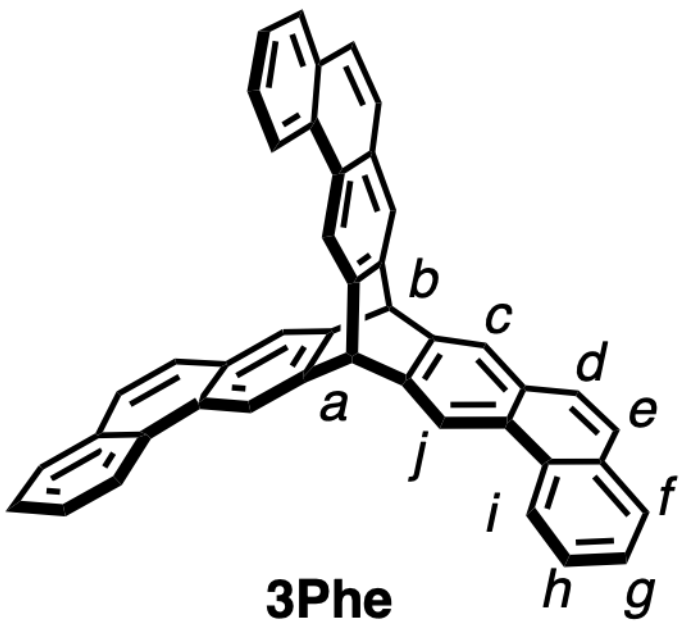

3Phe

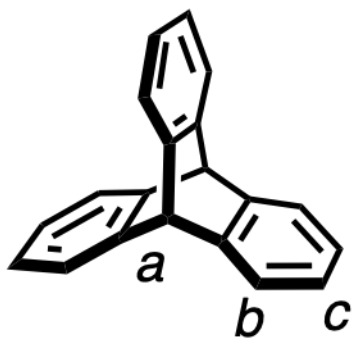

triptycene

Table S3a. Calculated ^{1}H and ^{13}C NMR chemical shifts for **3Py**, **3Phe**, and triptycene obtained by GIAO calculations at the HF/6-311+G(d,p), B3LYP/6-311+G(d,p), and B97-2/cc-PVTZ levels, using the optimized structure at the B3LYP-D3(BJ)/6-31G(d,p) level. All calculations were performed using PCM($CHCl_3$). Some experimental ^{13}C NMR signals could not be assigned due to signal overlap.

	**3Py** / ppm				**3Phe** / ppm				triptycene / ppm			
H	δ_{exp}	HF	B3LYP	B97-2	δ_{exp}	HF	B3LYP	B97-2	δ_{exp}[S10]	HF	B3LYP	B97-2
a	8.68	9.04	9.21	9.37	6.18	6.46	6.32	6.46	5.45	5.56	5.49	5.57
b	6.66	7.02	6.91	7.02	5.96	6.17	6.06	6.22	7.41	7.90	7.66	7.68
c	8.50	9.30	8.94	8.97	7.99	8.66	8.35	8.39	7.01	7.45	7.31	7.31
d	8.01	8.77	8.48	8.54	7.66	8.31	8.06	8.13				
e	8.00	8.72	8.44	8.50	7.66	8.29	8.05	8.11				
f	8.09	8.81	8.51	8.56	7.82	8.47	8.20	8.25				
g	7.91	8.56	8.33	8.38	7.53	8.07	7.87	7.93				
h	8.14	8.85	8.55	8.61	7.63	8.19	8.00	8.05				
i	8.26	9.01	8.72	8.78	8.71	9.50	9.29	9.31				
j	9.07	9.93	9.72	9.81	8.85	9.66	9.39	9.41				

	**3Py** / ppm				**3Phe** / ppm				triptycene / ppm			
C	δ_{exp}	HF	B3LYP	B97-2	δ_{exp}	HF	B3LYP	B97-2	δ_{exp}[S10]	HF	B3LYP	B97-2
a	40.6	39.3	48.0	46.8	55.0	51.6	62.9	61.2	54.2	51.0	62.4	60.4
b	56.7	53.4	64.9	63.1	53.2	49.9	61.1	59.5	123.7	131.7	129.8	126.6
c	120.8	127.7	126.4	123.5	123.3	130.2	129.2	126.3	125.3	133.7	131.9	128.1
d	127.3	134.8	134.2	131.1	-	134.2	133.5	130.3				
e	127.3	134.5	134.0	130.8	-	133.9	133.5	130.2				
f	-	133.1	131.3	128.1	128.6	136.9	135.3	131.9				
g	-	133.8	132.5	128.4	-	134.6	132.7	129.3				
h	124.9	132.5	130.8	127.7	-	134.9	132.9	129.3				
i	128.5	135.9	135.0	131.7	122.7	131.4	128.8	125.7				
j	122.1	130.3	129.0	125.9	117.8	125.6	123.3	120.6				

Table S3b. Calculated NICS(1)$_{zz}$ values for **Py**, **3Py**, **Phe**, and **3Phe** obtained by GIAO calculations at the B3LYP-D3(BJ)/6-31G(d,p) (PCM, $CHCl_3$) level.

	**Py**	**3Py**	**Phe**	**3Phe**
A	–35.7	–30.9	–29.2	–24.5
B	–16.6	–16.1	–20.7	–20.1
C	–35.7	–34.3	–29.2	–28.3
D	–16.6	–13.4	–	–

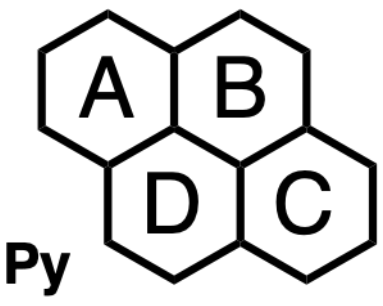

Py

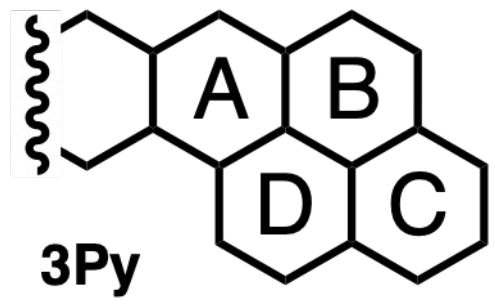

3Py

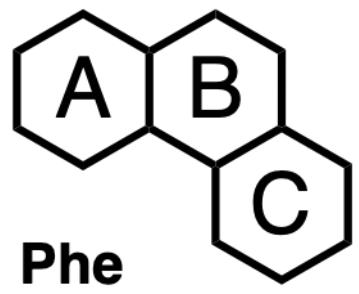

Phe

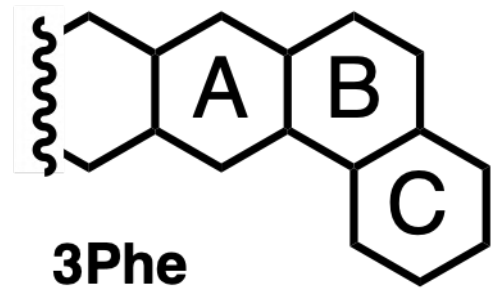

3Phe

4.3. TD-DFT calculations

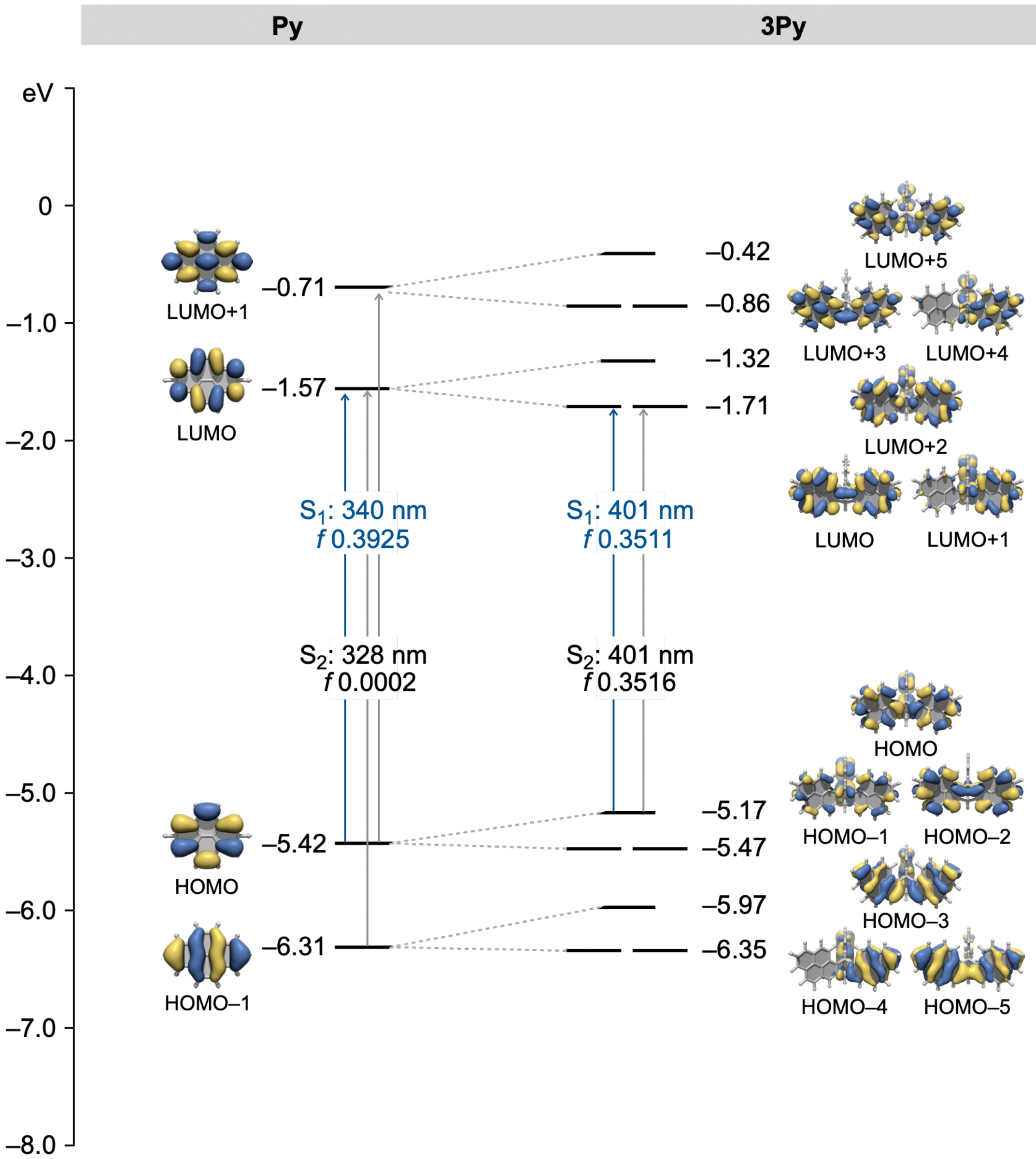

Figure S7. Frontier molecular orbital energy diagrams and selected electronic transitions of **Py** and **3Py**, calculated at the B3LYP-D3(BJ)/6-31G(d,p) (PCM, $CHCl_3$) level. The corresponding HOMO−1, HOMO, LUMO, and LUMO+1 orbitals are also shown. The $S_0 \rightarrow S_1$ and $S_0 \rightarrow S_2$ transitions are indicated by blue and gray arrows, respectively, together with their excitation wavelengths and oscillator strengths (*f*).

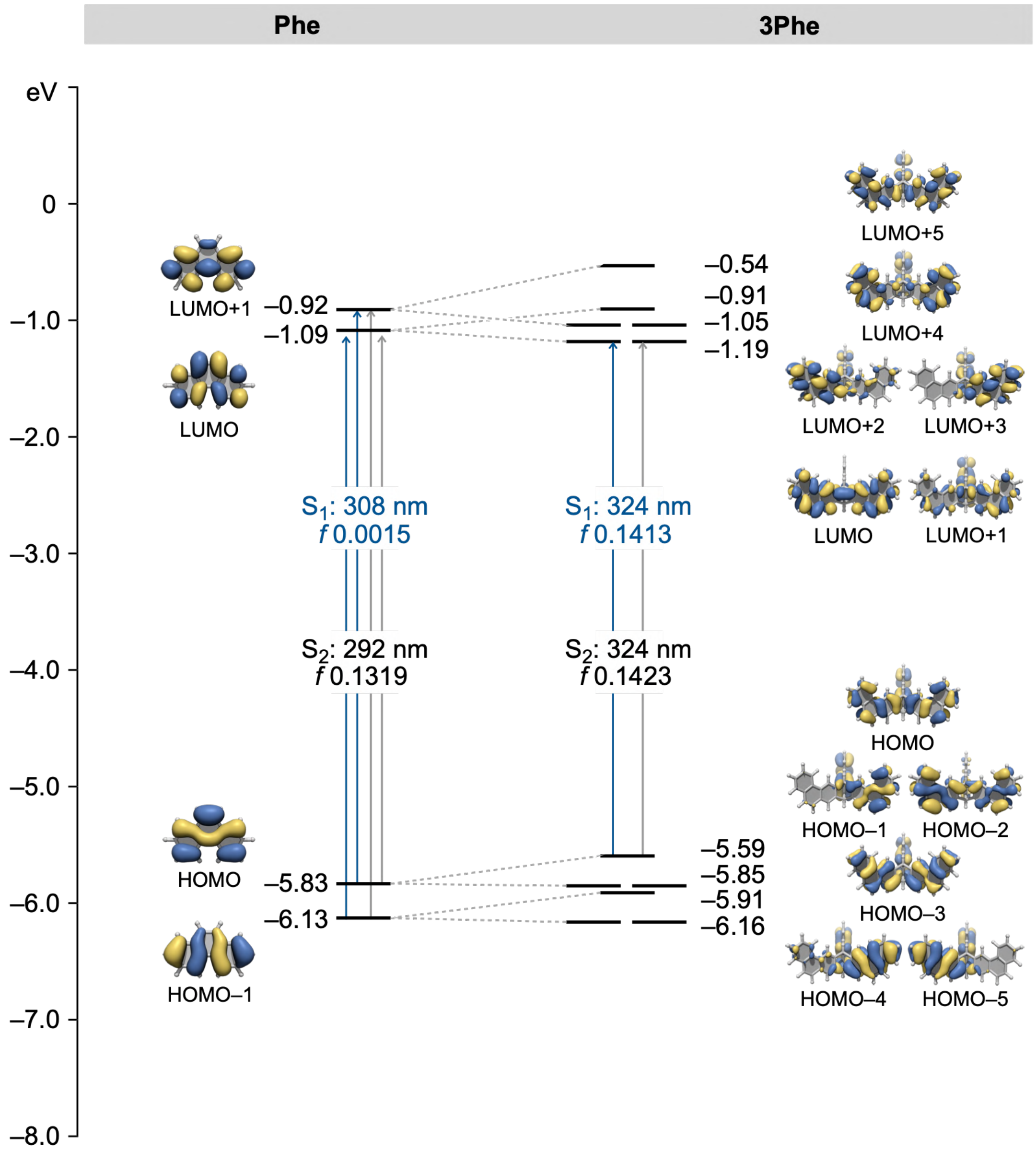

Figure S8. Frontier molecular orbital energy diagrams and selected electronic transitions of **Phe** and **3Phe**, calculated at the B3LYP-D3(BJ)/6-31G(d,p) (PCM, $CHCl_3$) level. The corresponding HOMO−1, HOMO, LUMO, and LUMO+1 orbitals are also shown. The $S_0 \rightarrow S_1$ and $S_0 \rightarrow S_2$ transitions are indicated by blue and gray arrows, respectively, together with their excitation wavelengths and oscillator strengths (*f*).

Table S4. Selected excitation data for the absorption of **Py** obtained from TD-DFT calculations at the B3LYP-D3(BJ)/6-31G(d,p) (PCM, $CHCl_3$) level.

excited state	energy/eV	λ/nm	f [a]	transition assignment [b]
1	3.65	340	0.3925	H→L(93.2%)
2	3.79	328	0.0002	H–1→L(46.8%) H→L+1(52.8%)
3	4.44	279	0.0000	H→L+2(89.4%)
4	4.58	271	0.3841	H–1→L(50.6%) H→L+1(44.8%)
5	4.65	267	0.0000	H–2→L(89.0%)
6	5.12	242	0.0000	H–3→L(42.8%) H→L+3(46.7%)
7	5.28	235	0.0000	H–2→L+1(39.0%) H–1→L+2(50.6%)
8	5.37	231	1.0097	H–1→L+1(90.1%)
9	5.69	218	0.0000	H–4→L(36.4%) H–3→L+1(33.2%)<br>H–1→L+3(18.7%) H→L+4(10.9%)
10	5.78	215	0.0000	H–3→L(43.9%) H→L+3(40.4%)

[a] Oscillator strength. [b] H: HOMO, L: LUMO. Only transitions with contributions >10% are listed.

Table S5. Selected excitation data for the absorption of **3Py** obtained from TD-DFT calculations at the B3LYP-D3(BJ)/6-31G(d,p) (PCM, $CHCl_3$) level.

excited state	energy/eV	λ/nm	f [a]	transition assignment [b]
1	3.09	401	0.3511	H→L(96.2%)
2	3.09	401	0.3516	H→L+1(96.1%)
3	3.34	371	0.0001	H–2→L+1(42.1%) H–1→L(44.3%)
4	3.39	366	0.3213	H–2→L(45.5%) H–1→L+1(45.3%)
5	3.39	366	0.3209	H–2→L+1(46.3%) H–1→L(44.5%)
6	3.41	364	0.0143	H–2→L(19.0%)<br>H–1→L+1(18.7%) H→L+2(54.5%)
7	3.59	345	0.0006	H–3→L(16.2%)<br>H–3→L+1(23.5%) H→L+3(37.4%)
8	3.59	345	0.0006	H–3→L(23.6%)<br>H–3→L+1(16.2%) H→L+4(37.3%)
9	3.66	338	0.1536	H–2→L(21.5%)<br>H–1→L+1(21.4%) H→L+2(37.9%)
10	3.75	331	0.0017	H–5→L(16.5%) H–4→L+1(16.5%)<br>H–3→L+2(13.1%) H–2→L+4(17.4%)<br>H–1→L+3(17.3%) H→L+5(11.3%)

[a] Oscillator strength. [b] H: HOMO, L: LUMO. Only transitions with contributions >10% are listed.

Table S6. Selected excitation data for the absorption of **Phe** obtained from TD-DFT calculations at the B3LYP-D3(BJ)/6-31G(d,p) (PCM, $CHCl_3$) level.

excited state	energy/eV	λ/nm	f [a]	transition assignment [b]
1	4.02	308	0.0015	H–1→L(40.7%) H→L+1(58.4%)
2	4.25	292	0.1319	H–1→L+1(17.1%) H→L(81.3%)
3	4.78	260	0.1830	H–1→L(54.8%) H→L+1(39.0%)
4	4.97	250	0.9998	H–1→L+1(80.3%) H→L(16.0%)
5	5.21	238	0.0050	H–2→L(44.1%) H→L+2(54.3%)
6	5.25	236	0.0094	H–2→L+1(47.4%) H–1→L+2(41.8%)
7	5.84	212	0.0128	H–3→L(54.1%) H→L+3(35.5%)
8	5.90	210	0.1911	H–2→L(47.8%) H→L+2(36.0%)
9	5.96	208	0.4791	H–2→L+1(35.3%) H–1→L+2(34.7%) H→L+3(18.7%)
10	6.15	202	0.0087	H–3→L+1(53.7%) H–1→L+3(42.7%)

[a] Oscillator strength. [b] H: HOMO, L: LUMO. Only transitions with contributions >10% are listed.

Table S7. Selected excitation data for the absorption of **3Phe** obtained from TD-DFT calculations at the B3LYP-D3(BJ)/6-31G(d,p) (PCM, $CHCl_3$) level.

excited state	energy/eV	λ/nm	f [a]	transition assignment [b]
1	3.82	324	0.1413	H→L(37.4%) H→L+2(20.1%)
2	3.83	324	0.1423	H→L+1(37.4%) H→L+3(20.4%)
3	3.99	311	0.0041	H–3→L+4(11.4%) H–2→L+3(14.6%) H–1→L+2(15.4%)
4	3.99	311	0.1619	H→L(39.3%) H→L+2(20.1%) H→L+3(16.6%)
5	3.99	311	0.1594	H→L+1(38.8%) H→L+2(17.0%) H→L+3(20.1%)
6	4.19	296	0.0334	H–2→L+1(18.3%) H–1→L(31.5%) H→L+4(25.1%)
7	4.19	296	0.0300	H–2→L(22.6%) H–1→L+1(26.7%)
8	4.19	296	0.0285	H–2→L+1(31.0%) H–1→L(21.6%)
9	4.25	292	0.0001	H–2→L(48.7%) H–1→L+1(47.5%)
10	4.27	290	0.2474	H–2→L(15.5%) H–1→L+1(14.7%) H→L+2(29.4%)

[a] Oscillator strength. [b] H: HOMO, L: LUMO. Only transitions with contributions >10% are listed.

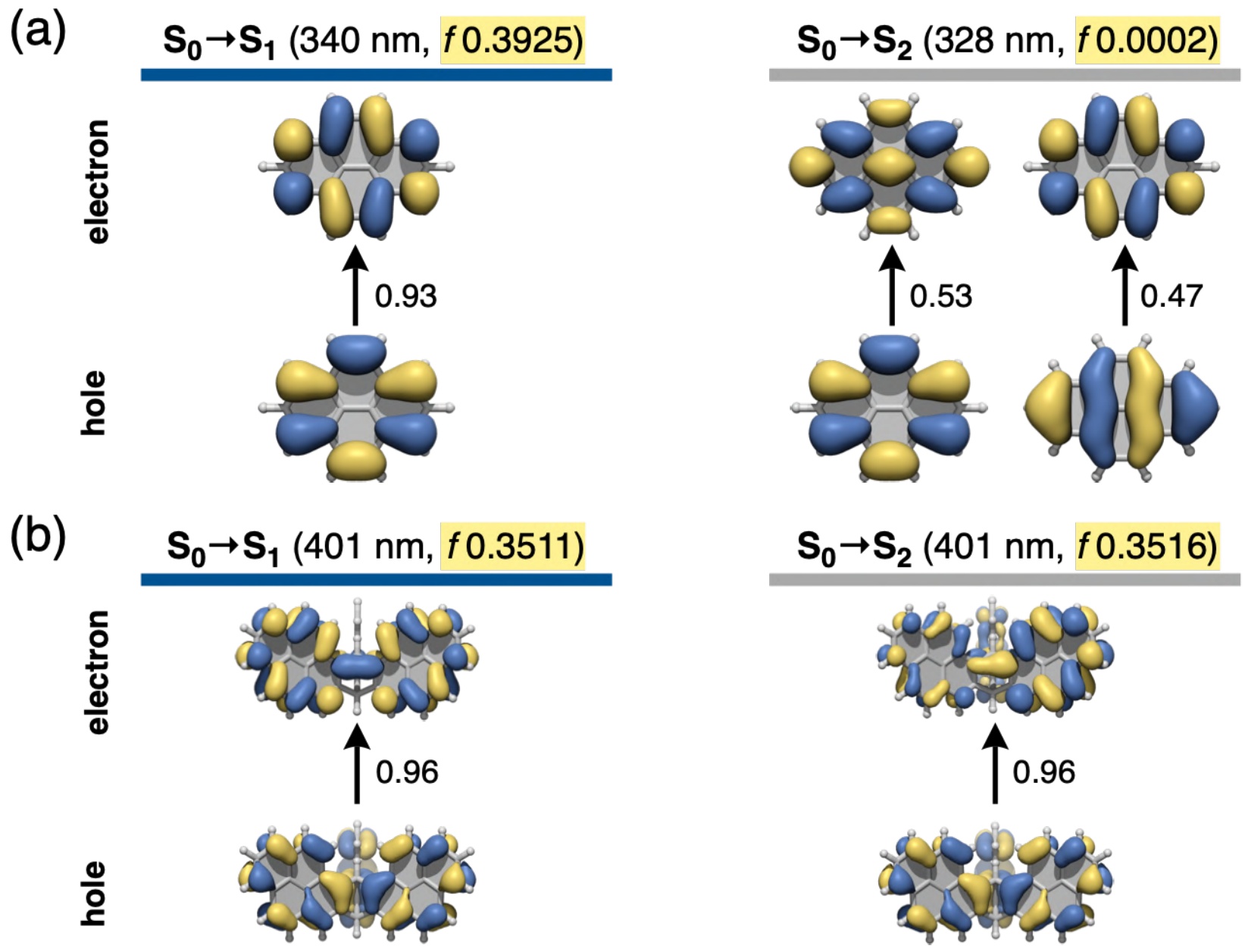

Figure S9. Optimized structures and corresponding NTOs for the S_1 and S_2 states of (a) **Py** and (b) **3Py**, calculated at the B3LYP-D3(BJ)/6-31G(d,p) (PCM, $CHCl_3$) level.

5. Optical properties

5.1. UV–vis and fluorescence properties

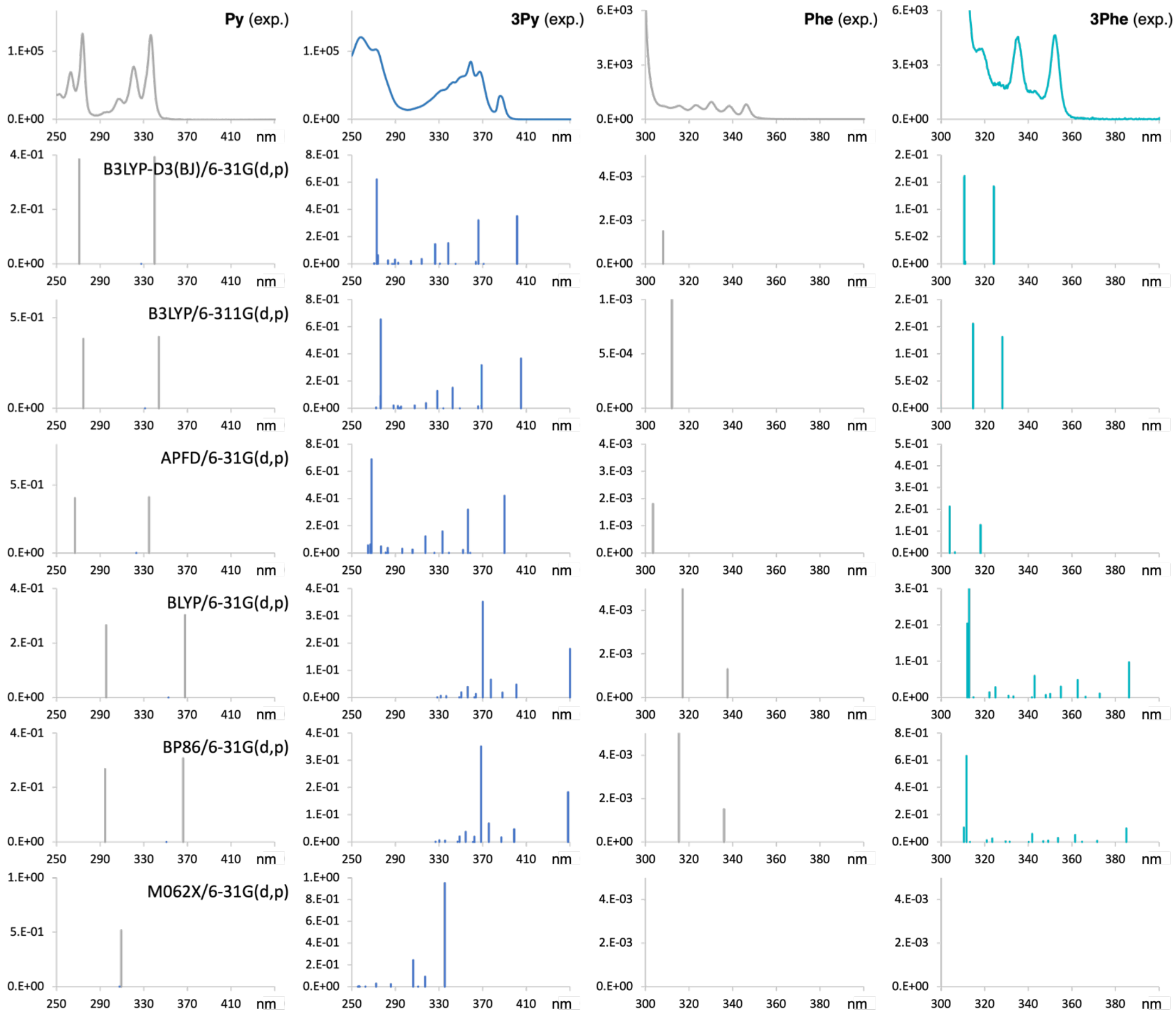

Figure S10. Screening of density functionals and basis sets for TD-DFT calculations of **Py**, **3Py**, **Phe**, and **3Phe** in $CHCl_3$. BLYP, previously reported to reproduce the excitation energies of **Phe**,[S11] was included. All calculations were performed using the optimized structures at the B3LYP-D3(BJ)/6-31G(d,p) (PCM, $CHCl_3$) level.

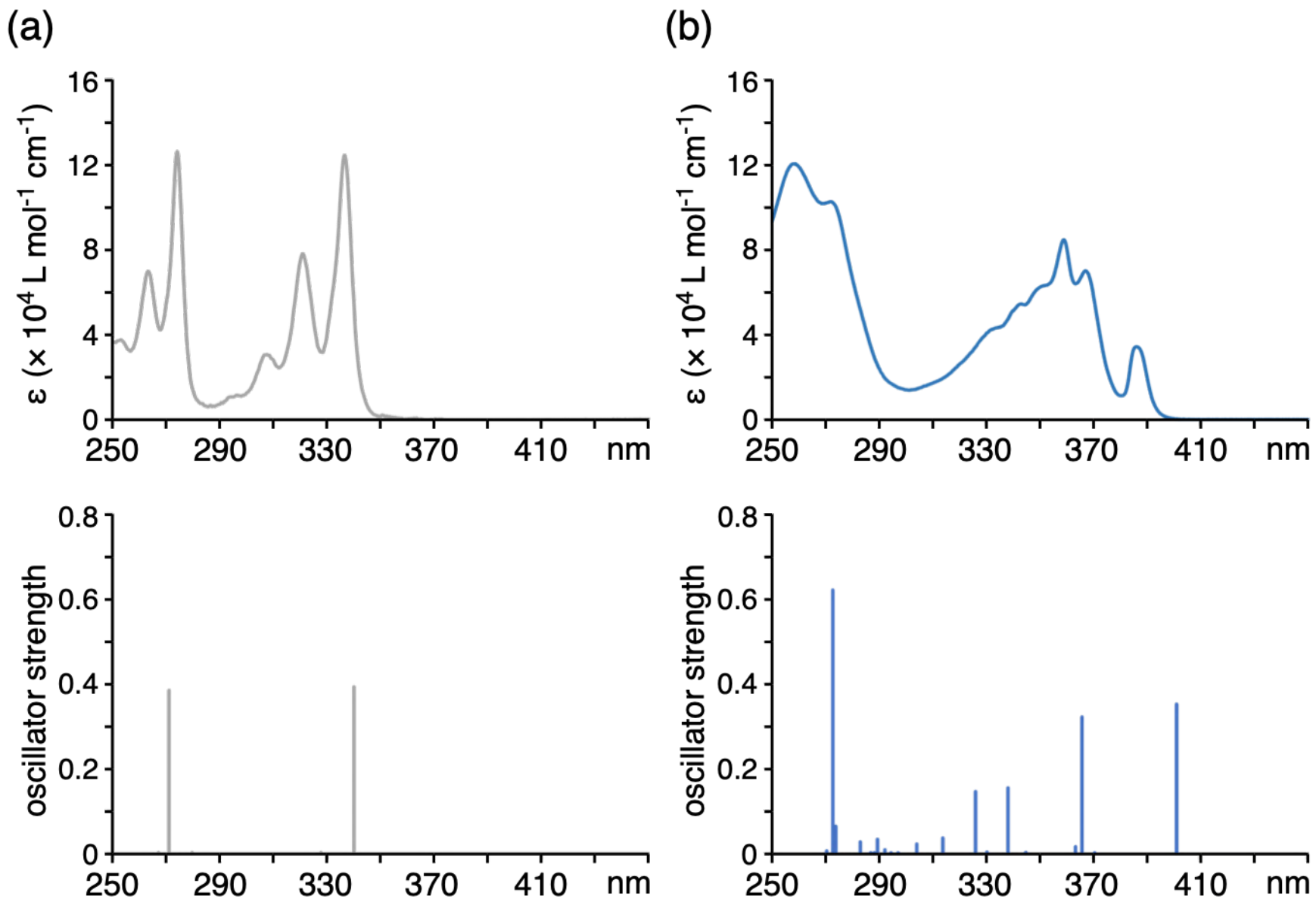

Figure S11. UV–vis spectra of (a) **Py** and (b) **3Py** in the 250–450 nm region ($CHCl_3$, 1.0 × 10^{-5} M) with calculated electronic transitions shown as sticks. The molar absorption coefficient (ε) of **Py** was multiplied by a factor of three to account for the three pyrene units in **3Py**. TD-DFT calculations were performed at the B3LYP-D3(BJ)/6-31G(d,p) (PCM, $CHCl_3$) level.

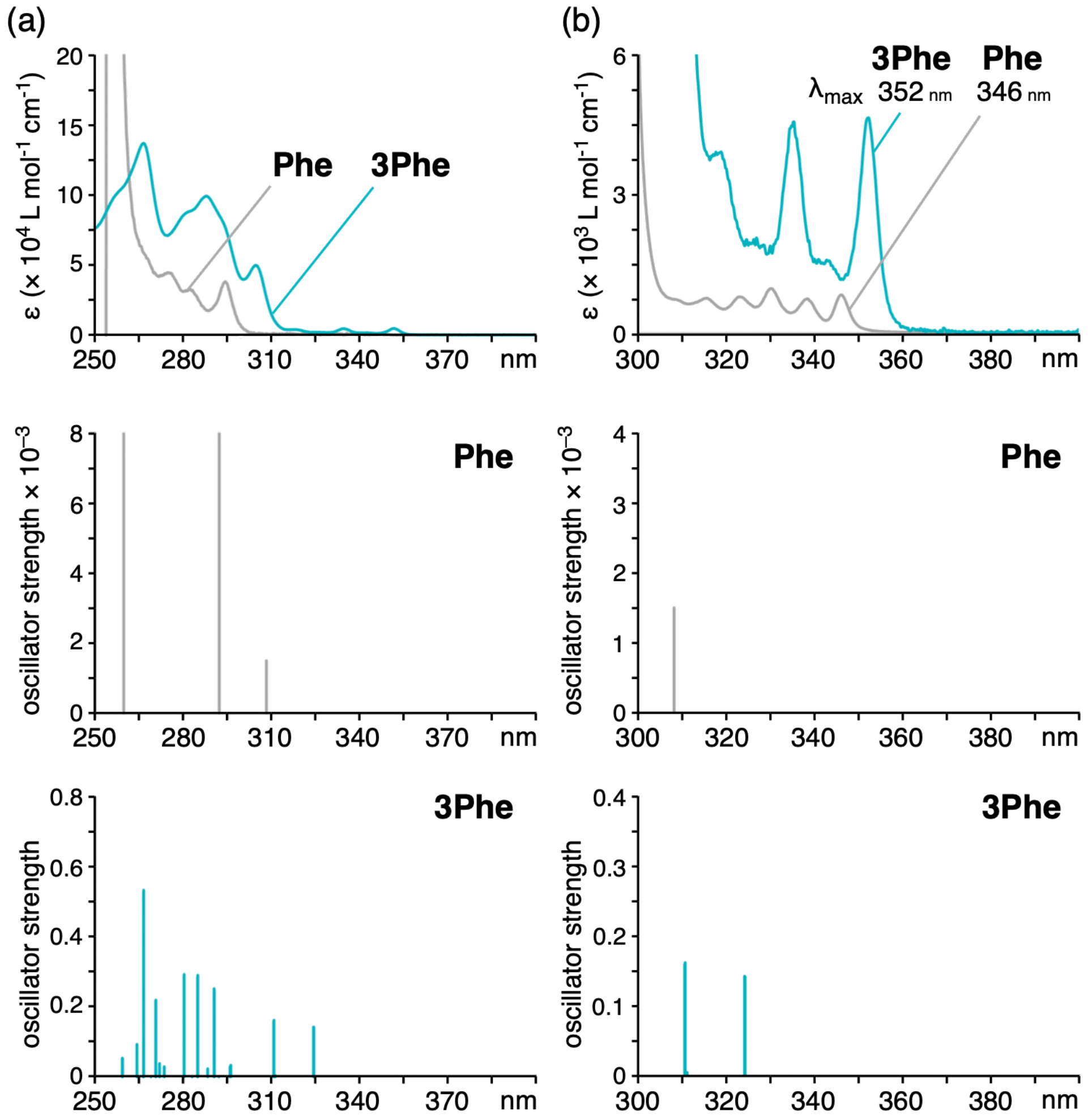

Figure S12. UV–vis spectra of **Phe** and **3Phe** in (a) the 250–400 nm region ($CHCl_3$, 1.0 × 10^{-5} M for both **Phe** and **3Phe**) and (b) the 300–400 nm region ($CHCl_3$, **Phe**: 1.0 × 10^{-3} M, **3Phe**:1.0 × 10^{-5} M) with calculated electronic transitions shown as sticks. The molar absorption coefficient (*ε*) of **Phe** was multiplied by a factor of three to account for the three phenanthrene units in **3Phe**. TD-DFT calculations were performed at the B3LYP-D3(BJ)/6-31G(d,p) (PCM, $CHCl_3$) level.

Table S8. Fluorescence parameters of **Py**, **3Py**, **Phe**, and **3Phe** ($CHCl_3$, 1.0 × 10^{-5} M). τ: fluorescence lifetime; Φ_F: fluorescence quantum yield; k_r and k_{nr}: radiative and nonradiative decay rate constants, respectively, calculated as $k_r = \Phi_F/\tau$ and $k_{nr} = (1-\Phi_F)/\tau$.

compound	τ (ns)	Φ_F	k_r (s^{-1})	k_{nr} (s^{-1})
**Py**	49	0.12	2.5×10^6	18×10^6
**3Py**	7.5	0.57	77×10^6	58×10^6
**Phe**	16	0.06	3.8×10^6	59×10^6
**3Phe**	11	0.14	13×10^6	79×10^6

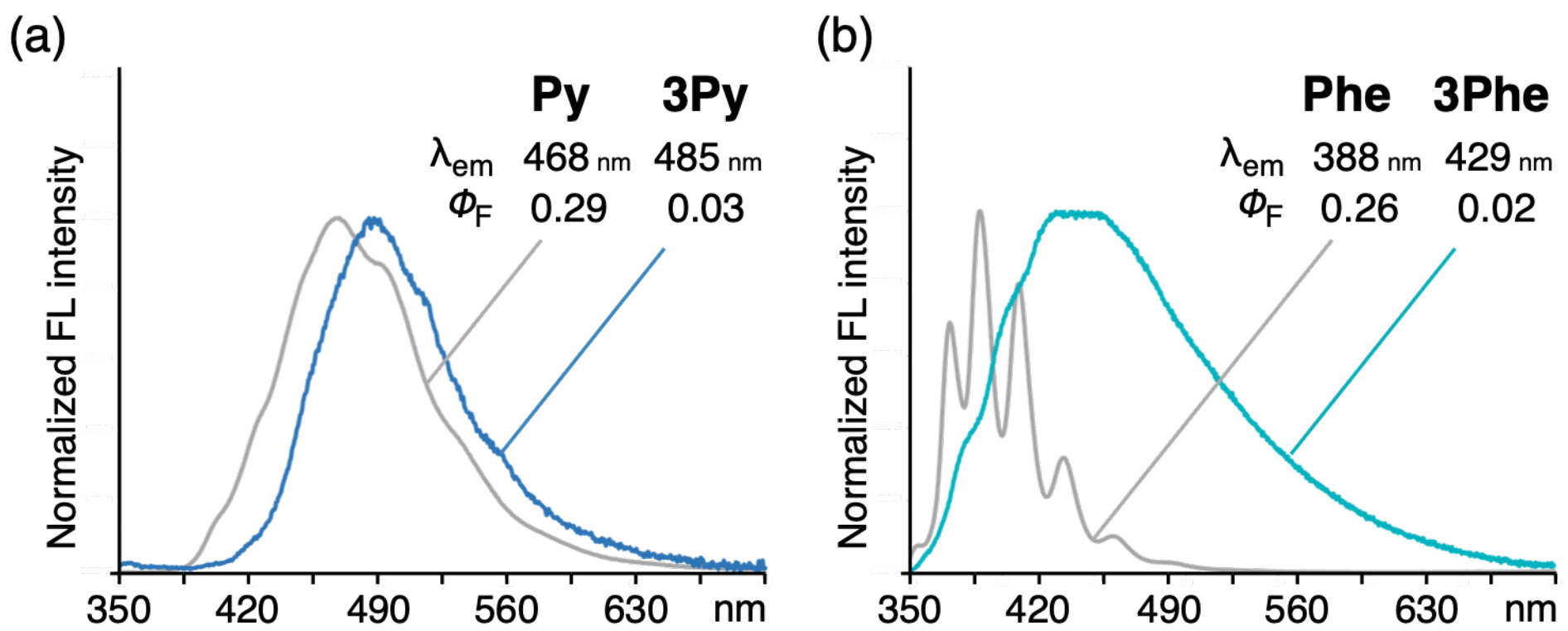

Figure S13. Solid-state fluorescence spectra of (a) **Py** and **3Py** and (b) **Phe** and **3Phe** (λ_{ex} = 300 nm).

5.2. Excited-state optimized structures

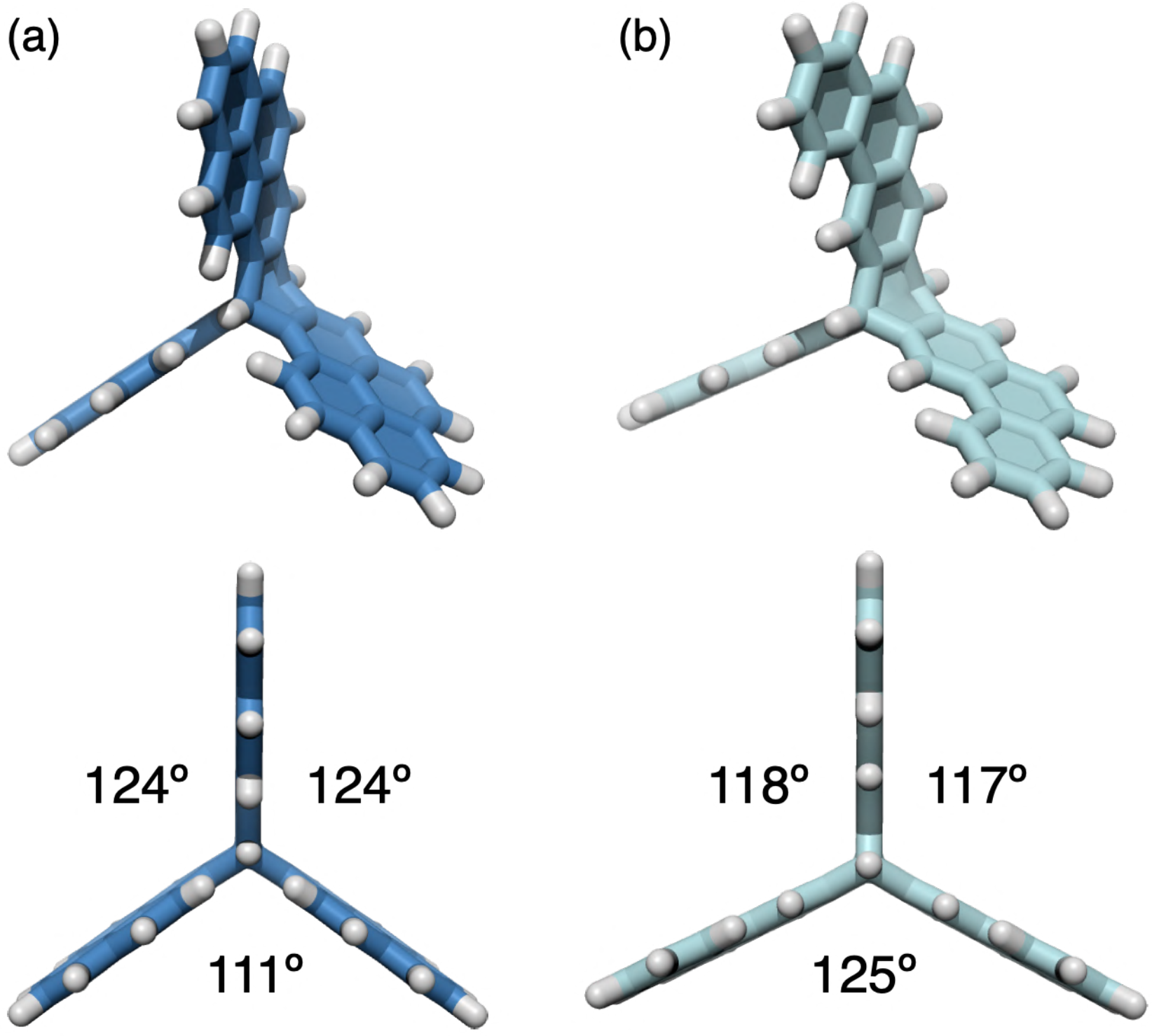

Figure S14. S_1-state optimized structures of (a) **3Py** and (b) **3Phe**, calculated at the B3LYP-D3(BJ)/6-31G(d,p) (PCM, $CHCl_3$) level. Side views (top) and top views (bottom) are shown. Interpanel angles were defined as the angular sectors between the PAH centroids projected onto a plane perpendicular to the bridgehead C–C axis.

Table S9. Selected S_1-state data for **Py**, **3Py**, **Phe**, and **3Phe** at their S_1-optimized structures, calculated at the B3LYP-D3(BJ)/6-31G(d,p) (PCM, $CHCl_3$) level.

compound	energy/eV	λ/nm	*f* [a]	emission assignment [b]	
**Py**	3.61	343	0.0008	H–1←L(44.7%)	H←L+1(55.0%)
**3Py**	2.78	446	0.6372	H←L(98.6%)	
**Phe**	3.76	329	0.0039	H–1←L(37.2%)	H←L+1(62.0%)
**3Phe**	3.59	345	0.5232	H←L(81.4%)	

[a] Oscillator strength. [b] H: HOMO, L: LUMO. Only transitions with contributions >10% are listed.

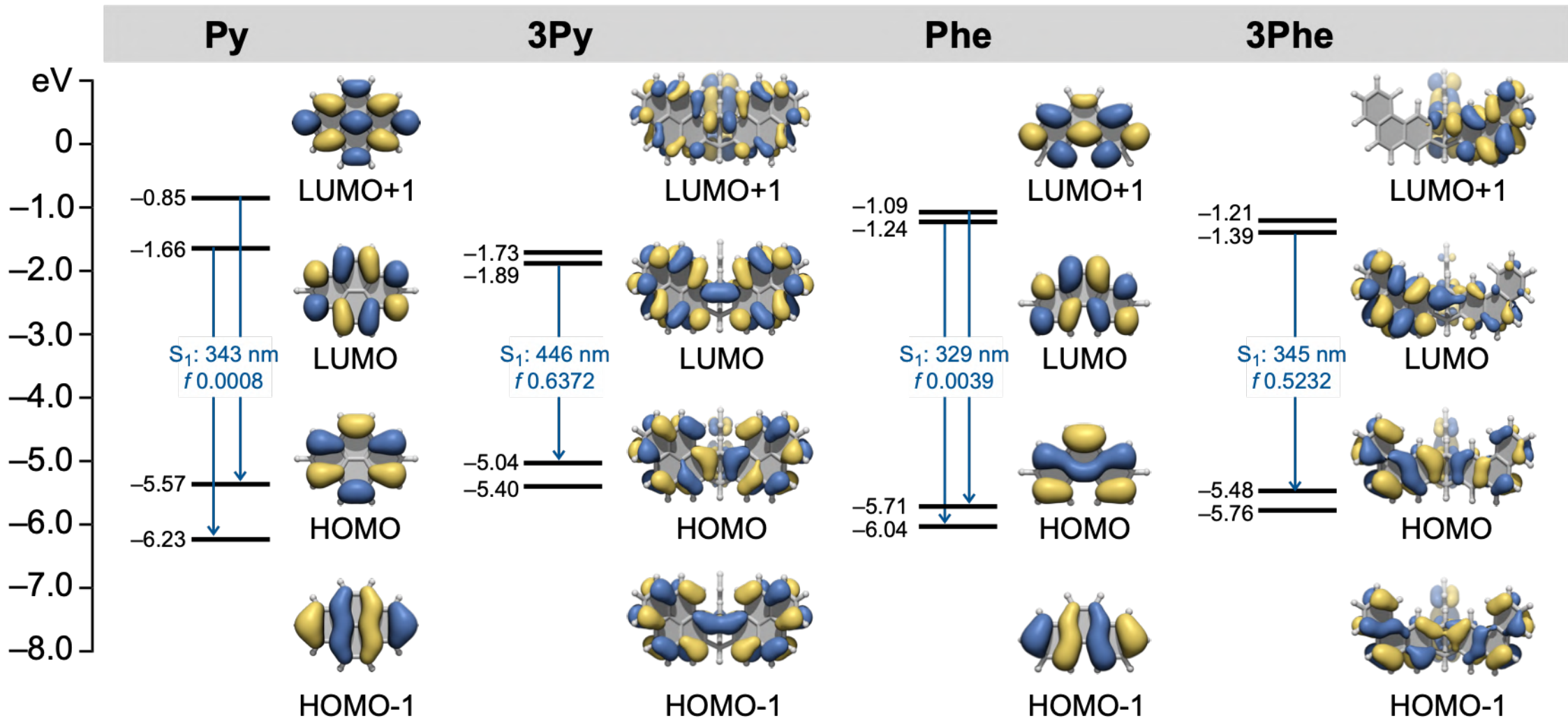

Figure S15. Frontier molecular orbital energy diagrams and selected electronic transitions of **Py**, **3Py**, **Phe**, and **3Phe** at their S_1-optimized structures, calculated at the B3LYP-D3(BJ)/6-31G(d,p) (PCM, $CHCl_3$) level. The corresponding HOMO−1, HOMO, LUMO, and LUMO+1 orbitals are also shown, together with the emission wavelengths and oscillator strengths (*f*) of the S_1 states.

Table S10. Selected S_1-state data for **Py**, **3Py**, **Phe**, and **3Phe** obtained from single-point TD-DFT calculations at the CAM-B3LYP-D3(BJ)/6-31G(d,p)//B3LYP-D3(BJ)/6-31G(d,p) and PBE0/6-31G(d,p)//B3LYP-D3(BJ)/6-31G(d,p) levels, with PCM ($CHCl_3$) applied throughout.

compound	energy/eV	λ/nm	f [a]	emission assignment [b]
CAM-B3LYP-D3(BJ)/6-31G(d,p)//B3LYP-D3(BJ)/6-31G(d,p) (PCM, $CHCl_3$)				
**Py**	3.79	327	0.0005	H–1←L(45.1%) H←L+1(53.2%)
**3Py**	3.33	372	0.9678	H←L(81.5%)
**Phe**	4.02	308	0.0022	H–1←L(39.2%) H←L+1(56.7%)
**3Phe**	4.01	309	0.2085	H–5←L(14.5%) H←L(29.5%) H←L+2(20.5%)
PBE0/6-31G(d,p)//B3LYP-D3(BJ)/6-31G(d,p) (PCM, $CHCl_3$)				
**Py**	3.68	337	0.0009	H–1←L(44.2%) H←L+1(55.4%)
**3Py**	2.94	422	0.6102	H←L(98.4%)
**Phe**	3.85	322	0.0031	H–1←L(37.8%) H←L+1(61.1%)
**3Phe**	3.73	332	0.3896	H←L(67.1%) H←L+2(12.6%)

[a] Oscillator strength. [b] H: HOMO, L: LUMO. Only transitions with contributions >10% are listed.

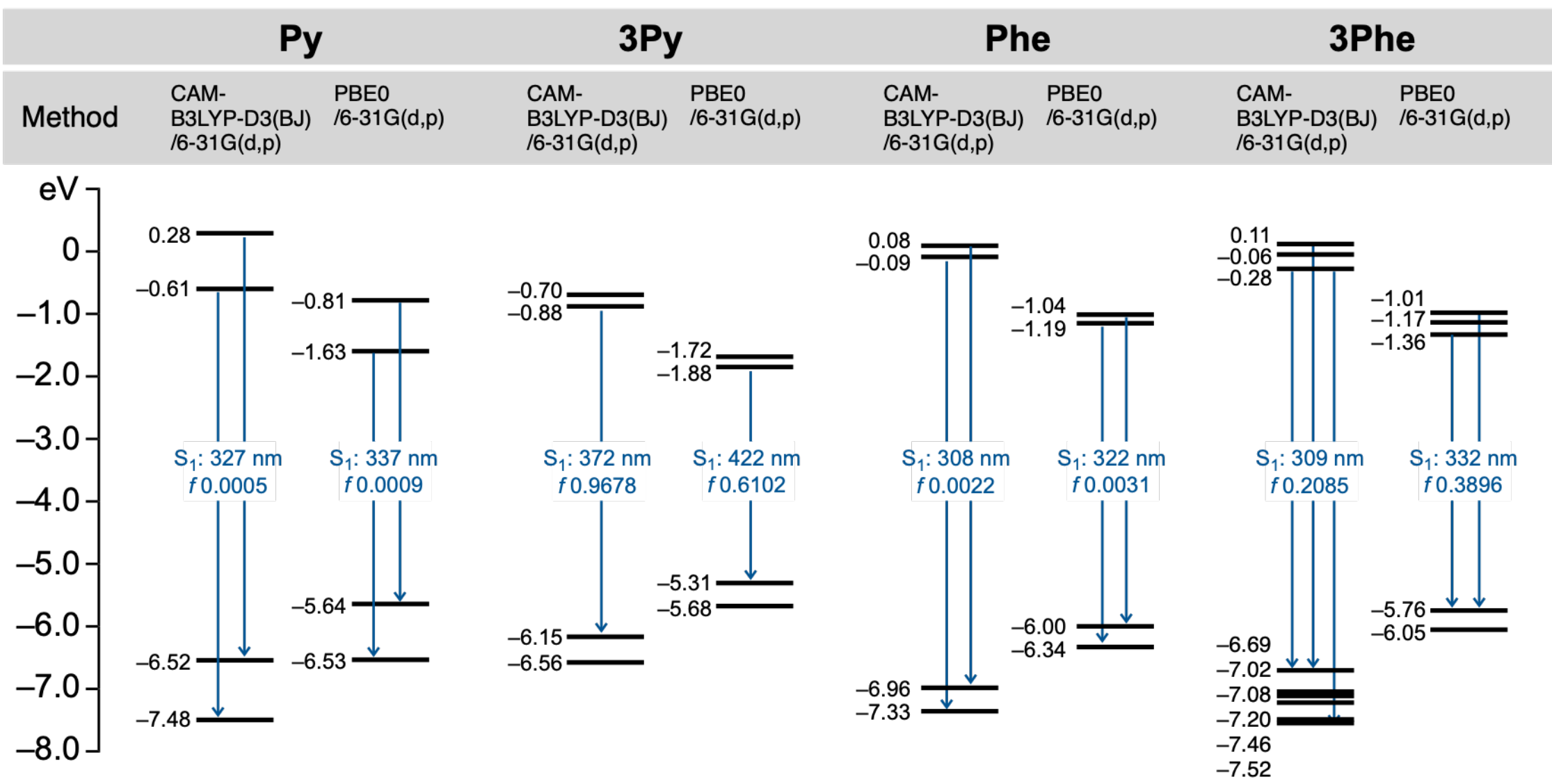

Figure S16. Frontier molecular orbital energy diagrams and selected electronic transitions of **Py**, **3Py**, **Phe**, and **3Phe** at their S_1-optimized structures, calculated at the CAM-B3LYP-D3(BJ)/6-31G(d,p)//B3LYP-D3(BJ)/6-31G(d,p) and PBE0/6-31G(d,p)//B3LYP-D3(BJ)/6-31G(d,p) levels, with PCM ($CHCl_3$) applied throughout. The emission wavelengths and oscillator strengths (f) of the S_1 states are also shown.

6. Electrochemical measurements

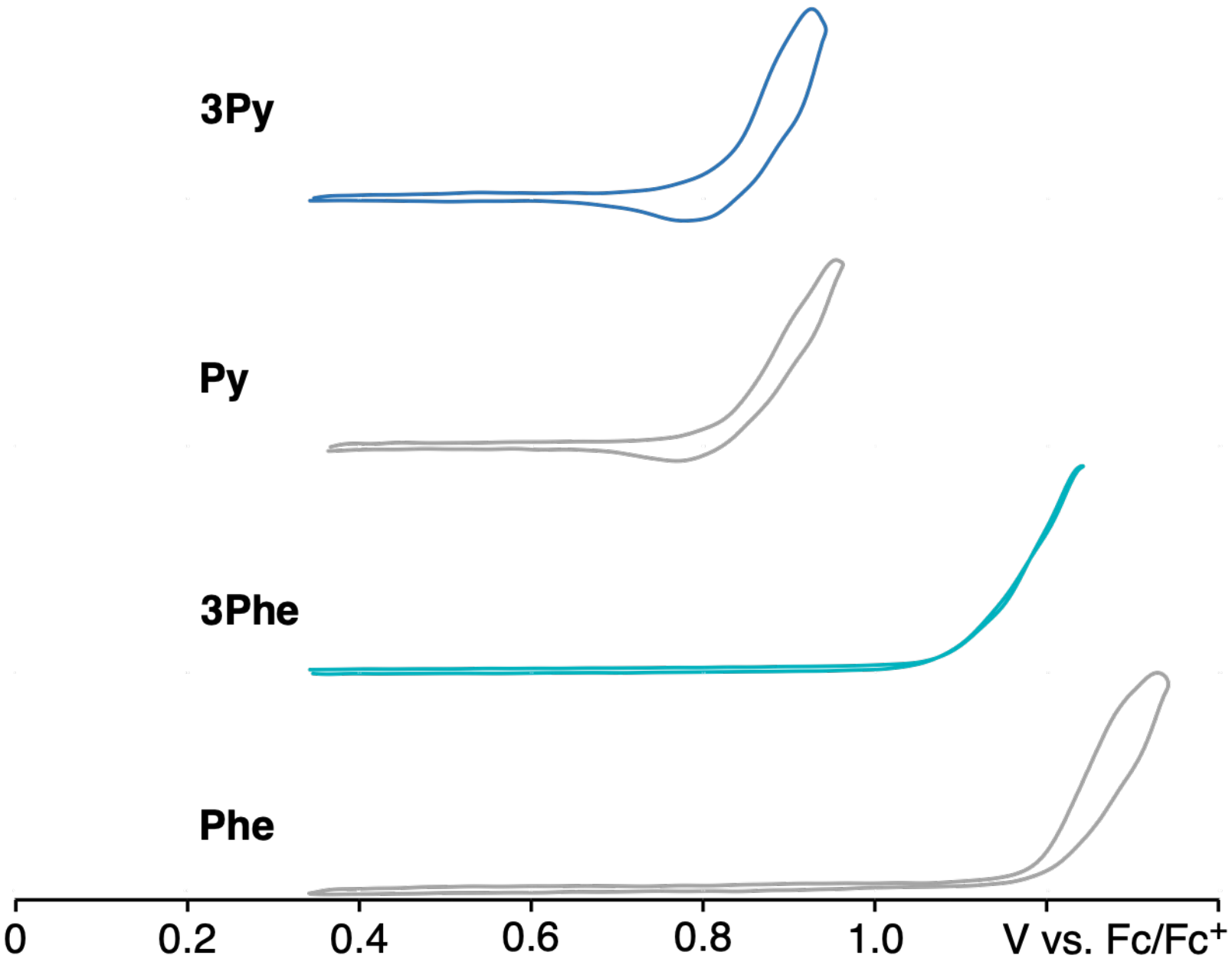

Figure S17. Cyclic voltammograms of **3Py**, **Py**, **3Phe**, and **Phe** at room temperature. Conditions: Pt disk working electrode, Pt counter electrode, Ag/AgCl reference electrode, and 0.10 mol L^{-1} Bu_4NPF_6 as the supporting electrolyte in CH_3CN/benzene (1/1, v/v).

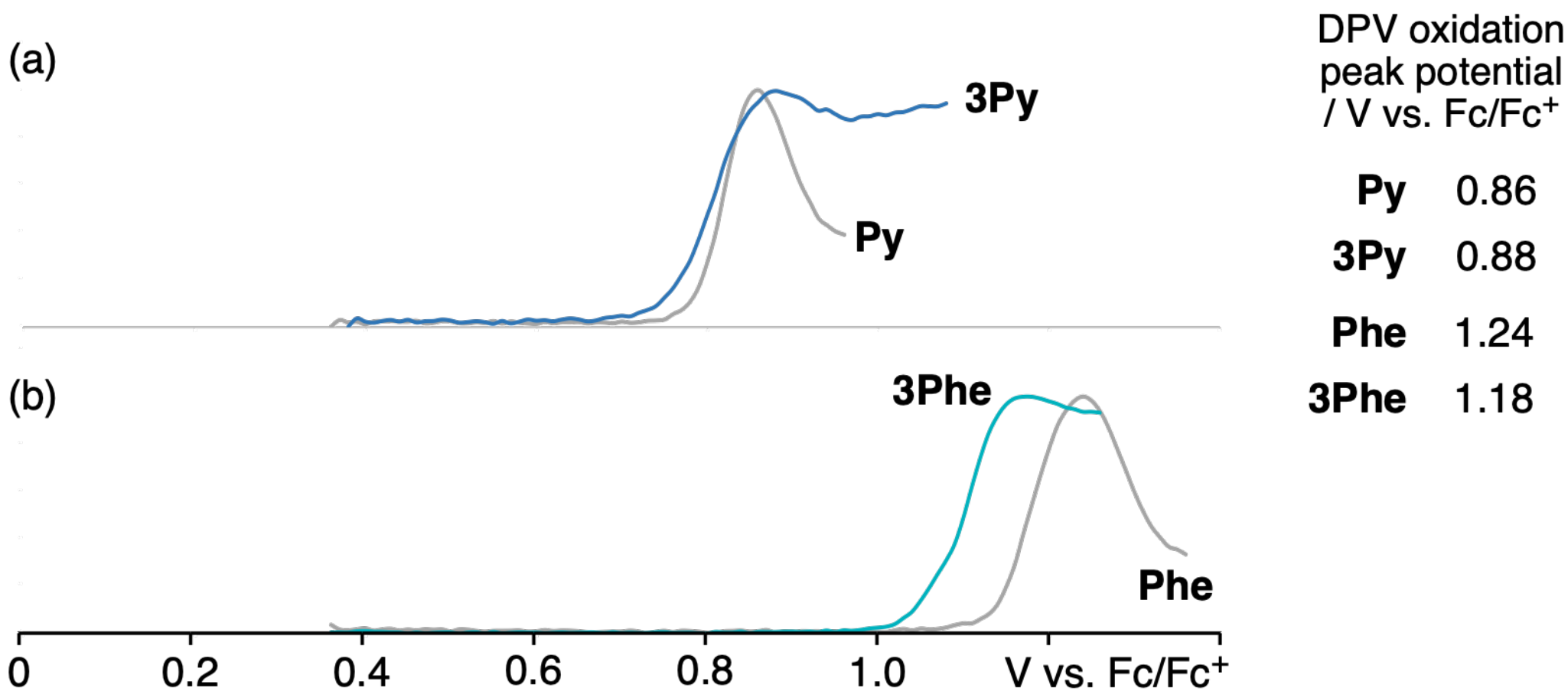

Figure S18. Differential pulse voltammograms of (a) **Py** and **3Py** and (b) **Phe** and **3Phe** at room temperature. Conditions: Pt disk working electrode, Pt counter electrode, Ag/AgCl reference electrode, and 0.10 mol L^{-1} Bu_4NPF_6 as the supporting electrolyte in CH_3CN/benzene (1/1, v/v).

7. NMR spectra

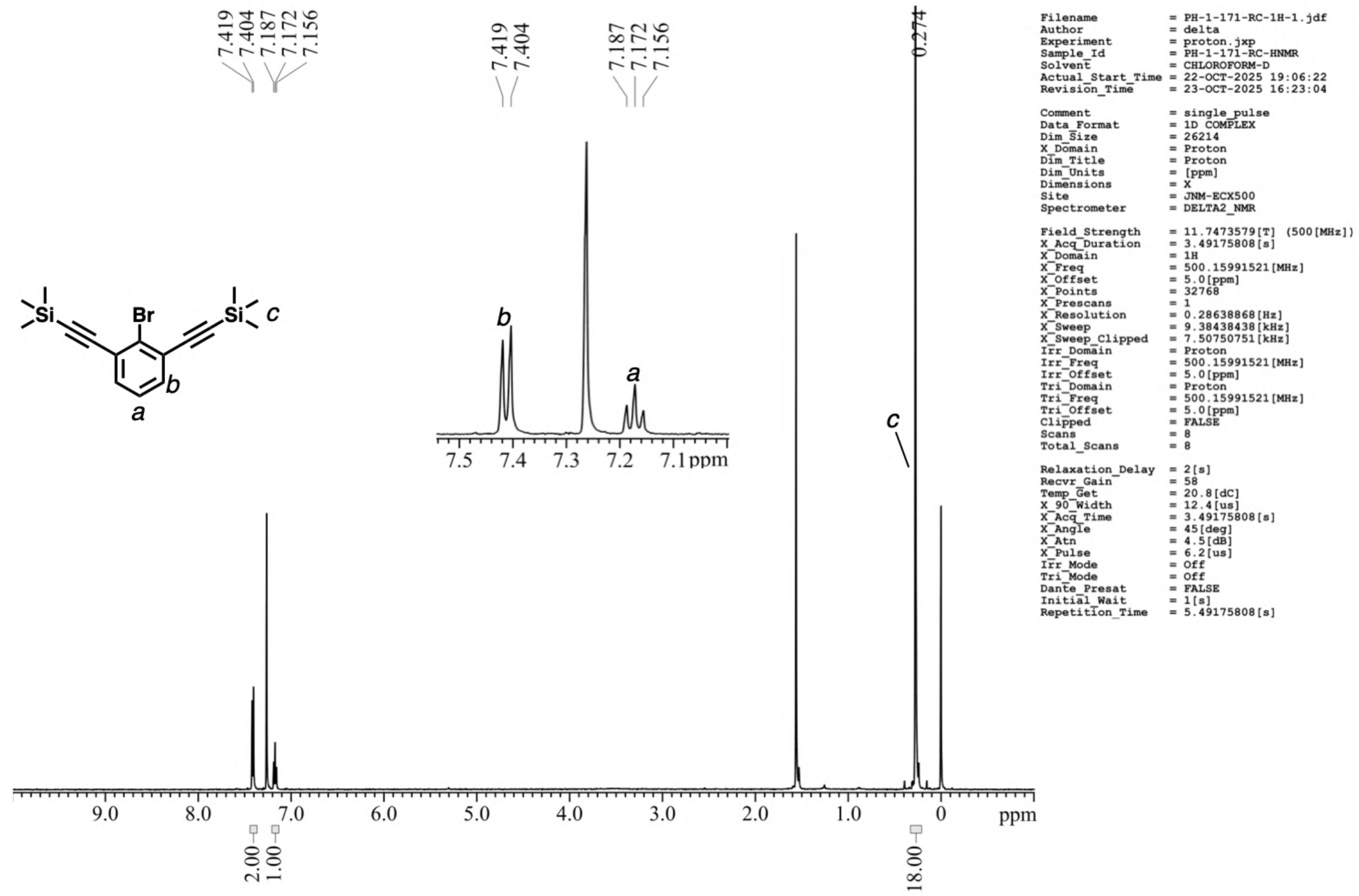

Figure S19. ^{1}H NMR spectrum (500 MHz, $CDCl_3$, 298 K) of **A**.

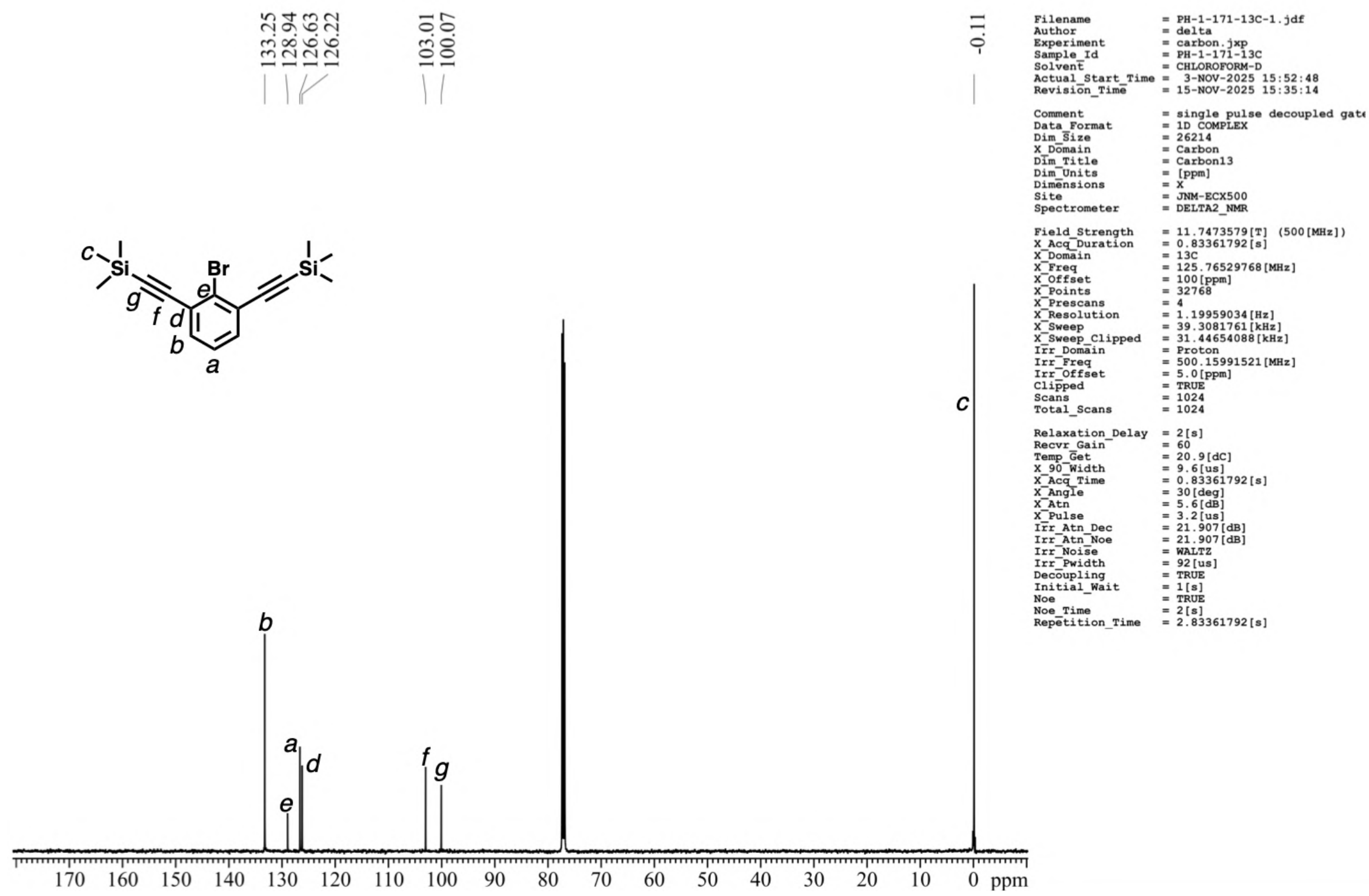

Figure S20. ^{13}C NMR spectrum (125 MHz, $CDCl_3$, 298 K) of **A**.

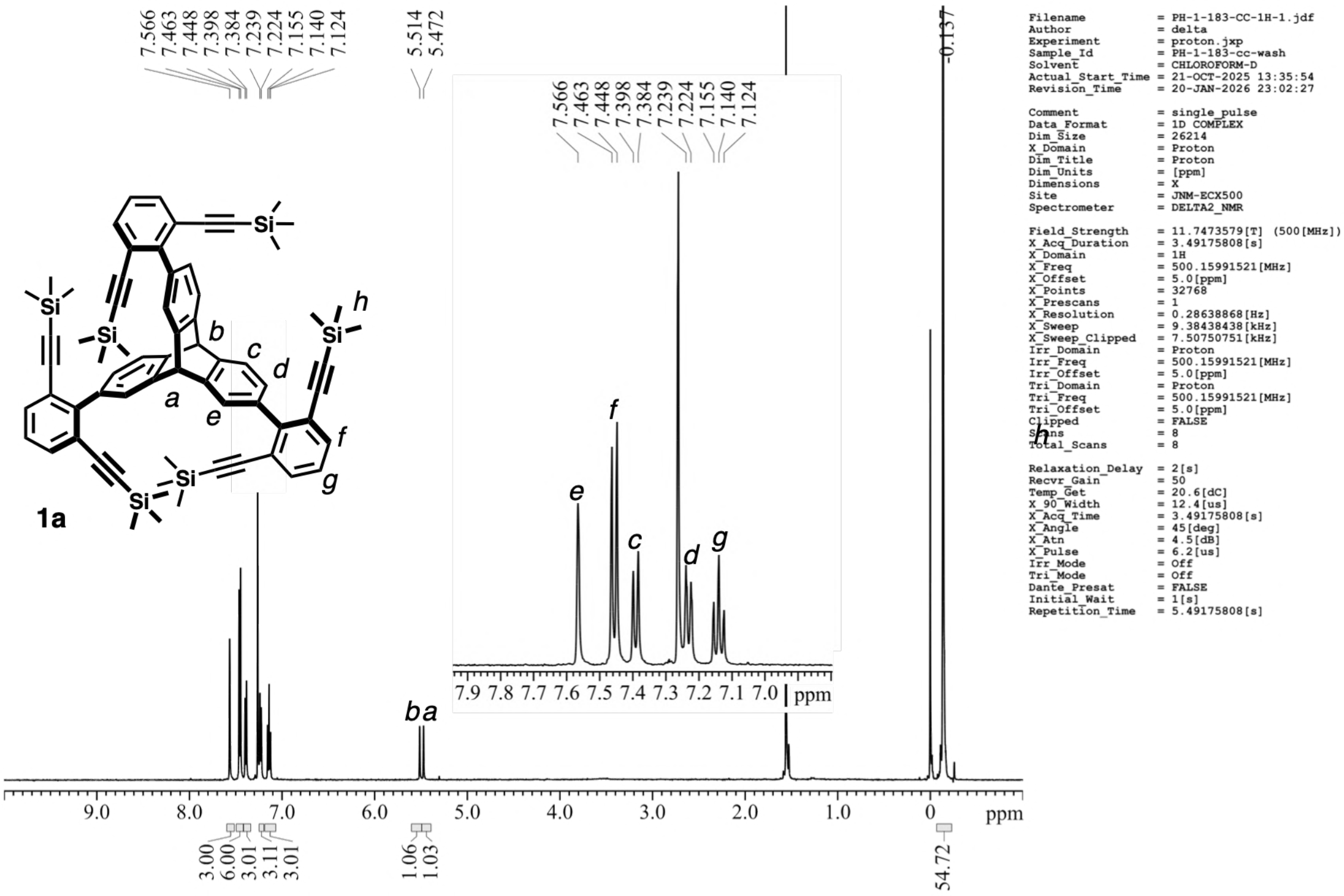

Figure S21. ^{1}H NMR spectrum (500 MHz, $CDCl_3$, 298 K) of **1a**.

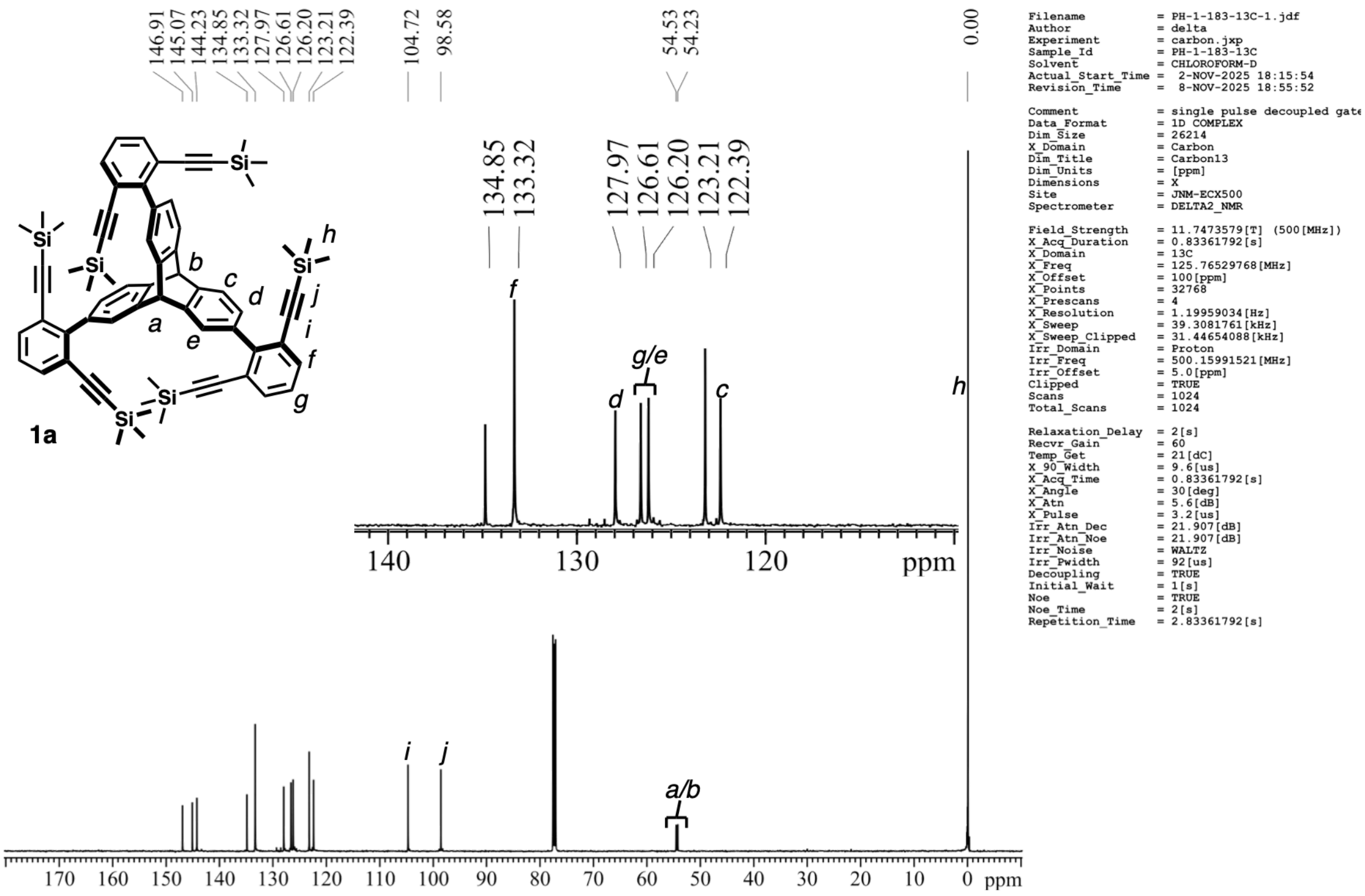

Figure S22. ^{13}C NMR spectrum (125 MHz, $CDCl_3$, 298 K) of **1a**.

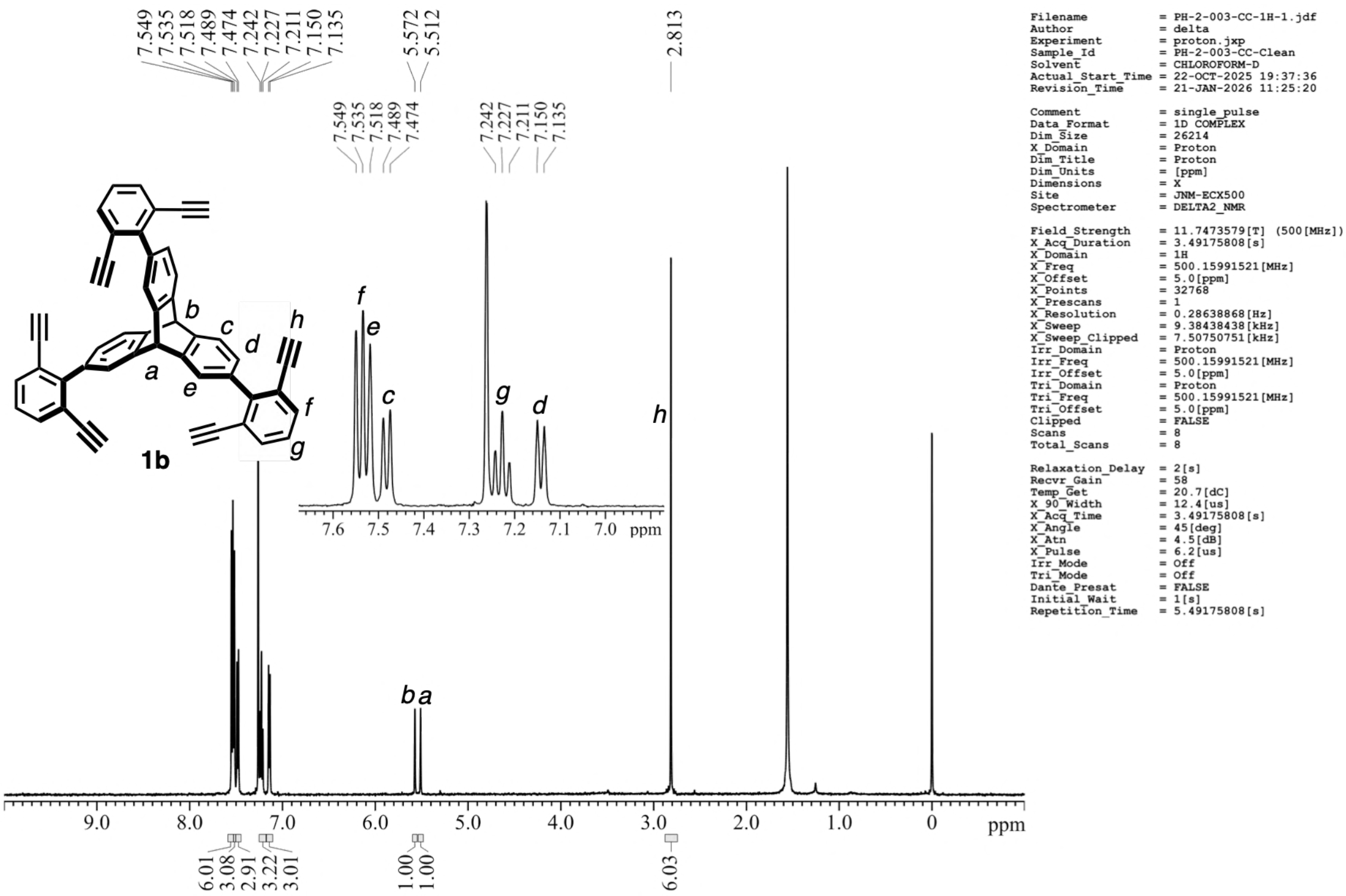

Figure S23. ^{1}H NMR spectrum (500 MHz, $CDCl_3$, 298 K) of **1b**.

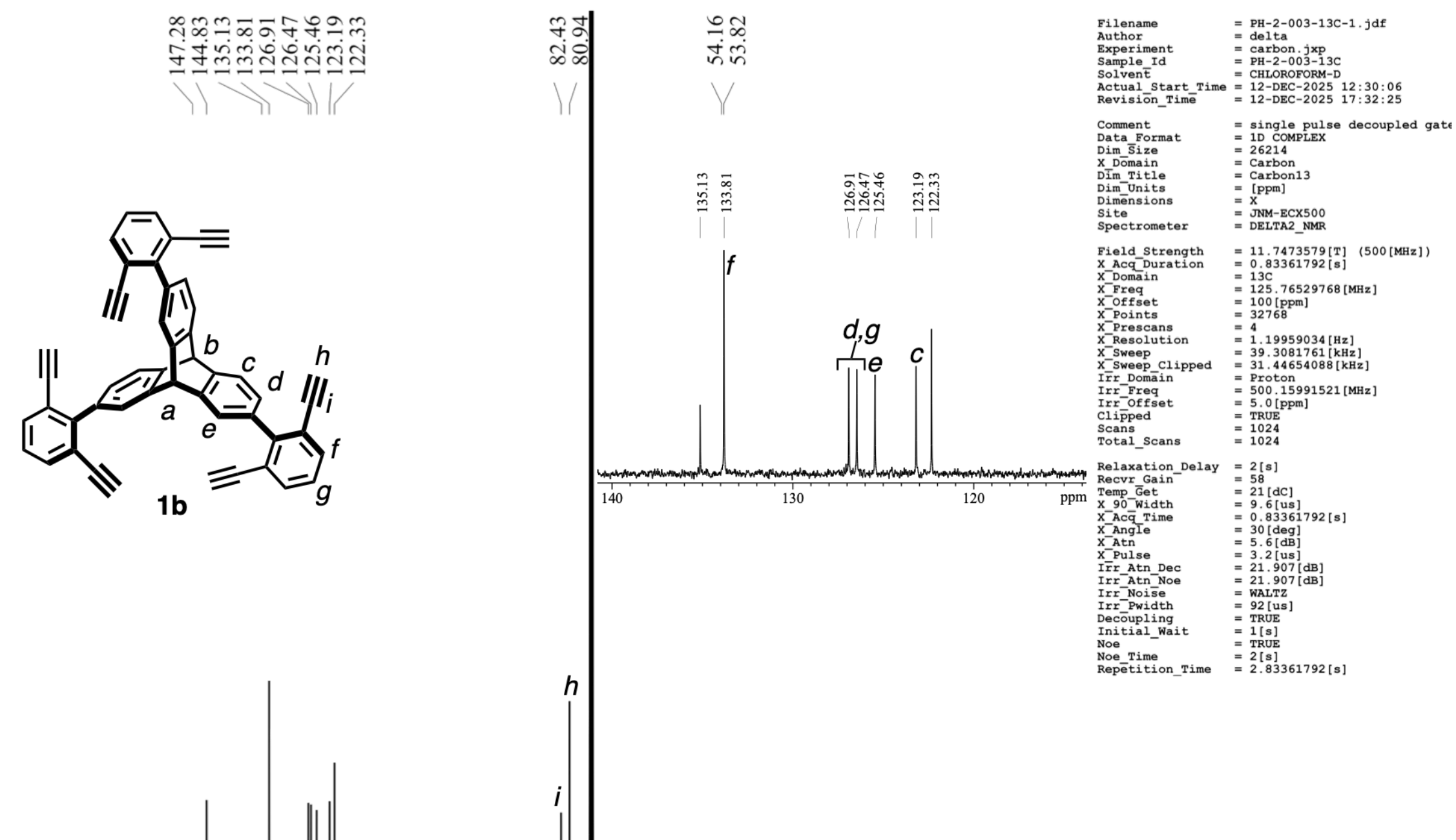

Figure S24. ^{13}C NMR spectrum (125 MHz, $CDCl_3$, 298 K) of **1b**.

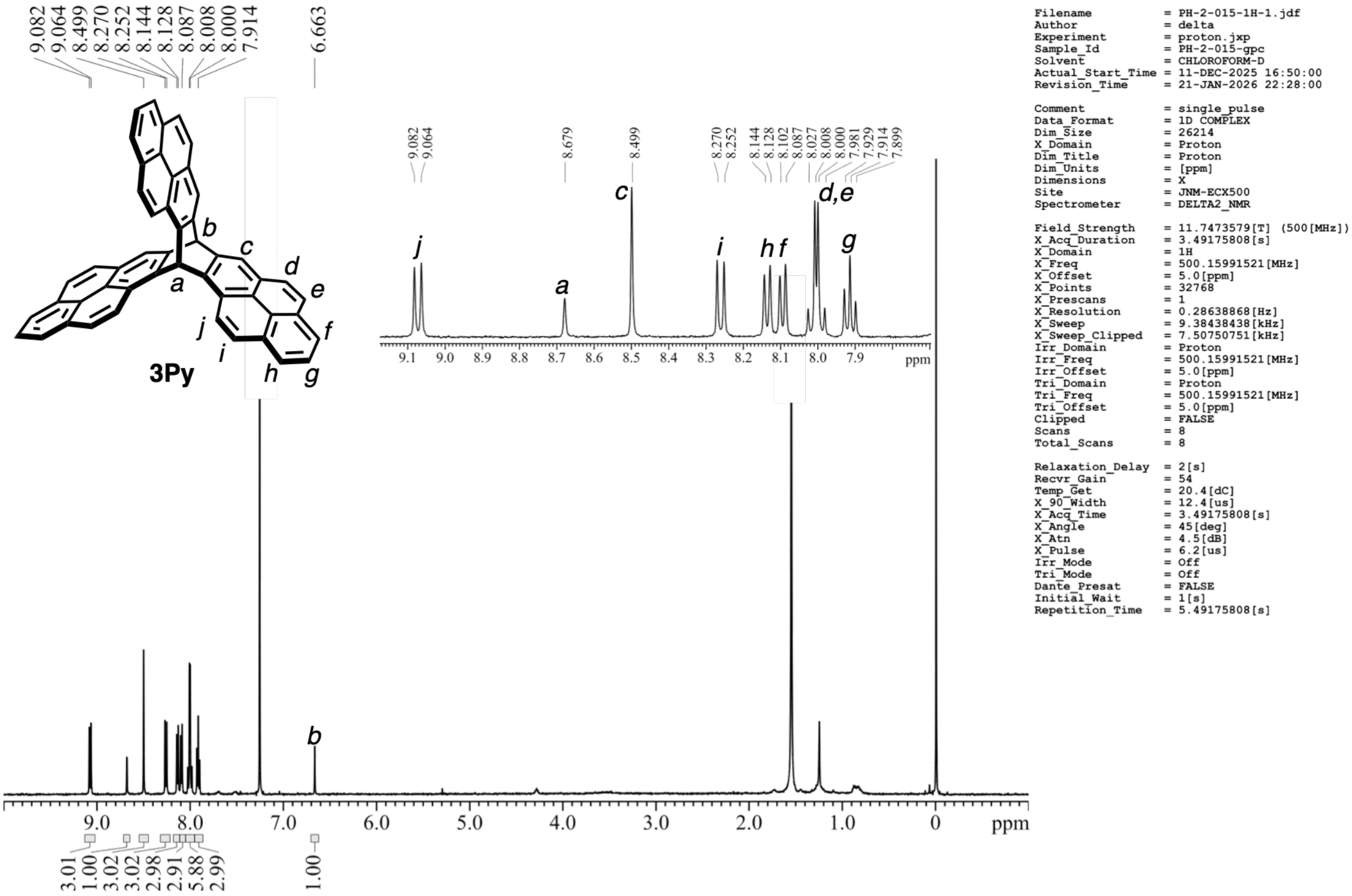

Figure S25. ^{1}H NMR spectrum (500 MHz, $CDCl_3$, 298 K) of **3Py**.

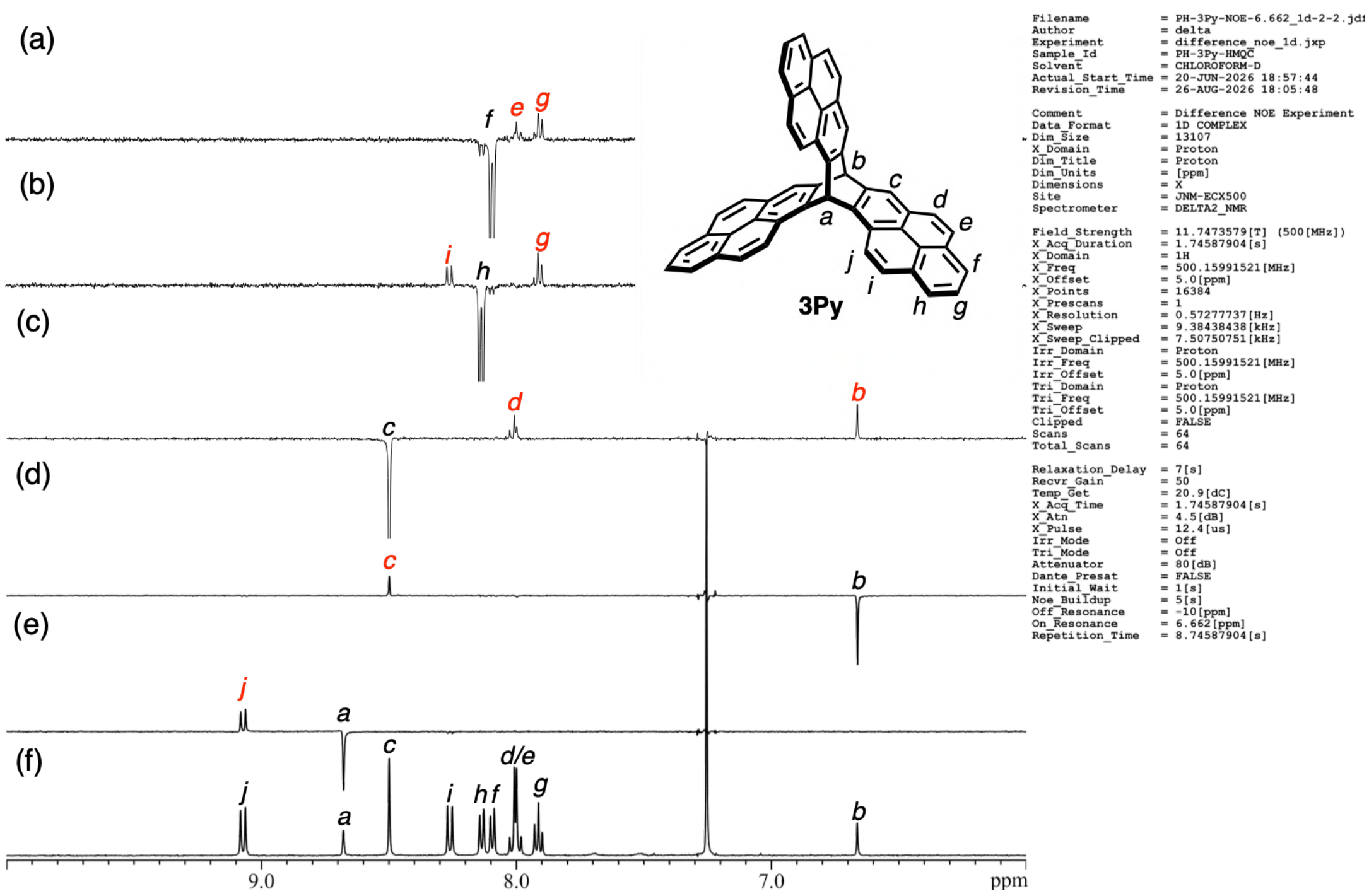

Figure S26. (a–e) 1D NOE spectra and (f) ^{1}H NMR spectrum (500 MHz, $CDCl_3$, 298 K) of **3Py**. Irradiation at (a) 8.10, (b) 8.14, (c) 8.50, (d) 6.66, and (e) 8.67 ppm.

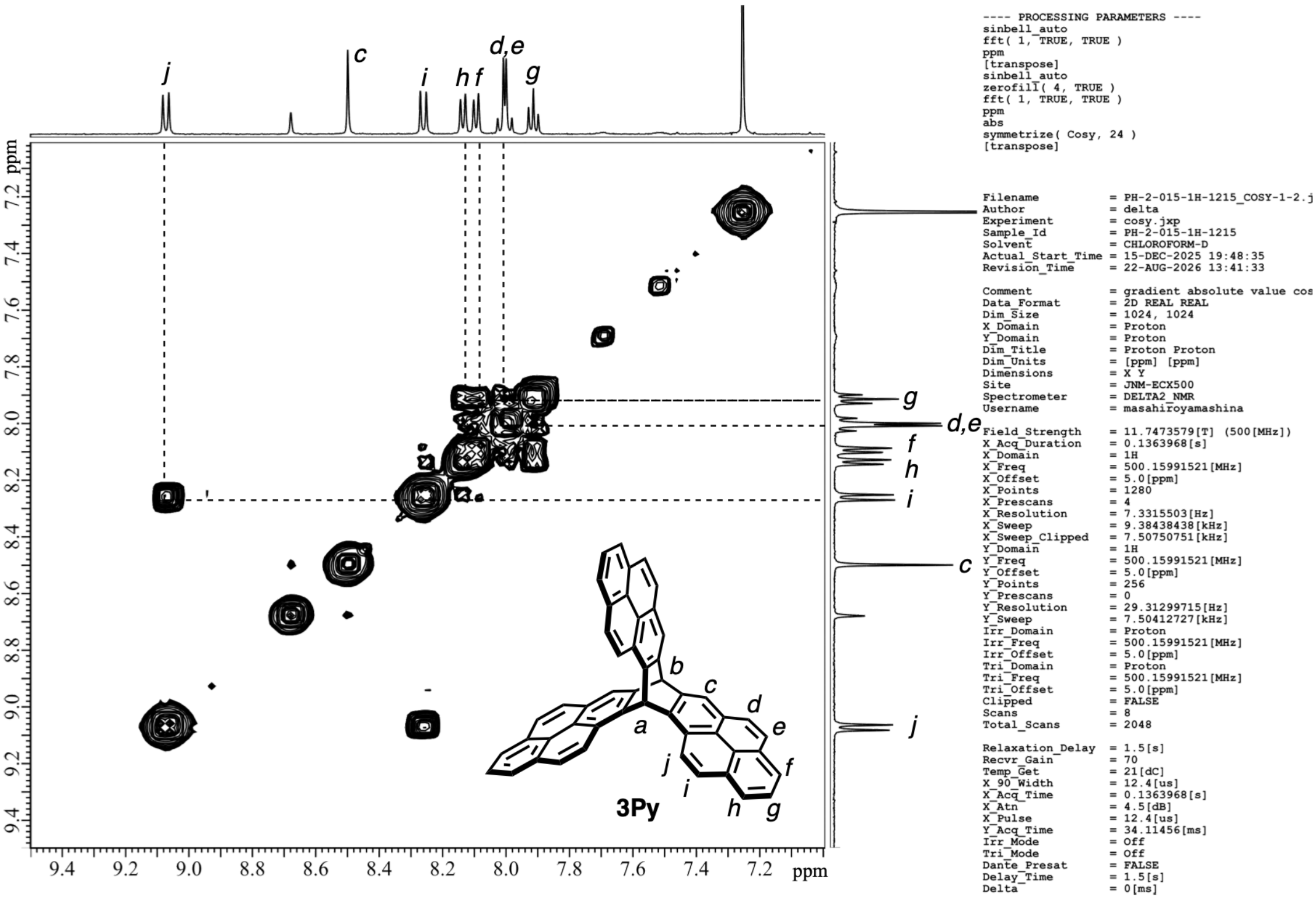

Figure S27. ^1H–^1H COSY spectrum (500 MHz, $CDCl_3$, 298 K) of **3Py**.

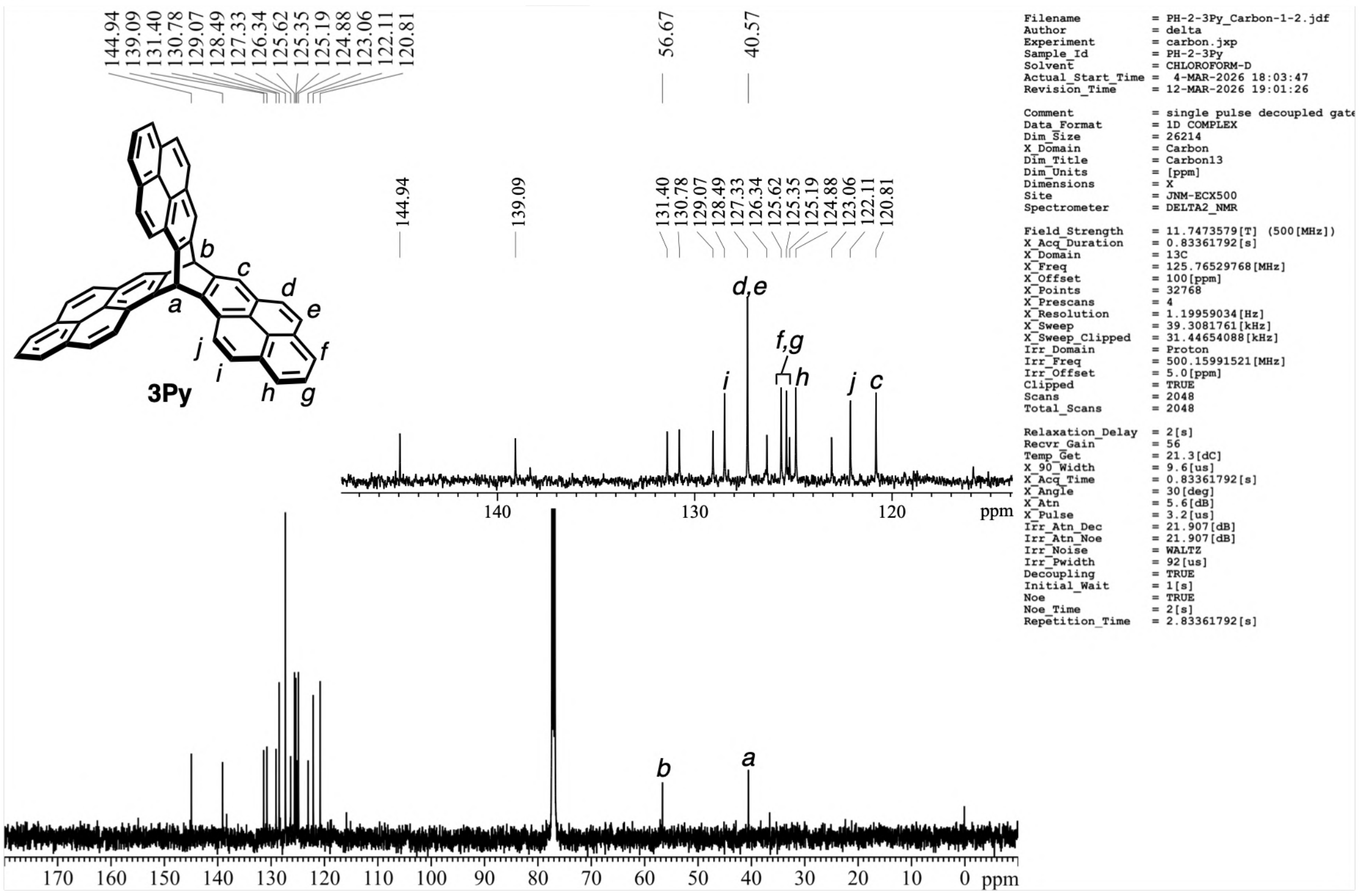

Figure S28. ^{13}C NMR spectrum (125 MHz, $CDCl_3$, 5.0 mM, 298 K) of **3Py**.

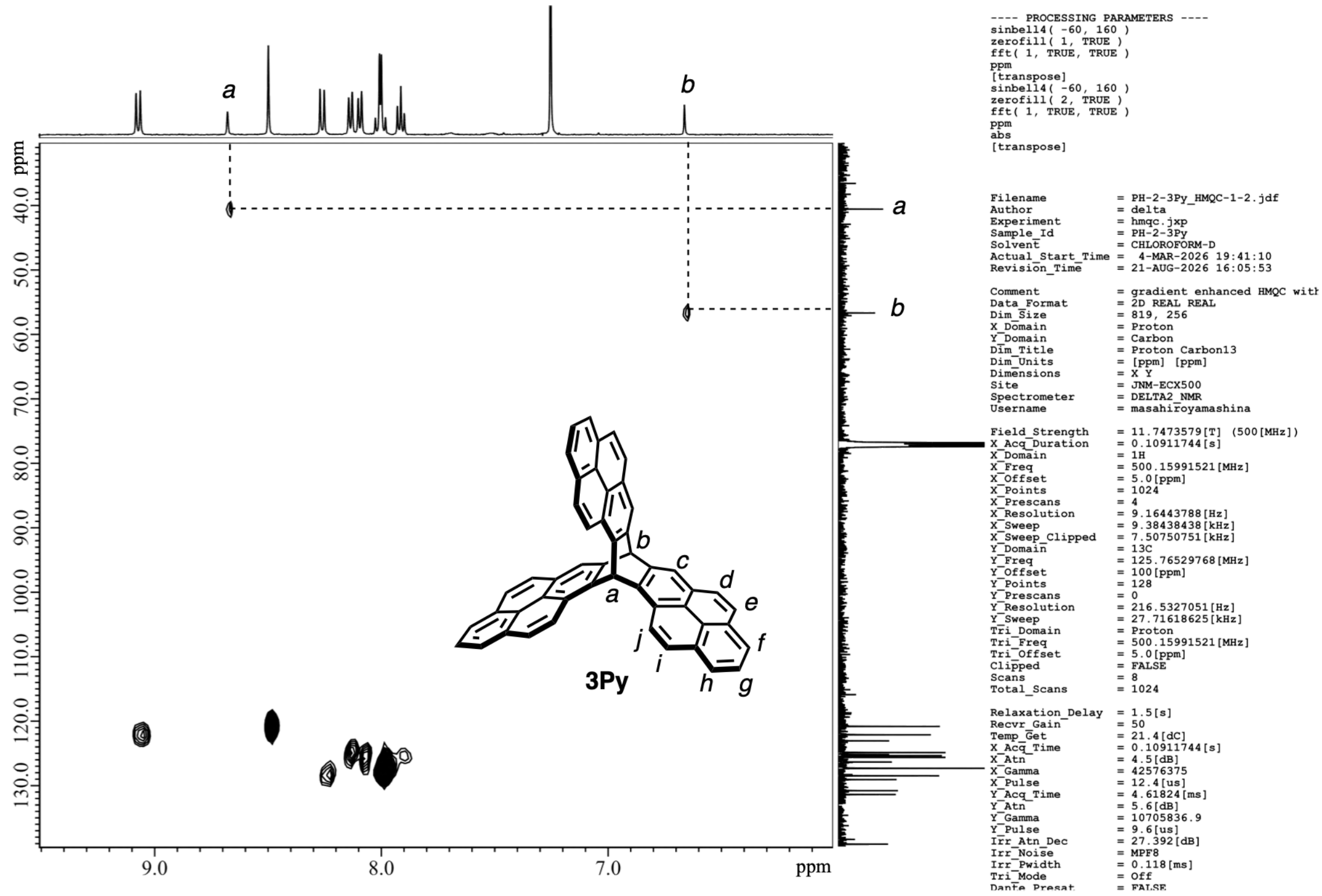

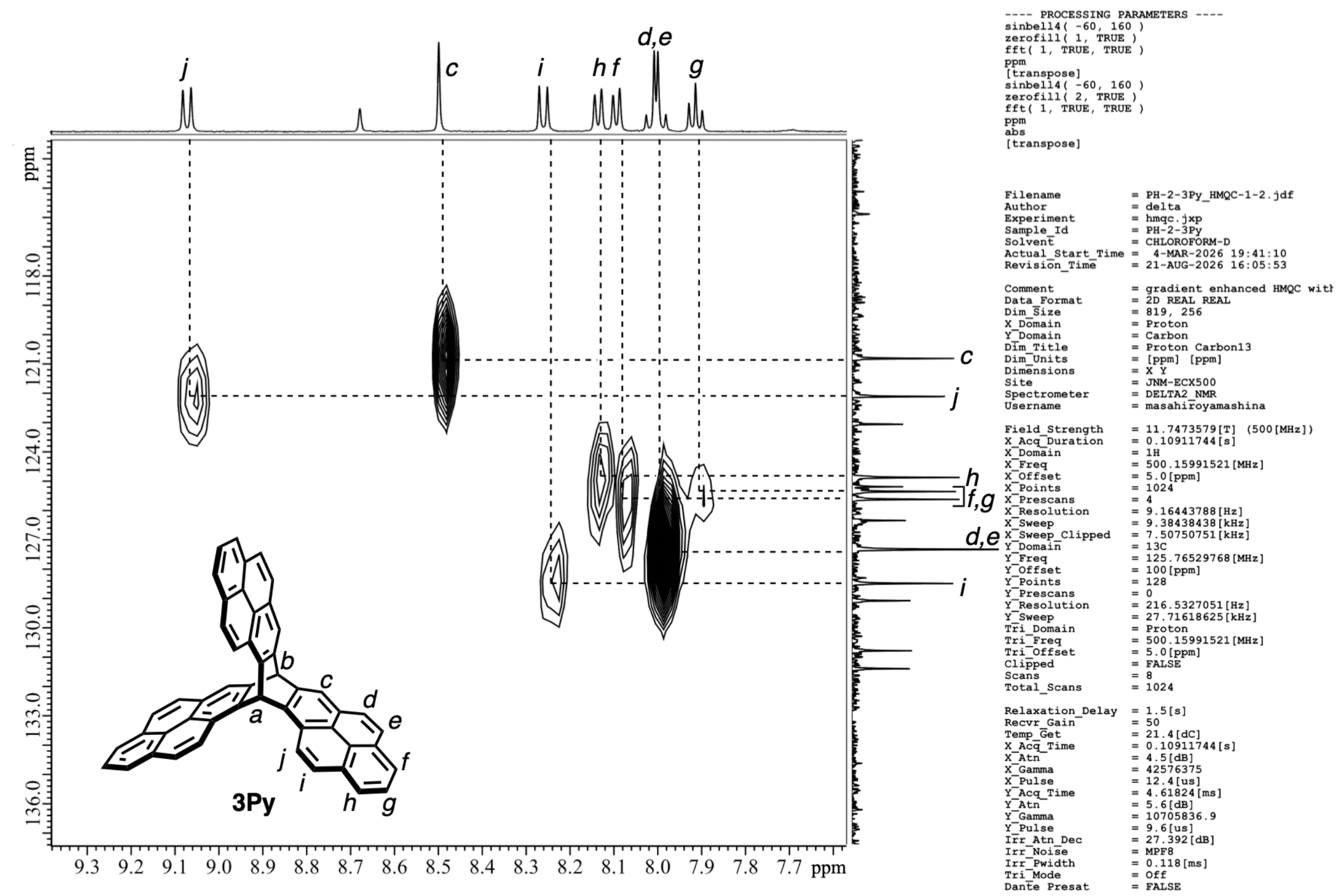

Figure S29. HMQC spectrum (500 MHz, $CDCl_3$, 5.0 mM, 298 K) of **3Py**. Top: 6.0–9.5 ppm region; bottom: 7.6–9.4 ppm region.

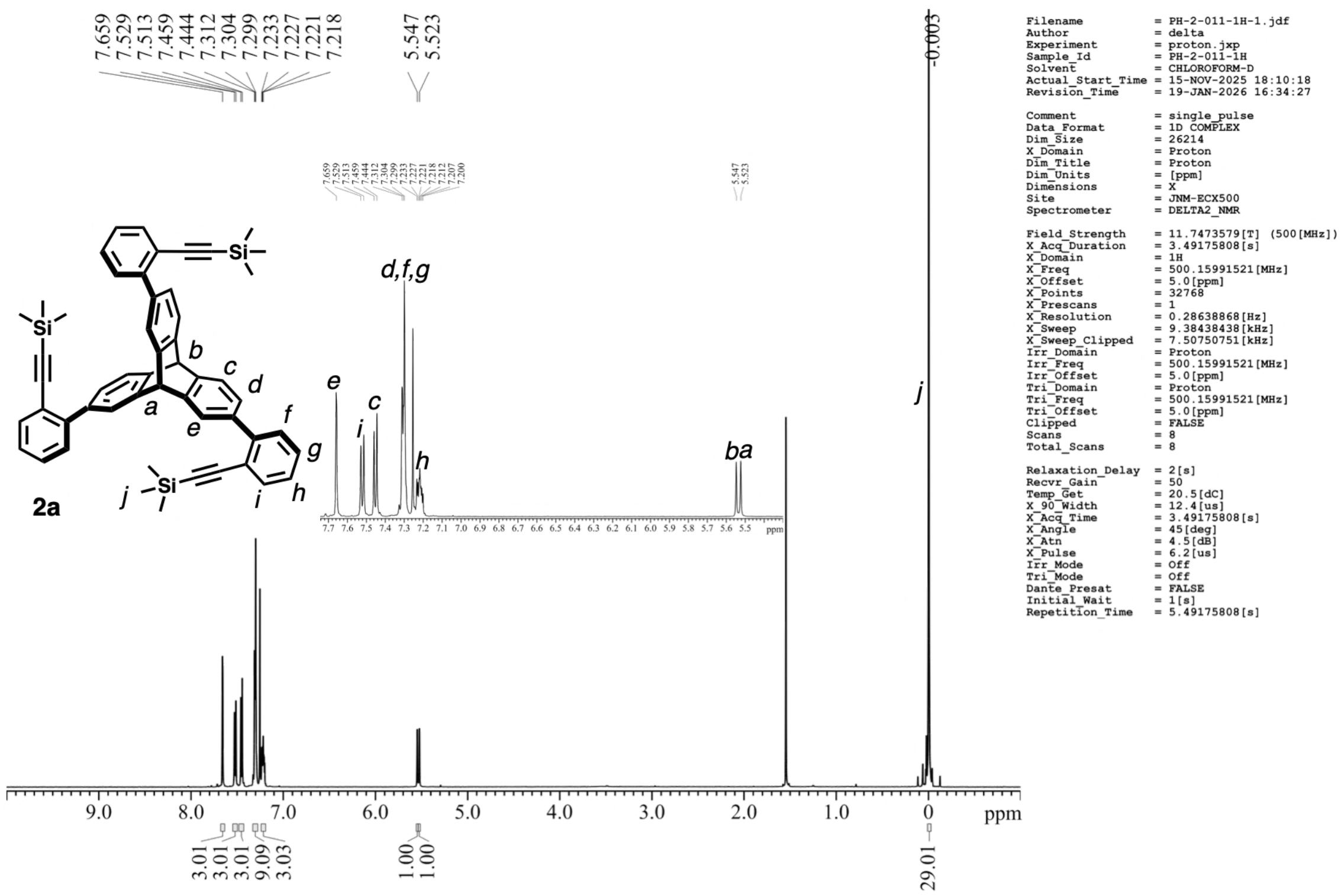

Figure S30. ^{1}H NMR spectrum (500 MHz, $CDCl_3$, 298 K) of **2a**.

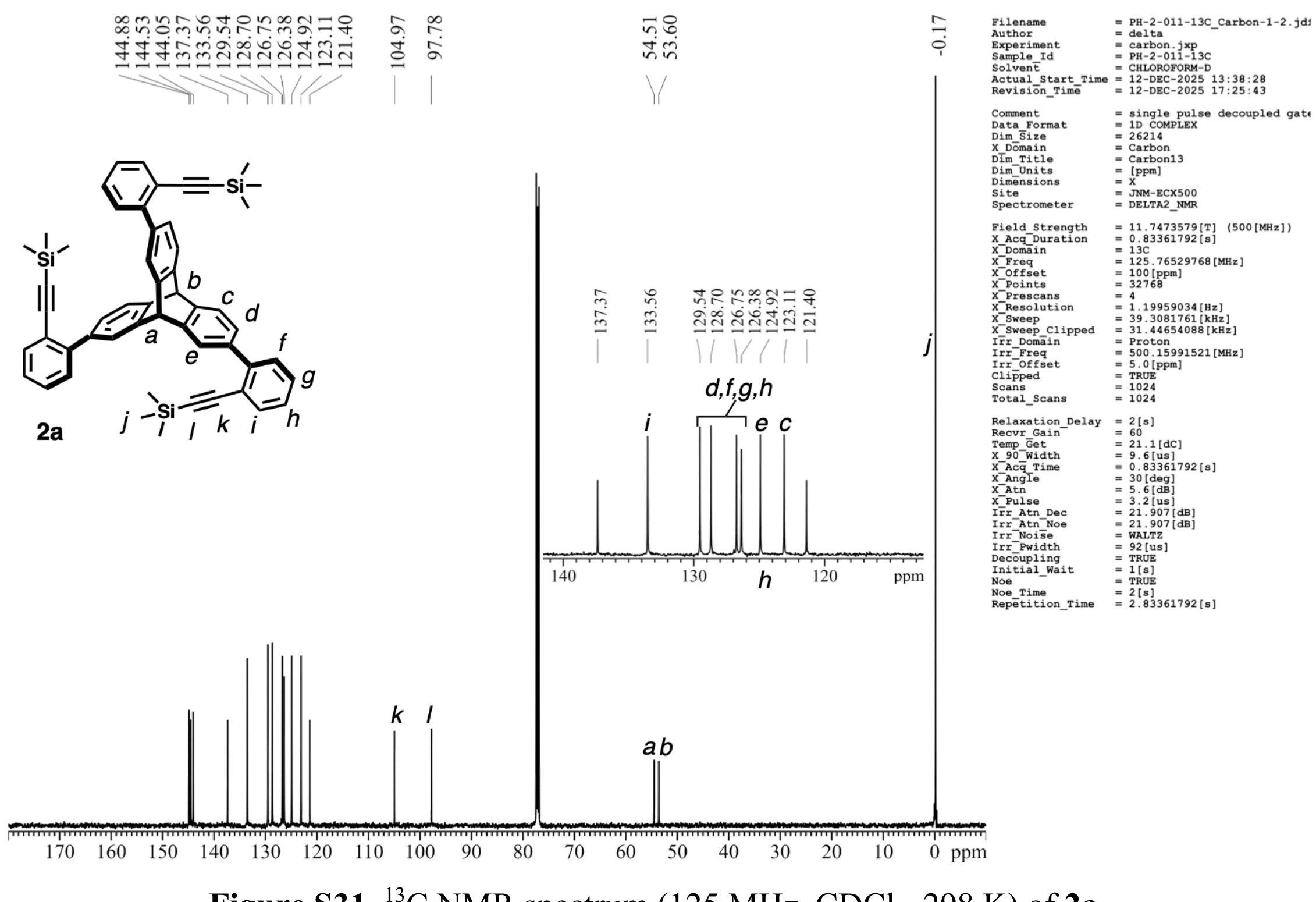

Figure S31. ^{13}C NMR spectrum (125 MHz, $CDCl_3$, 298 K) of **2a**.

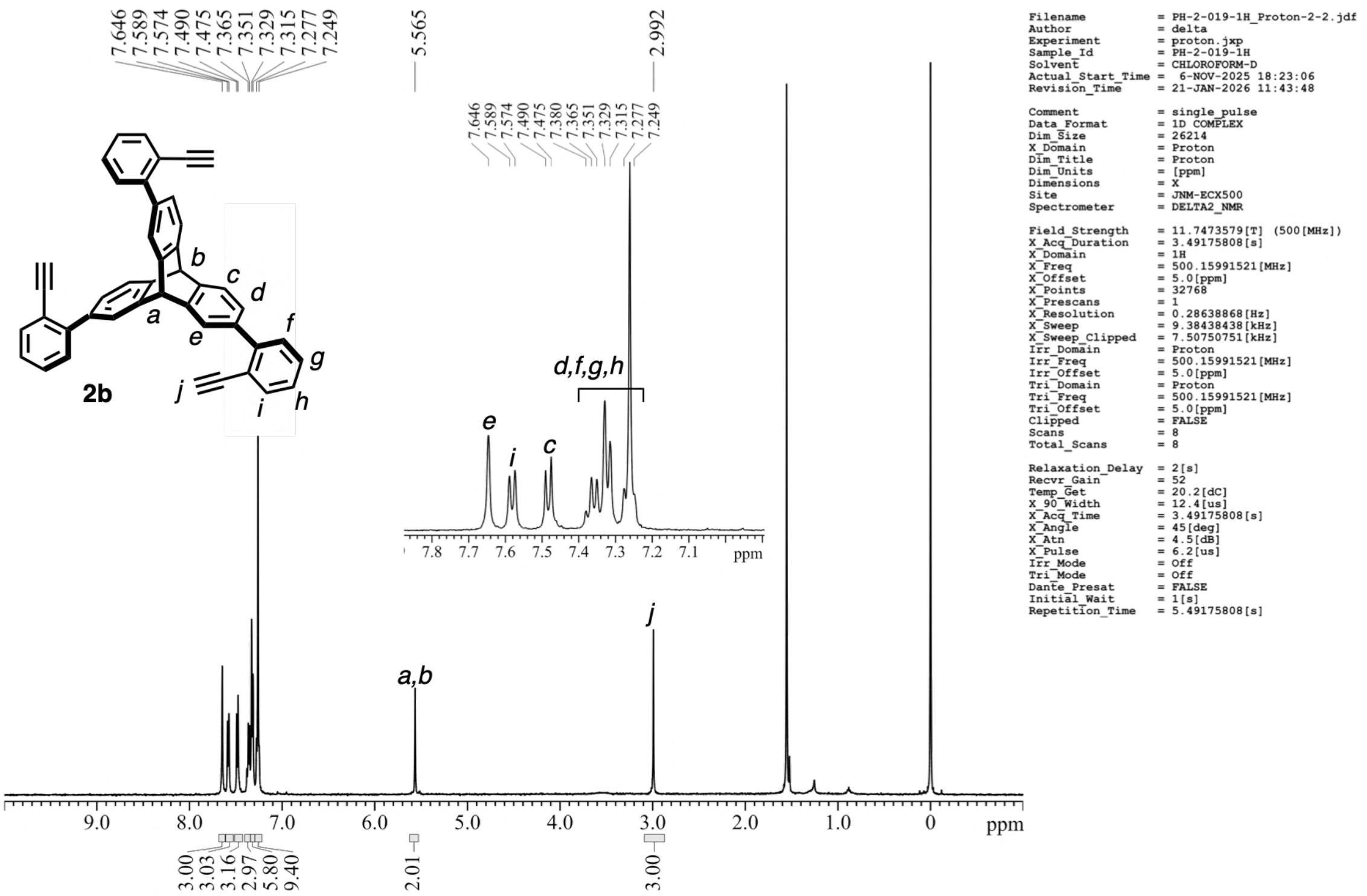

Figure S32. ^{1}H NMR spectrum (500 MHz, $CDCl_3$, 298 K) of **2b**.

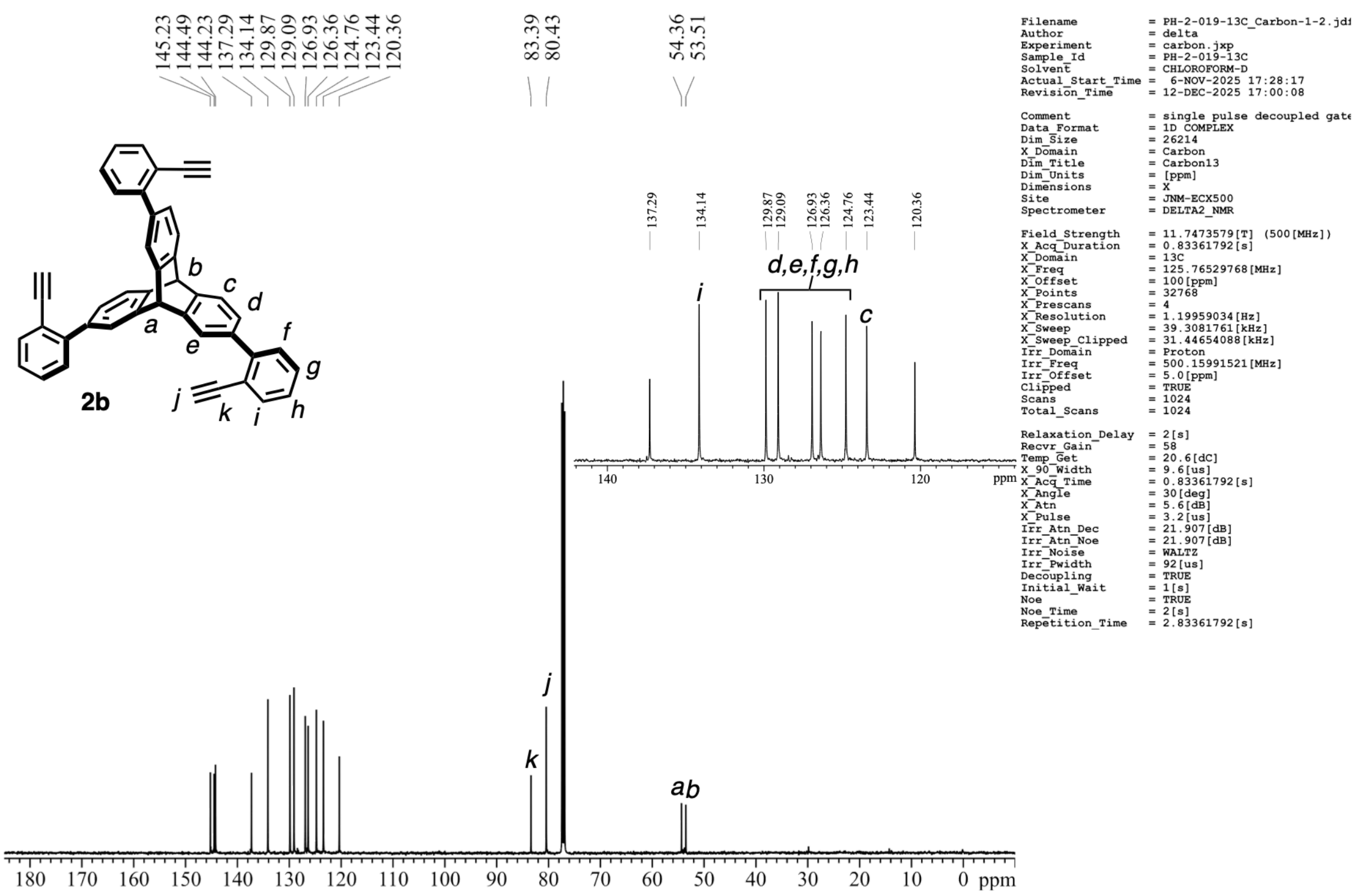

Figure S33. ^{13}C NMR spectrum (125 MHz, $CDCl_3$, 298 K) of **2b**.

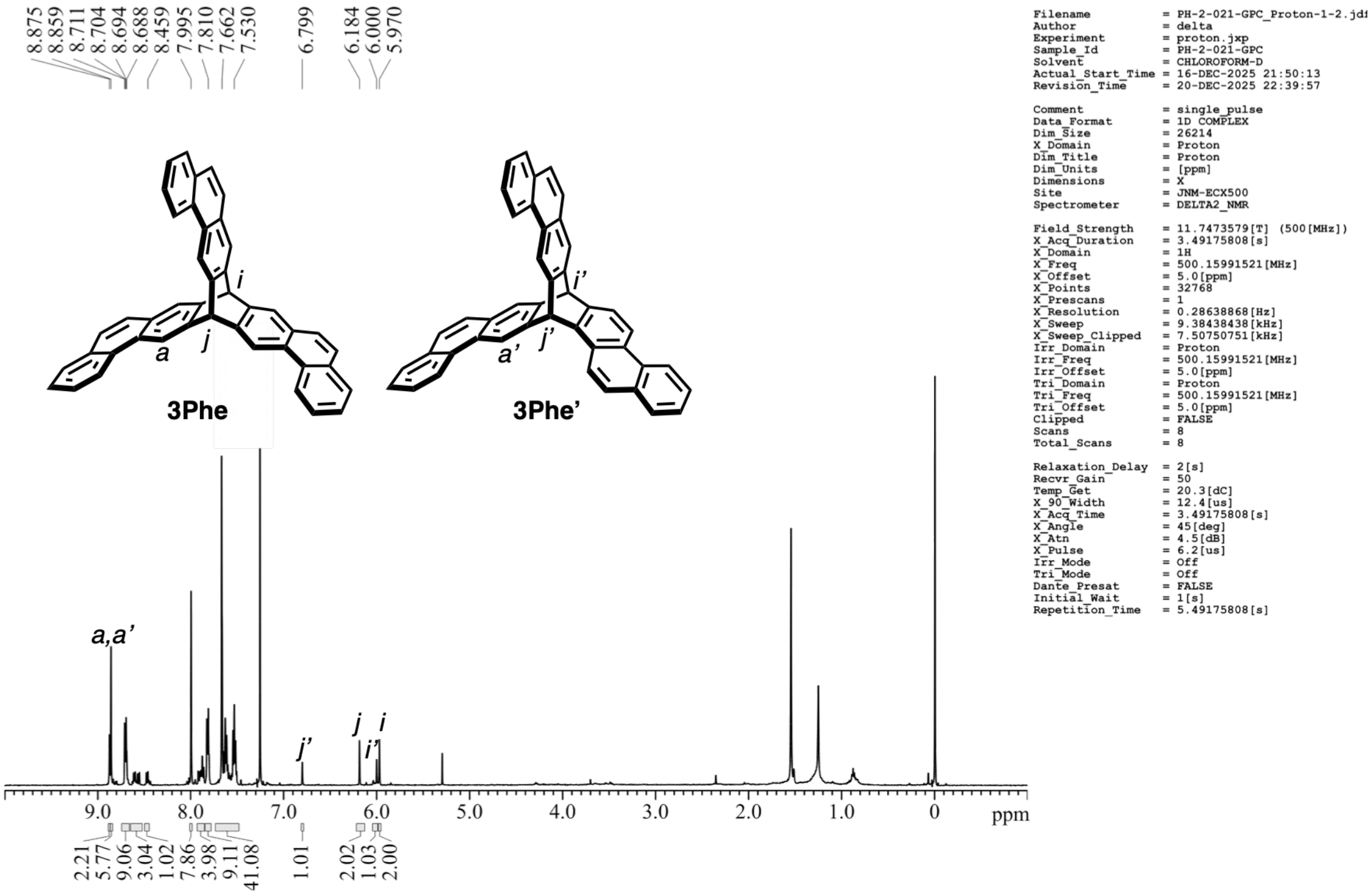

Figure S34. ^{1}H NMR spectrum (500 MHz, $CDCl_3$, 298 K) of a mixture of **3Phe/3Phe'**.

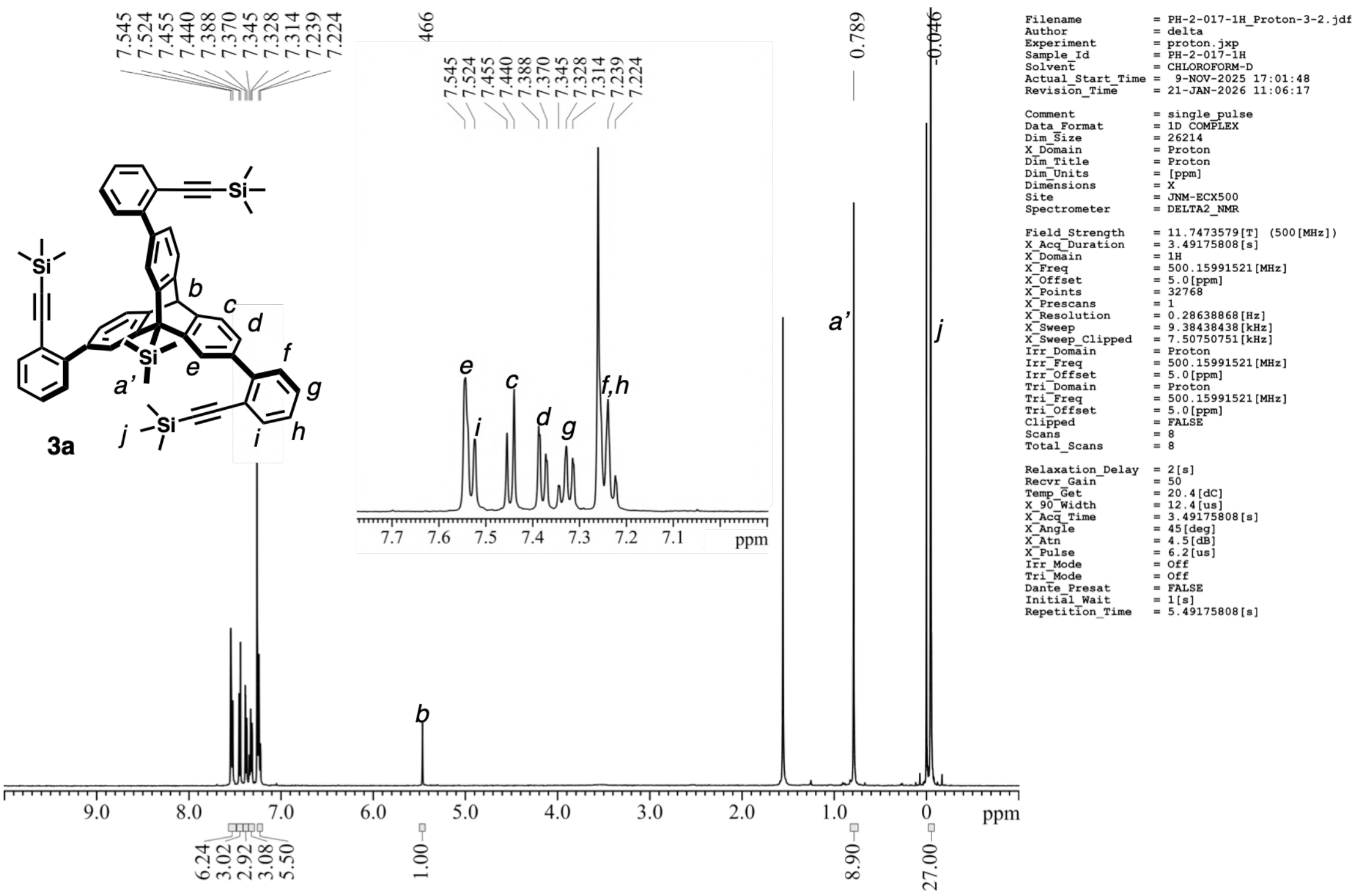

Figure S35. ^{1}H NMR spectrum (500 MHz, $CDCl_3$, 298 K) of **3a**.

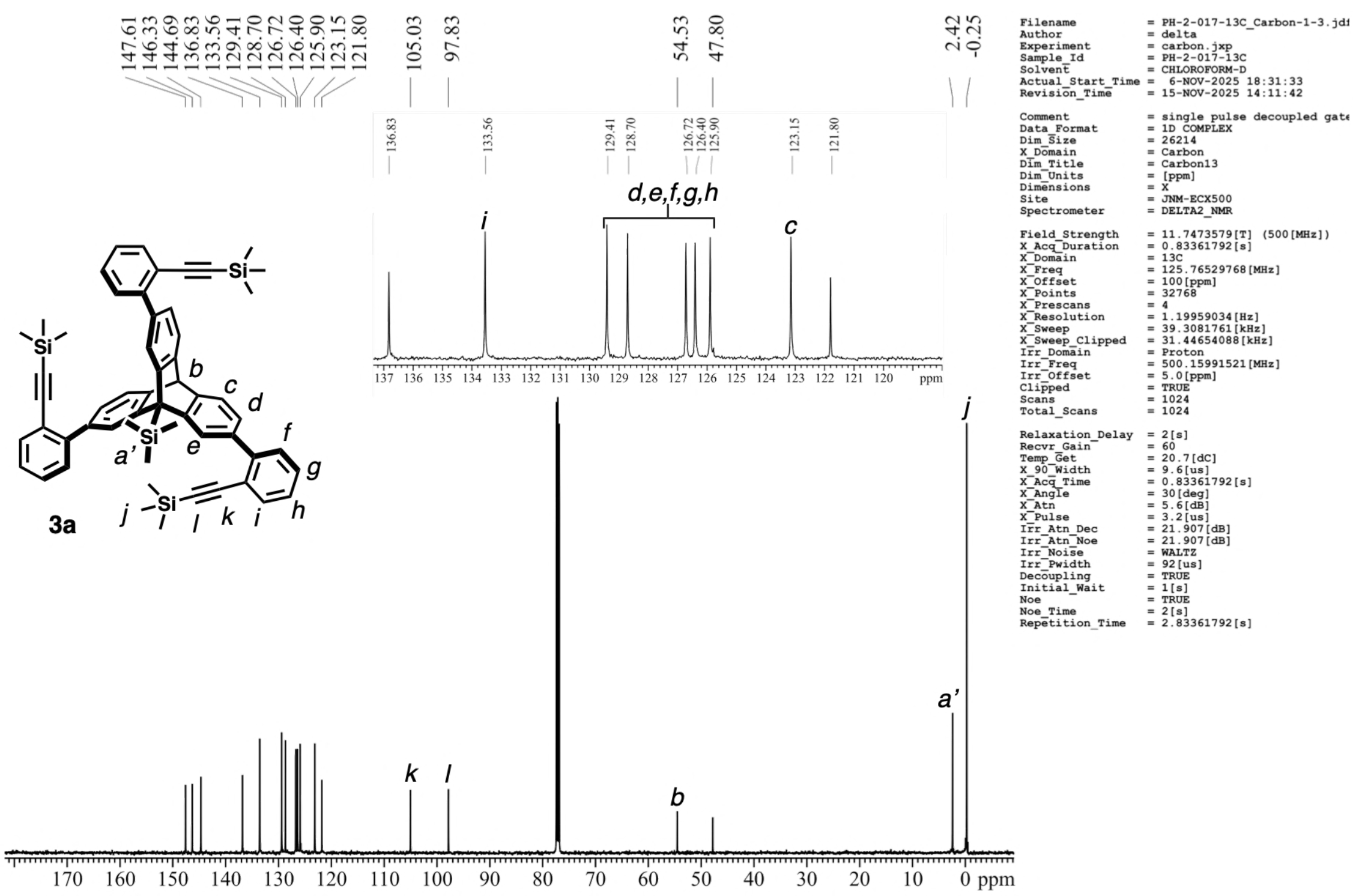

Figure S36. ^{13}C NMR spectrum (125 MHz, $CDCl_3$, 298 K) of **3a**.

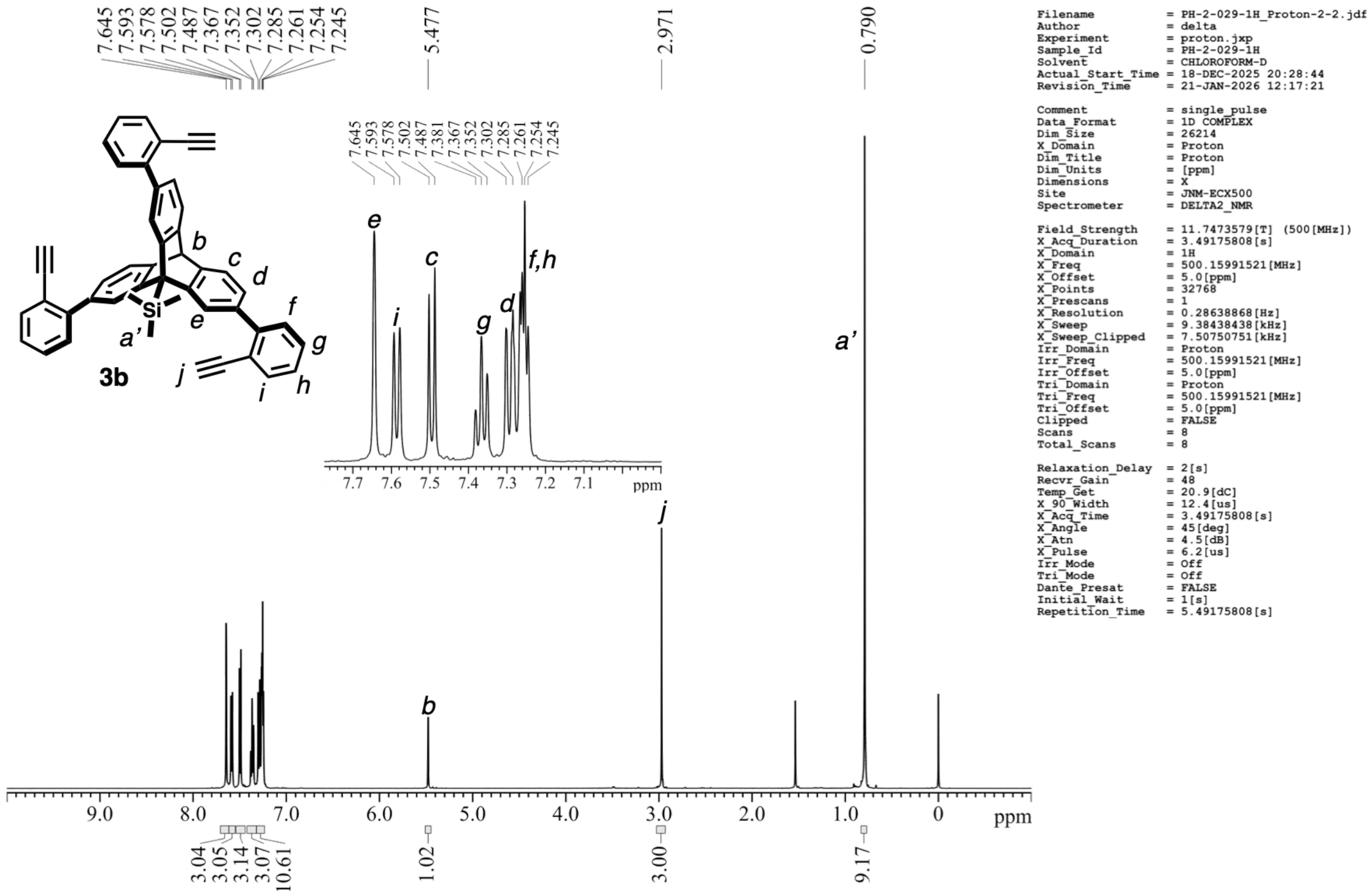

Figure S37. ^{1}H NMR spectrum (500 MHz, $CDCl_3$, 298 K) of **3b**.

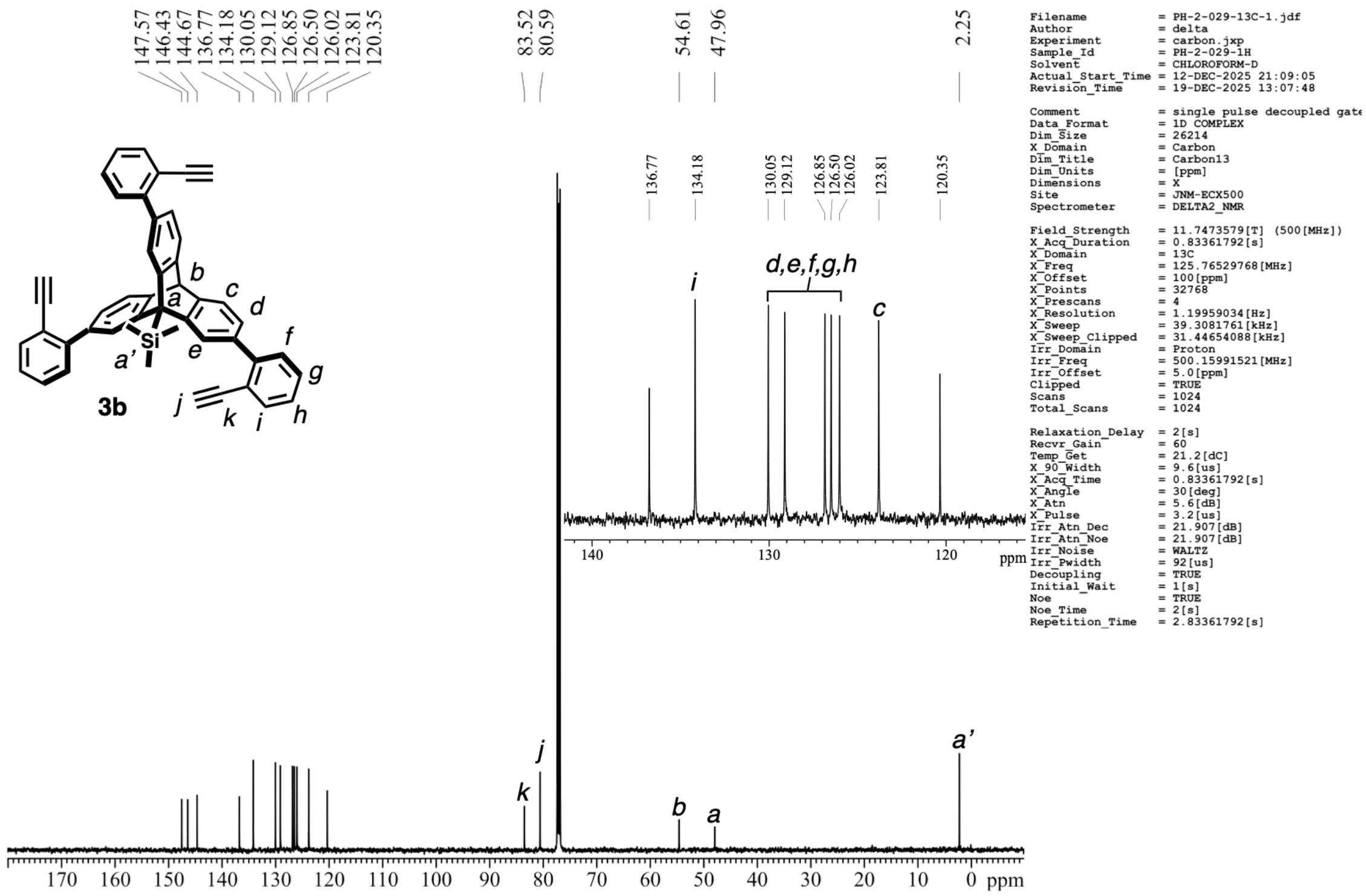

Figure S38. ^{13}C NMR spectrum (125 MHz, $CDCl_3$, 298 K) of **3b**.

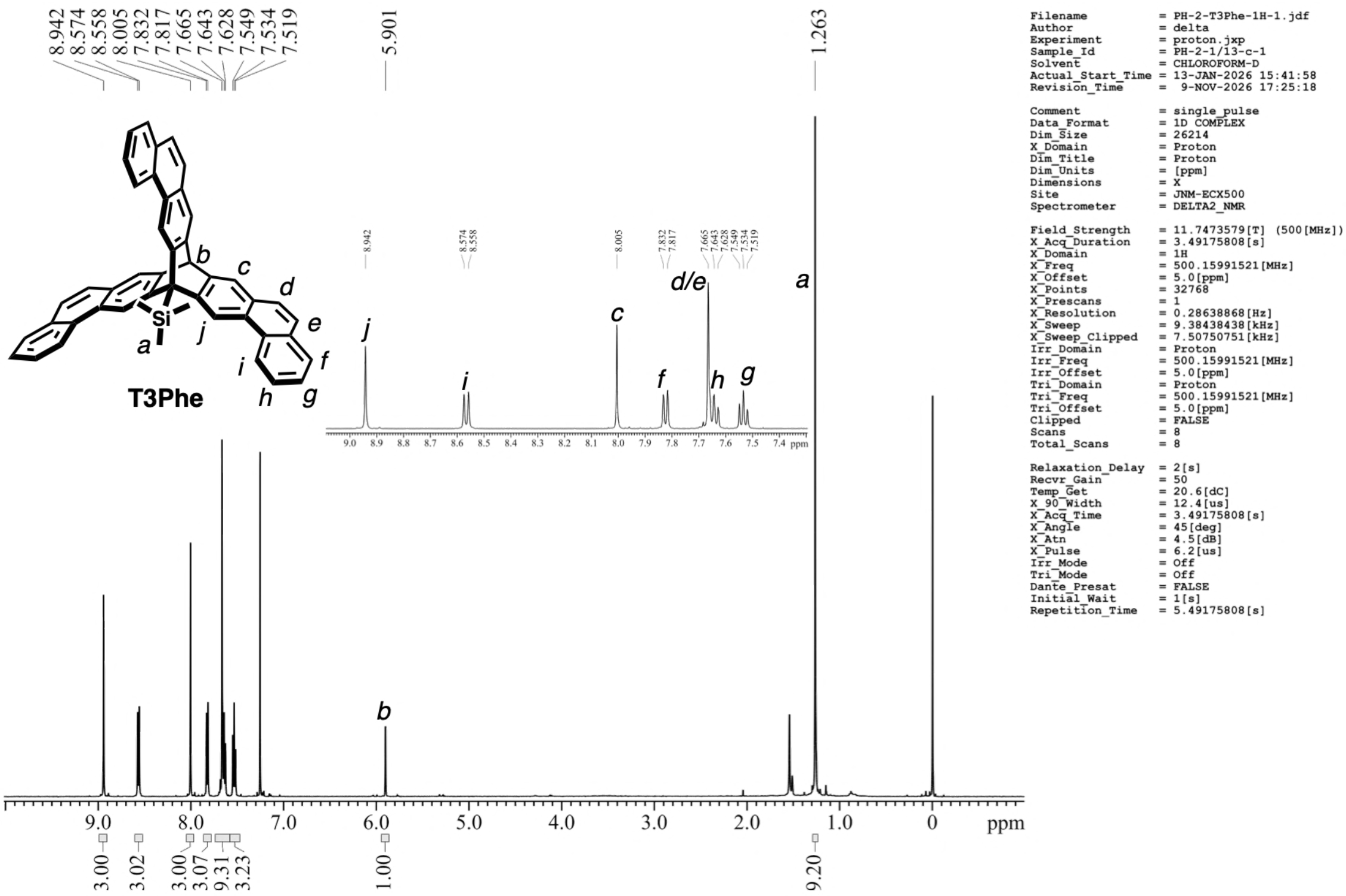

Figure S39. ^{1}H NMR spectrum (500 MHz, $CDCl_3$, 298 K) of **T3Phe**.

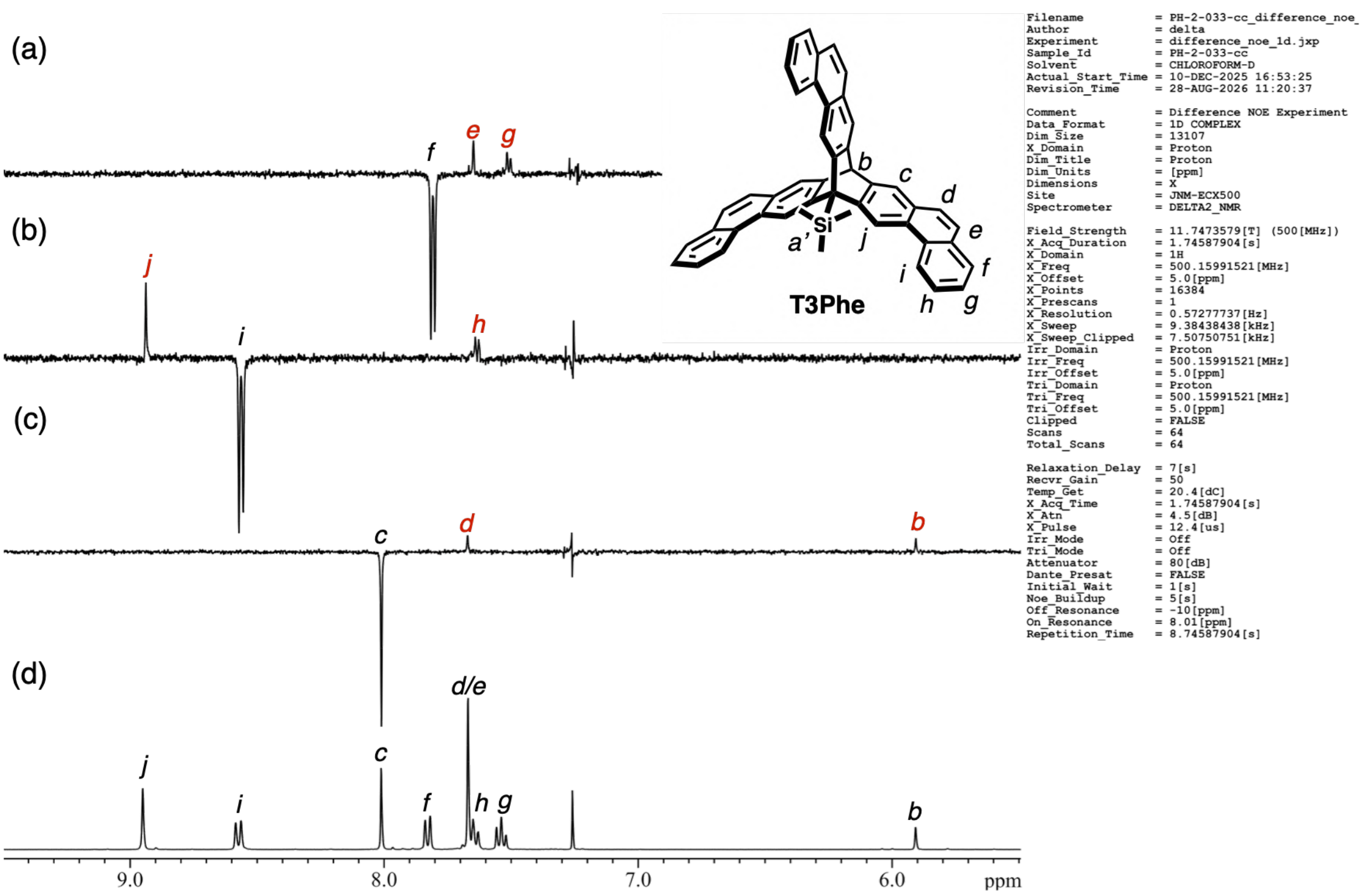

Figure S40. (a–c) 1D NOE spectra and (d) ^{1}H NMR spectrum (500 MHz, $CDCl_3$, 298 K) of **T3Phe**. Irradiation at (a) 7.83, (b) 8.57, and (c) 7.99 ppm.

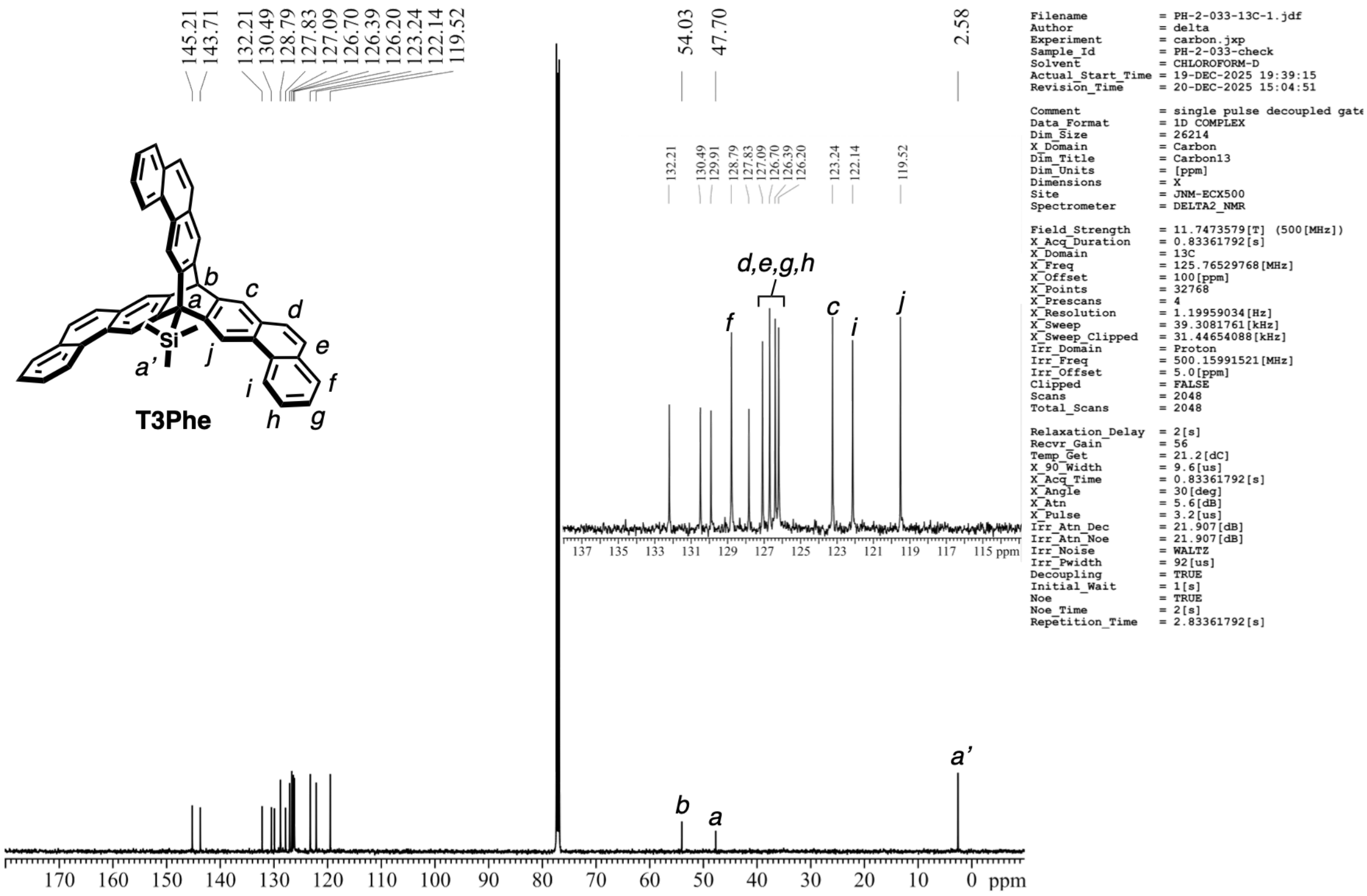

Figure S41. ^{13}C NMR spectrum (125 MHz, $CDCl_3$, 298 K) of **T3Phe**.

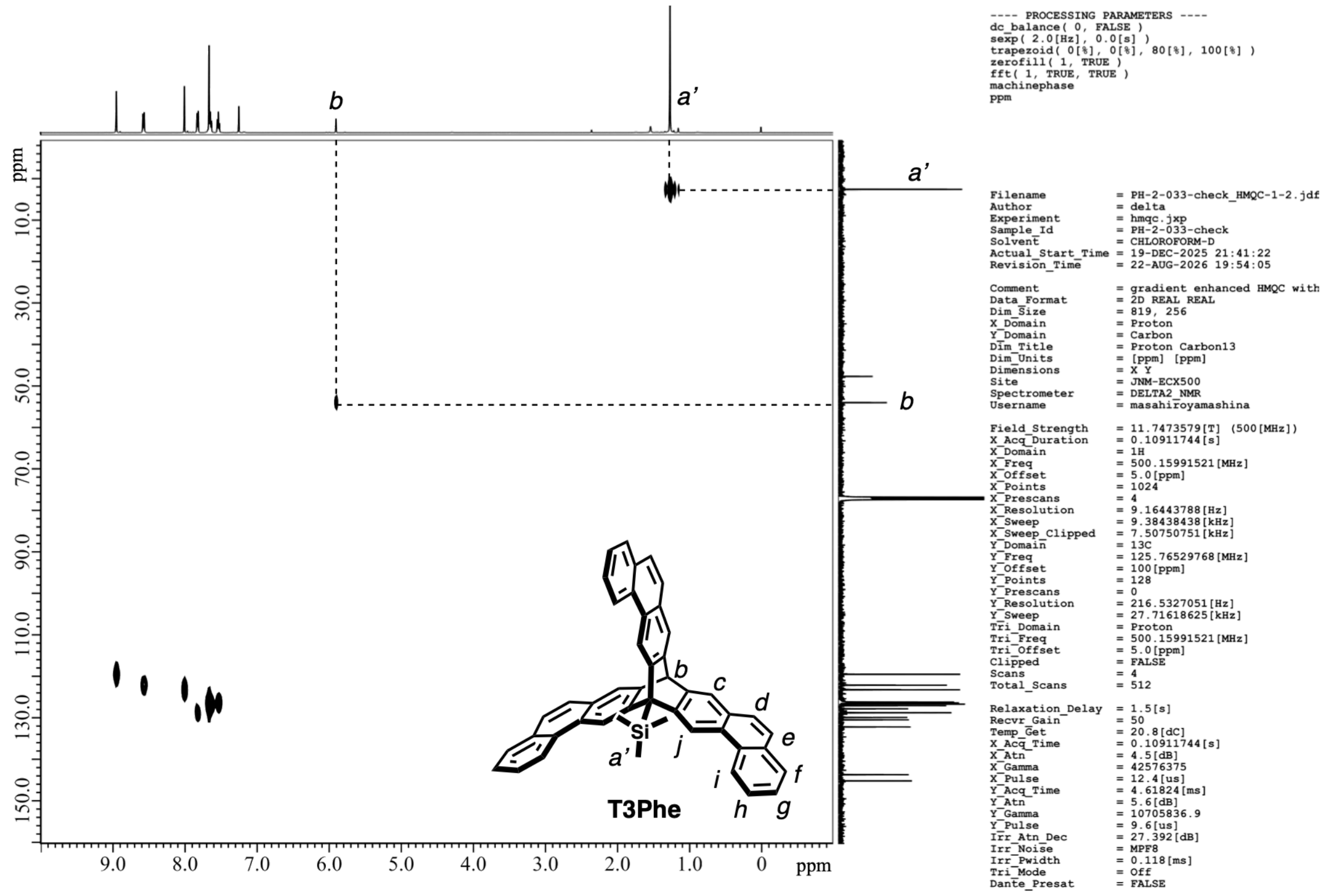

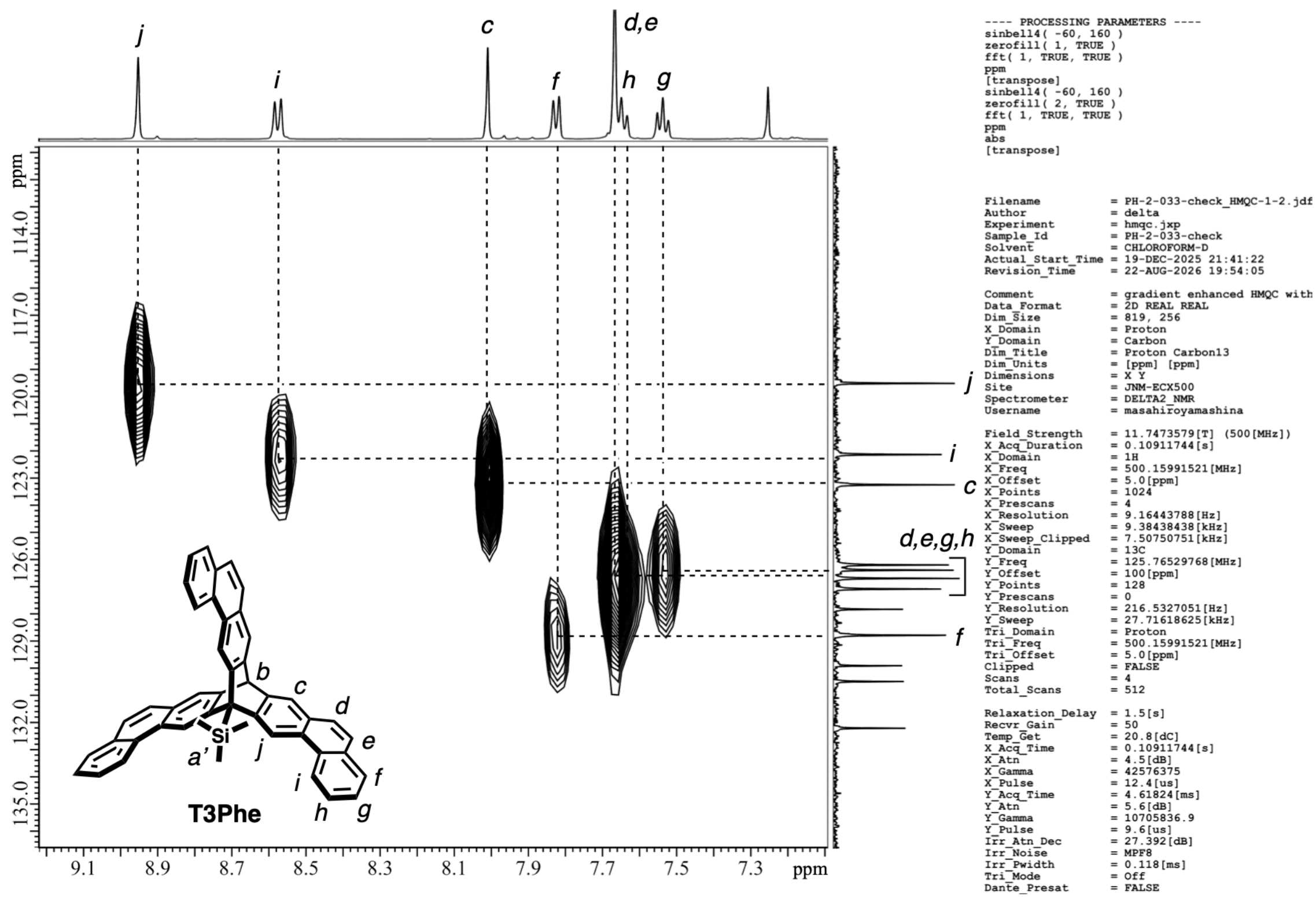

Figure S42. HMQC spectrum (500 MHz, $CDCl_3$, 5.0 mM, 298 K) of **T3Phe**. Top: –1.0−10.0 ppm region; bottom: 7.1–9.2 ppm region.

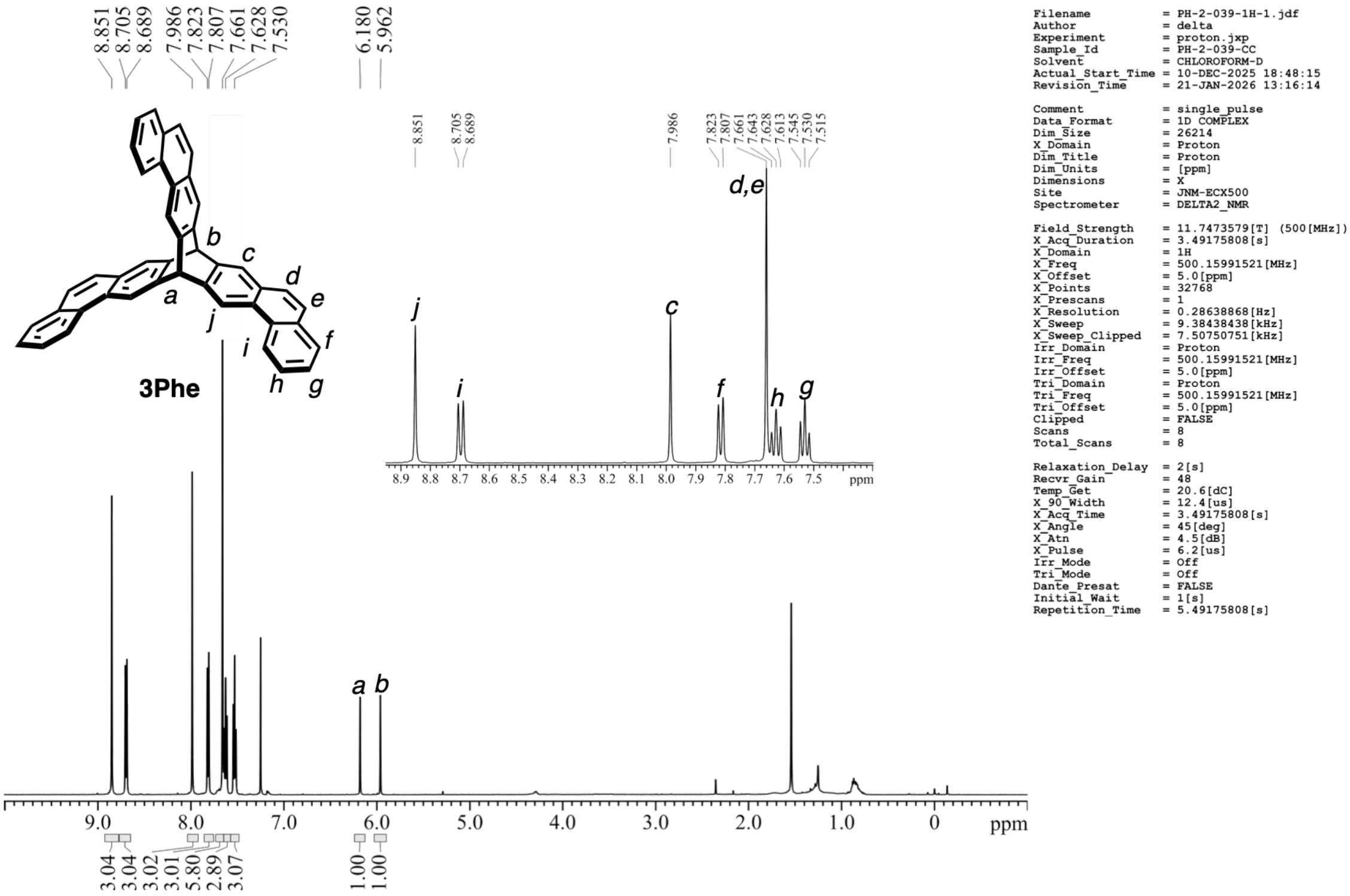

Figure S43. ^{1}H NMR spectrum (500 MHz, $CDCl_3$, 298 K) of **3Phe**.

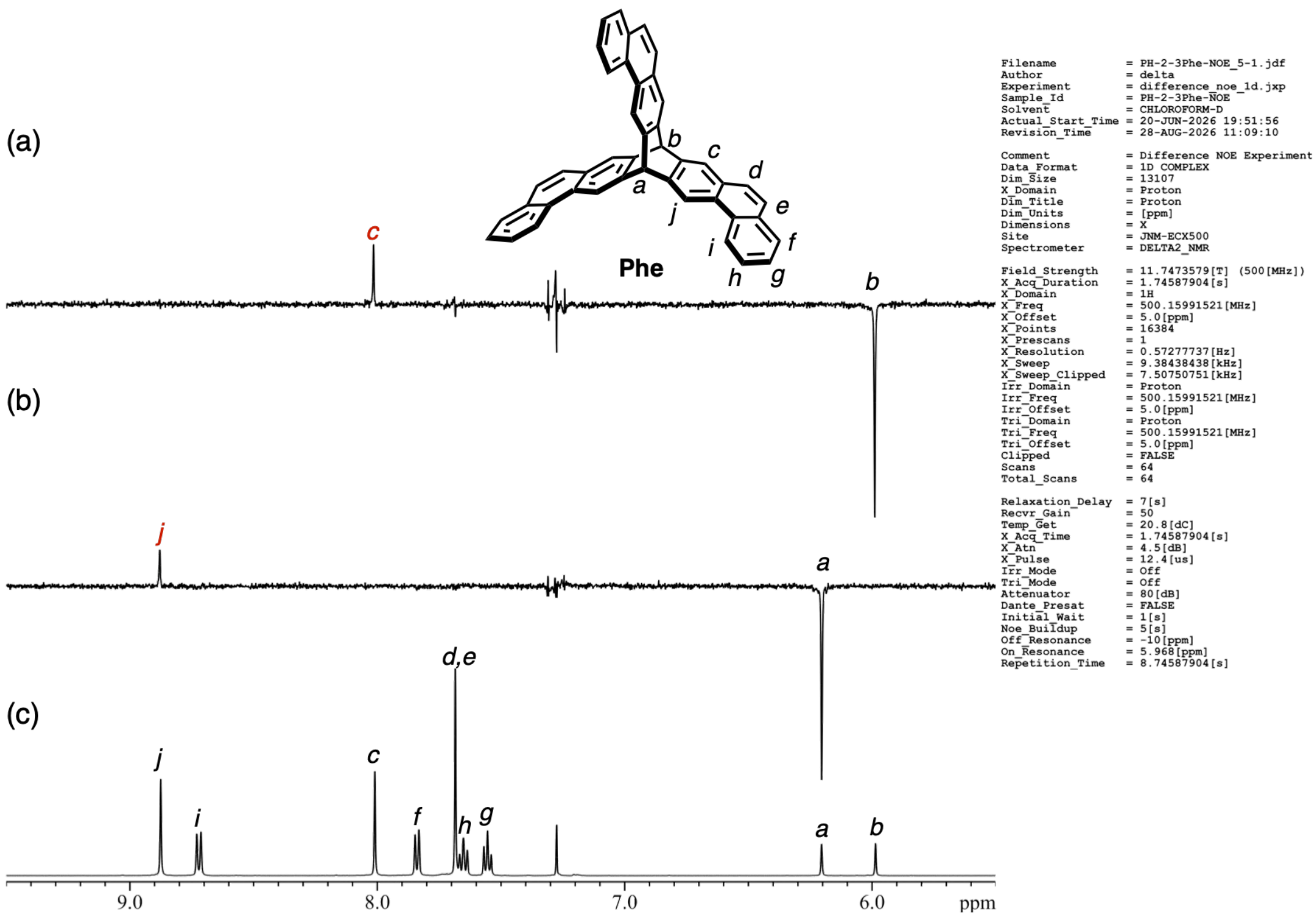

Figure S44. (a–b) 1D NOE spectra and (c) ^{1}H NMR spectrum (500 MHz, $CDCl_3$, 298 K) of **3Phe**. Irradiation at (a) 5.97 and (b) 6.18 ppm.

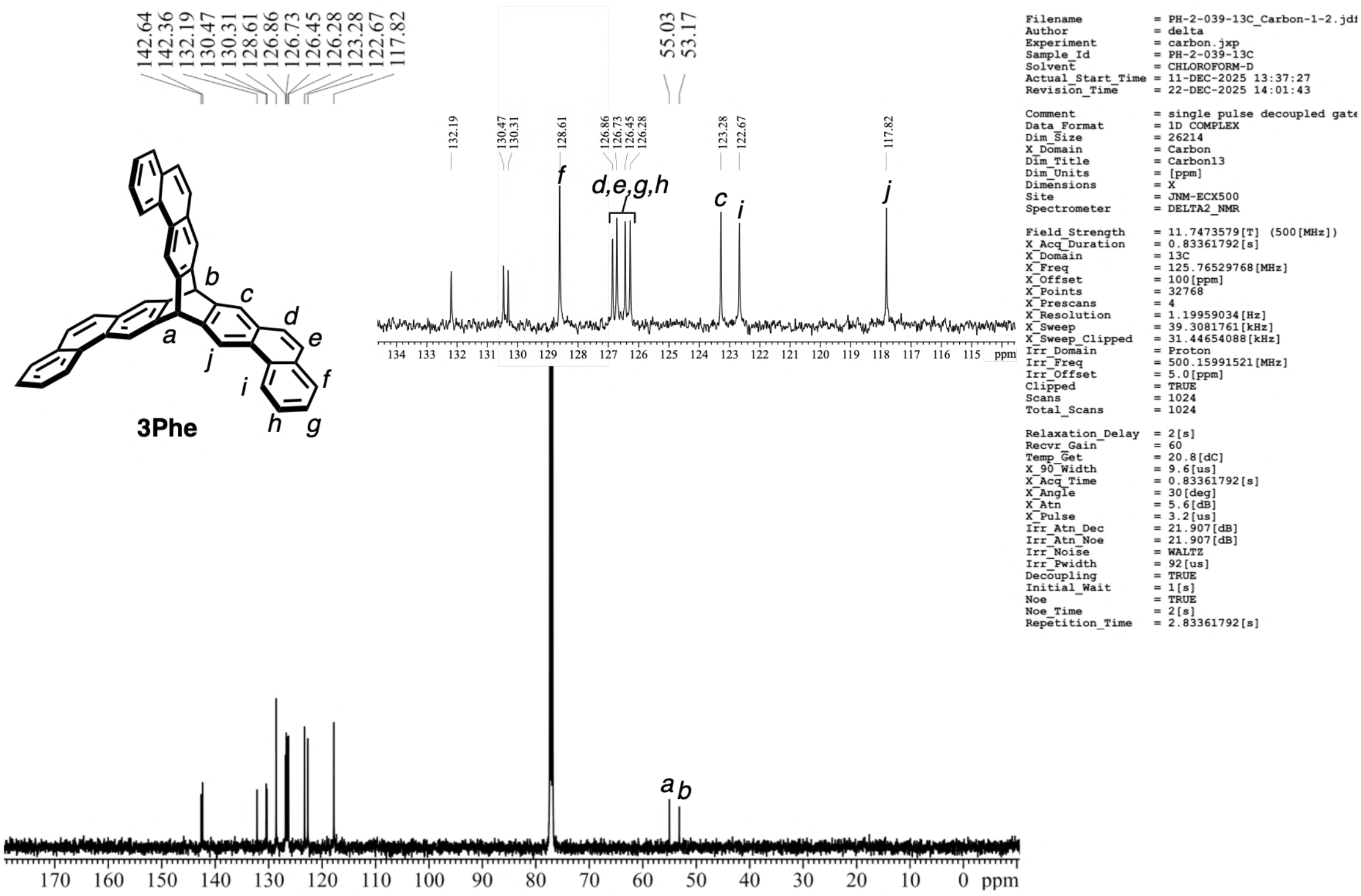

Figure S45. ^{13}C NMR spectrum (125 MHz, $CDCl_3$, 298 K) of **3Phe**.

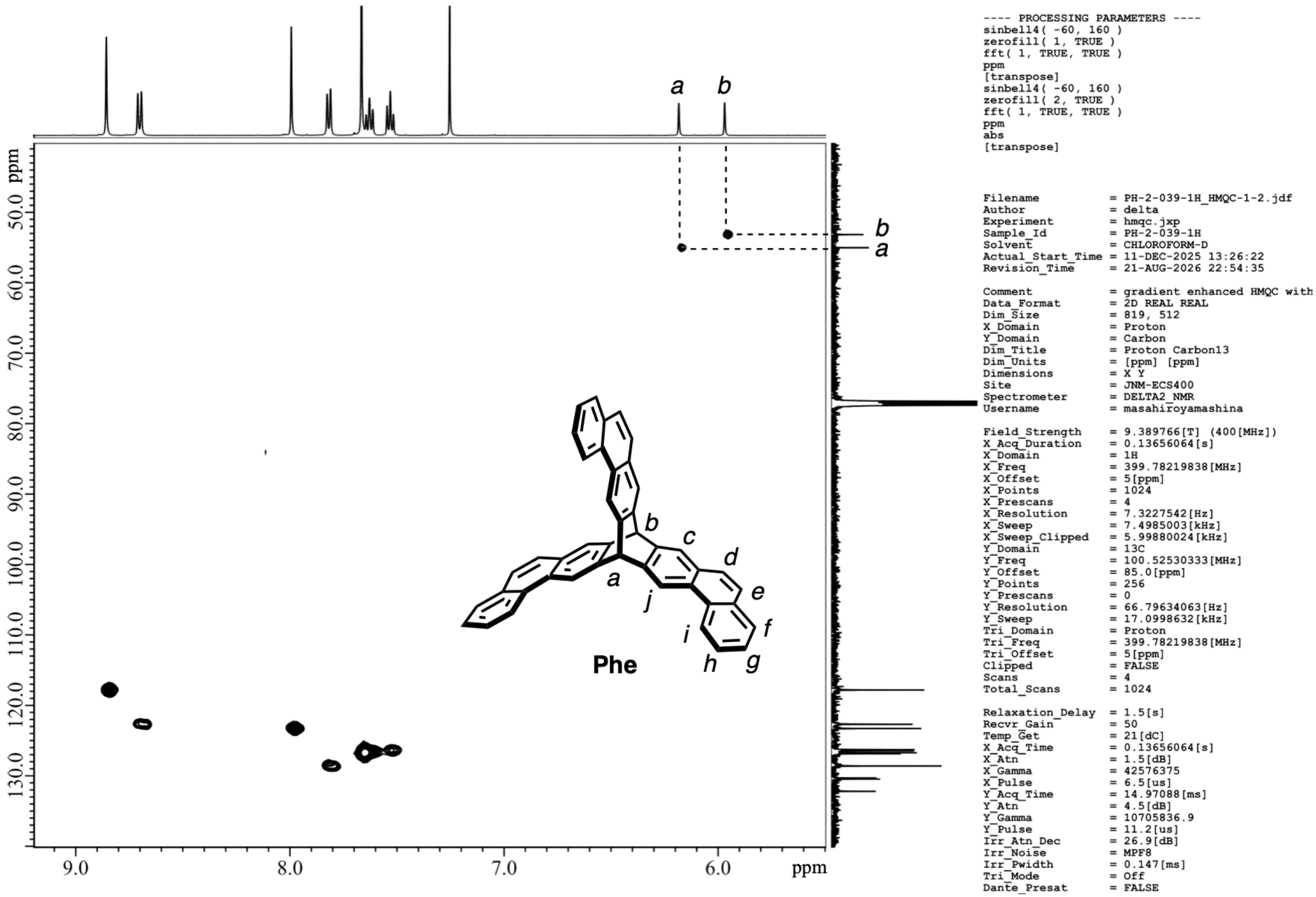

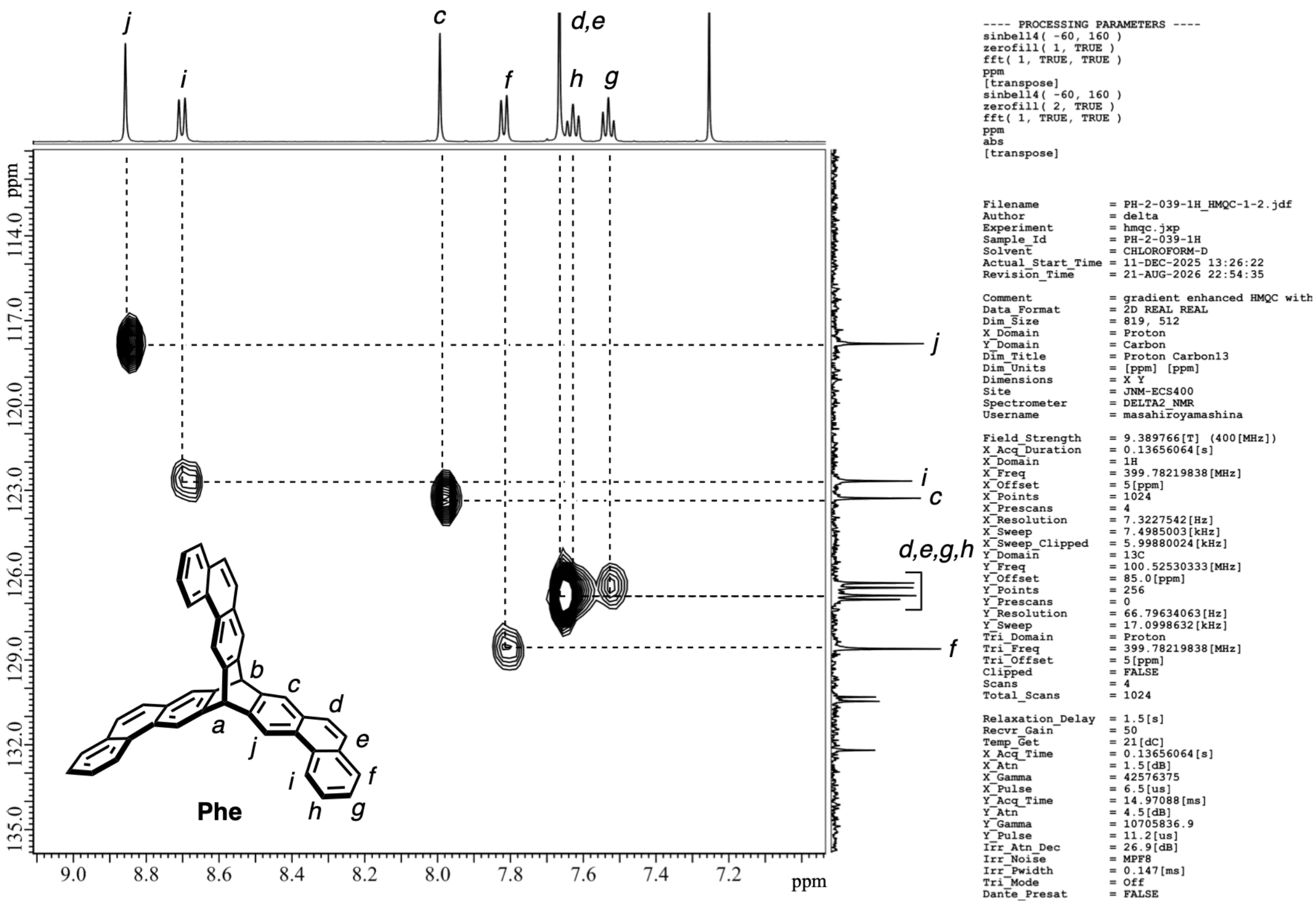

Figure S46. HMQC spectrum (500 MHz, $CDCl_3$, 5.0 mM, 298 K) of **3Phe**. Top: 5.5−9.2 ppm region; bottom: 6.9–9.1 ppm region.

8. Cartesian coordinates

Table S11. Cartesian coordinates of **Py** and **3Py** in the ground state, optimized at the B3LYP-D3(BJ)/6-31G(d,p) (PCM, $CHCl_3$) level.

Py											
C	-3.521	0.000	0.000	C	0.681	2.463	0.000	H	1.230	-3.400	0.000
C	-2.831	1.211	0.000	C	2.831	1.211	0.000	H	-1.230	3.400	0.000
C	-1.428	1.236	0.000	H	3.377	2.150	0.000	H	-4.607	0.000	0.000
C	-0.713	0.000	0.000	C	3.521	0.000	0.000	H	-3.377	2.150	0.000
C	-1.428	-1.236	0.000	C	2.831	-1.211	0.000	H	-3.377	-2.150	0.000
C	-2.831	-1.211	0.000	C	1.428	-1.236	0.000	H	1.230	3.400	0.000
C	-0.681	2.463	0.000	C	0.681	-2.463	0.000	H	4.607	0.000	0.000
C	0.713	0.000	0.000	C	-0.681	-2.463	0.000	H	3.377	-2.150	0.000
C	1.428	1.236	0.000	H	-1.230	-3.400	0.000				

3Py											
C	0.002	-0.001	0.301	C	-1.944	4.681	-2.457	H	-0.983	2.370	-3.503
C	-0.857	-1.121	-0.289	C	-2.445	5.879	2.454	H	-1.560	-2.037	-3.503
C	-0.855	-1.118	-1.693	C	-2.903	6.978	1.727	H	2.547	-0.332	-3.503
C	0.002	-0.001	-2.284	C	-2.894	6.958	0.335	H	-0.676	1.635	2.438
C	1.401	-0.184	-0.289	C	-3.100	-4.042	0.370	H	-1.501	3.615	3.599
C	1.397	-0.183	-1.693	C	-3.121	-4.070	1.797	H	-2.762	6.644	-2.353
C	-0.538	1.299	-1.693	C	-2.363	-3.082	2.513	H	-1.933	4.653	-3.542
C	-0.539	1.303	-0.289	C	-1.635	-2.135	1.863	H	-2.454	5.900	3.539
C	-1.002	2.416	0.426	C	5.053	-0.661	0.370	H	-3.269	7.855	2.251
C	-1.473	3.549	-0.317	C	5.087	-0.666	1.798	H	-3.252	7.816	-0.226
C	-1.466	3.530	-1.745	C	3.853	-0.506	2.514	H	-2.382	-3.107	3.599
C	-0.991	2.389	-2.416	C	2.668	-0.351	1.863	H	-1.076	-1.406	2.437
C	-1.573	-2.053	-2.417	C	6.268	-0.818	-0.365	H	3.883	-0.511	3.600
C	-2.326	-3.034	-1.745	C	7.475	-0.976	0.335	H	1.756	-0.234	2.438
C	-2.338	-3.051	-0.318	C	7.497	-0.979	1.728	H	8.397	-1.095	-0.226
C	-1.591	-2.077	0.426	C	6.316	-0.826	2.454	H	8.439	-1.103	2.252
C	2.596	-0.341	0.426	C	-3.846	-5.013	-0.366	H	6.339	-0.830	3.540
C	3.814	-0.499	-0.317	C	-4.588	-5.979	0.334	H	-5.154	-6.717	-0.227
C	3.793	-0.495	-1.744	C	-4.601	-5.997	1.727	H	-5.180	-6.751	2.250
C	2.567	-0.336	-2.416	C	-3.876	-5.053	2.453	H	-3.890	-5.071	3.539
C	-1.029	2.483	1.863	C	6.211	-0.810	-1.799	H	7.138	-0.931	-2.352
C	-1.489	3.586	2.514	C	5.029	-0.656	-2.456	H	4.999	-0.652	-3.542
C	-1.968	4.735	1.798	C	-3.811	-4.968	-1.800	H	-4.380	-5.710	-2.353
C	-1.954	4.703	0.370	C	-3.084	-4.023	-2.457	H	-3.066	-3.999	-3.543
C	-2.425	5.834	-0.365	H	0.001	-0.001	1.384				
C	-2.403	5.781	-1.799	H	0.002	-0.001	-3.375				

Table S12. Cartesian coordinates of **Phe** and **3Phe** in the ground state, optimized at the B3LYP-D3(BJ)/6-31G(d,p) (PCM, $CHCl_3$) level.

**Phe**											
C	0.000	-2.835	0.879	C	0.000	1.421	0.866	H	0.000	-3.441	-2.458
C	0.000	-1.421	0.866	C	0.000	2.835	0.879	H	0.000	-4.643	-0.273
C	0.000	-0.728	-0.380	C	0.000	3.558	-0.297	H	0.000	1.002	-2.531
C	0.000	-1.496	-1.567	C	0.000	2.879	-1.530	H	0.000	3.346	1.838
C	0.000	-2.879	-1.530	C	0.000	-0.680	2.094	H	0.000	4.643	-0.273
C	0.000	-3.558	-0.297	C	0.000	0.680	2.094	H	0.000	3.441	-2.458
C	0.000	0.728	-0.380	H	0.000	-3.346	1.838	H	0.000	-1.233	3.029
C	0.000	1.496	-1.567	H	0.000	-1.002	-2.531	H	0.000	1.233	3.029

**3Phe**											
C	0.000	0.001	0.574	C	-4.693	1.912	-2.140	H	2.059	1.599	-3.212
C	1.110	0.862	-0.021	C	-5.961	2.430	2.748	H	2.018	1.566	1.765
C	1.109	0.862	-1.439	C	-7.064	2.878	1.997	H	0.347	-2.530	1.765
C	0.000	0.001	-2.035	C	-7.004	2.853	0.618	H	0.354	-2.582	-3.212
C	0.191	-1.392	-0.021	C	4.035	3.131	0.703	H	-3.991	1.629	2.720
C	0.191	-1.391	-1.439	C	4.116	3.192	2.114	H	-6.663	2.714	-2.039
C	-1.301	0.531	-1.439	C	0.695	-5.060	0.703	H	-4.662	1.899	-3.226
C	-1.302	0.531	-0.021	C	0.709	-5.160	2.114	H	-6.002	2.448	3.833
C	-2.397	0.977	0.682	C	0.860	-6.258	-0.054	H	-7.954	3.241	2.500
C	-3.550	1.447	0.003	C	1.029	-7.491	0.617	H	-7.846	3.196	0.024
C	-3.544	1.445	-1.423	C	1.038	-7.556	1.996	H	3.406	2.641	2.720
C	-2.403	0.979	-2.125	C	0.876	-6.376	2.748	H	0.586	-4.270	2.720
C	2.049	1.591	-2.126	C	4.991	3.873	-0.054	H	1.153	-8.392	0.023
C	3.023	2.347	-1.424	C	5.975	4.636	0.618	H	1.169	-8.508	2.499
C	3.029	2.351	0.003	C	6.027	4.675	1.996	H	0.882	-6.421	3.833
C	2.045	1.587	0.682	C	5.086	3.945	2.748	H	6.693	5.194	0.024
C	0.352	-2.563	0.682	C	0.852	-6.201	-1.486	H	6.787	5.264	2.500
C	0.522	-3.797	0.002	C	0.690	-5.020	-2.141	H	5.122	3.972	3.833
C	0.521	-3.791	-1.424	C	4.945	3.838	-1.486	H	0.980	-7.127	-2.040
C	0.353	-2.569	-2.126	C	4.003	3.108	-2.140	H	0.686	-4.986	-3.226
C	-4.823	1.967	2.114	H	0.000	0.000	1.665	H	5.683	4.412	-2.039
C	-4.730	1.928	0.703	H	0.000	0.001	-3.126	H	3.976	3.087	-3.226
C	-5.851	2.384	-0.054	H	-2.365	0.964	1.765				
C	-5.798	2.362	-1.486	H	-2.415	0.984	-3.212				

Table S13. Cartesian coordinates of **Py** and **3Py** in the S_1 state, optimized the B3LYP-D3(BJ)/6-31G(d,p) (PCM, $CHCl_3$) level.

**Py**											
C	0.000	0.000	3.542	C	0.000	2.467	-0.689	H	0.000	-3.407	-1.233
C	0.000	1.219	2.840	C	0.000	1.219	-2.840	H	0.000	3.407	1.233
C	0.000	1.251	1.434	H	0.000	2.158	-3.386	H	0.000	0.000	4.626
C	0.000	0.000	0.697	C	0.000	0.000	-3.542	H	0.000	2.158	3.386
C	0.000	-1.251	1.434	C	0.000	-1.219	-2.840	H	0.000	-2.158	3.386
C	0.000	-1.219	2.840	C	0.000	-1.251	-1.434	H	0.000	3.407	-1.233
C	0.000	2.467	0.689	C	0.000	-2.467	-0.689	H	0.000	0.000	-4.626
C	0.000	0.000	-0.697	C	0.000	-2.467	0.689	H	0.000	-2.158	-3.386
C	0.000	1.251	-1.434	H	0.000	-3.407	1.233				

**3Py**											
C	0.191	0.000	0.297	C	-2.558	4.224	-2.479	H	-1.111	2.178	-3.529
C	-0.552	-1.182	-0.302	C	-3.544	5.196	2.419	H	-1.111	-2.179	-3.529
C	-0.512	-1.203	-1.717	C	-4.191	6.181	1.684	H	2.803	0.000	-3.472
C	0.226	0.000	-2.297	C	-4.116	6.197	0.291	H	-0.881	1.429	2.416
C	1.600	0.000	-0.274	C	-2.708	-4.190	0.340	H	-2.175	3.159	3.570
C	1.617	0.000	-1.682	C	-2.792	-4.184	1.769	H	-3.790	5.972	-2.389
C	-0.512	1.202	-1.717	C	-2.114	-3.164	2.486	H	-2.496	4.230	-3.564
C	-0.552	1.182	-0.302	C	-1.385	-2.191	1.832	H	-3.606	5.188	3.504
C	-1.287	2.166	0.416	C	5.276	0.000	0.436	H	-4.762	6.948	2.199
C	-1.960	3.184	-0.338	C	5.291	0.000	1.863	H	-4.623	6.972	-0.276
C	-1.884	3.200	-1.769	C	4.040	0.000	2.562	H	-2.176	-3.159	3.570
C	-1.148	2.179	-2.444	C	2.852	0.000	1.893	H	-0.882	-1.429	2.416
C	-1.148	-2.179	-2.444	C	6.510	0.000	-0.283	H	4.055	0.000	3.647
C	-1.884	-3.200	-1.769	C	7.718	0.000	0.433	H	1.926	0.000	2.455
C	-1.960	-3.184	-0.338	C	7.723	0.000	1.828	H	8.656	0.000	-0.115
C	-1.287	-2.165	0.416	C	6.525	0.000	2.537	H	8.666	0.000	2.363
C	2.801	0.000	0.458	C	-3.379	-5.211	-0.405	H	6.532	0.000	3.623
C	4.037	0.000	-0.267	C	-4.115	-6.198	0.291	H	-4.623	-6.972	-0.276
C	4.035	0.000	-1.697	C	-4.191	-6.181	1.684	H	-4.762	-6.948	2.199
C	2.807	0.000	-2.386	C	-3.545	-5.195	2.420	H	-3.607	-5.187	3.504
C	-1.385	2.191	1.832	C	6.473	0.000	-1.716	H	7.414	0.000	-2.258
C	-2.114	3.164	2.486	C	5.288	0.000	-2.391	H	5.274	0.000	-3.477
C	-2.792	4.184	1.769	C	-3.281	-5.198	-1.824	H	-3.790	-5.973	-2.389
C	-2.708	4.190	0.339	C	-2.557	-4.225	-2.479	H	-2.495	-4.230	-3.564
C	-3.380	5.211	-0.405	H	0.177	0.000	1.380				
C	-3.281	5.197	-1.824	H	0.246	0.000	-3.387				

Table S14. Cartesian coordinates of **Phe** and **3Phe** in the S_1 state, optimized the B3LYP-D3(BJ)/6-31G(d,p) (PCM, $CHCl_3$) level.

Phe											
C	0.000	2.847	0.890	C	0.000	-1.438	0.886	H	0.000	3.451	-2.463
C	0.000	1.438	0.886	C	0.000	-2.847	0.890	H	0.000	4.665	-0.283
C	0.000	0.714	-0.385	C	0.000	-3.581	-0.305	H	0.000	-1.010	-2.541
C	0.000	1.497	-1.574	C	0.000	-2.893	-1.532	H	0.000	-3.362	1.846
C	0.000	2.893	-1.532	C	0.000	0.699	2.089	H	0.000	-4.665	-0.283
C	0.000	3.581	-0.305	C	0.000	-0.699	2.089	H	0.000	-3.451	-2.463
C	0.000	-0.714	-0.385	H	0.000	3.362	1.846	H	0.000	1.236	3.033
C	0.000	-1.497	-1.574	H	0.000	1.010	-2.541	H	0.000	-1.236	3.033

3Phe											
C	-0.029	0.110	0.570	C	4.210	2.893	-2.122	H	0.215	-2.438	-3.256
C	0.105	-1.273	-0.043	C	5.432	3.501	2.772	H	0.233	-2.449	1.743
C	0.100	-1.265	-1.463	C	6.427	4.157	2.025	H	-2.383	1.077	1.779
C	-0.034	0.138	-2.043	C	6.355	4.159	0.645	H	-2.420	1.195	-3.203
C	-1.314	0.675	-0.016	C	0.494	-4.982	0.664	H	3.634	2.363	2.734
C	-1.313	0.699	-1.437	C	0.513	-5.094	2.063	H	6.012	4.029	-2.011
C	1.122	0.923	-1.440	C	-4.753	2.042	0.731	H	4.173	2.902	-3.207
C	1.131	0.898	-0.019	C	-4.856	2.037	2.140	H	5.484	3.494	3.856
C	2.144	1.516	0.691	C	-5.870	2.521	-0.020	H	7.247	4.657	2.531
C	3.191	2.185	0.019	C	-7.024	2.973	0.658	H	7.117	4.661	0.056
C	3.171	2.217	-1.416	C	-7.093	2.958	2.038	H	0.423	-4.203	2.675
C	2.119	1.576	-2.123	C	-5.998	2.484	2.783	H	-4.029	1.677	2.741
C	0.218	-2.439	-2.171	C	0.617	-6.192	-0.115	H	-7.863	3.335	0.070
C	0.350	-3.689	-1.480	C	0.750	-7.433	0.567	H	-7.986	3.308	2.546
C	0.357	-3.698	-0.021	C	0.765	-7.502	1.951	H	-6.046	2.468	3.867
C	0.232	-2.480	0.660	C	0.646	-6.326	2.711	H	0.842	-8.339	-0.025
C	-2.417	1.106	0.696	C	-5.809	2.536	-1.449	H	0.868	-8.463	2.445
C	-3.567	1.581	0.026	C	-4.699	2.099	-2.113	H	0.656	-6.369	3.795
C	-3.556	1.614	-1.408	C	0.603	-6.125	-1.520	H	-6.671	2.904	-1.999
C	-2.411	1.167	-2.117	C	0.472	-4.897	-2.183	H	-4.666	2.113	-3.198
C	4.384	2.861	2.131	H	-0.027	0.098	1.661	H	0.696	-7.043	-2.093
C	4.280	2.846	0.722	H	-0.037	0.152	-3.134	H	0.464	-4.869	-3.269
C	5.295	3.513	-0.032	H	2.118	1.481	1.774				
C	5.230	3.517	-1.460	H	2.121	1.605	-3.209				
